# A scoping review: What are the cellular mechanisms that drive the allergic inflammatory response to fungal allergens in the lung epithelium?

**DOI:** 10.1002/clt2.12252

**Published:** 2023-06-01

**Authors:** Emma‐Jane Goode, Emma Marczylo

**Affiliations:** ^1^ Toxicology Department UK Health Security Agency Chilton UK

**Keywords:** allergic, epithelial, fungi, IL33, protease

## Abstract

Allergic airway disease (AAD) is a collective term for respiratory disorders that can be exacerbated upon exposure to airborne allergens. The contribution of fungal allergens to AAD has become well established over recent years. We conducted a comprehensive review of the literature using Preferred Reporting Items for Systematic Reviews and Meta‐Analyses guidelines to better understand the mechanisms involved in the allergic response to fungi in airway epithelia, identify knowledge gaps and make recommendations for future research. The search resulted in 61 studies for final analysis. Despite heterogeneity in the models and methods used, we identified major pathways involved in fungal allergy. These included the activation of protease‐activated receptor 2, the EGFR pathway, adenosine triphosphate and purinergic receptor‐dependent release of IL33, and oxidative stress, which drove mucin expression and goblet cell metaplasia, Th2 cytokine production, reduced barrier integrity, eosinophil recruitment, and airway hyperresponsiveness. However, there were several knowledge gaps and therefore we recommend future research should focus on the use of more physiologically relevant methods to directly compare key allergenic fungal species, clarify specific mechanisms of fungal allergy, and assess the fungal allergy in disease models. This will inform disease management and future interventions, ultimately reducing the burden of disease.

## 1 INTRODUCTION

### 1.1 Fungal allergens

Fungi are eukaryotic organisms that are ubiquitous in the environment and can be present in the air we breathe as bioaerosols.^1^, ^2^ Humans are exposed to fungi multiple times on a daily basis with the main route of exposure being inhalation.^3^, ^4^ Most fungi cause little adverse effects in healthy humans, but a minority can cause invasive disease or respiratory allergy, especially in susceptible individuals.^5^, ^6^ Fungal species known or thought to cause respiratory allergy and contribute to the pathogenesis of respiratory disease can be found in Table 1.^7^ Thermotolerant genera such as *Aspergillus* and *Penicillium* may exert their allergenic effects through colonisation of the airway, whilst non‐thermotolerant fungi such as *Alternaria* may still cause an allergic response through inhalational exposure and sensitisation.^11^, ^12^

**TABLE 1:** Known thermotolerant^a^ and non‐thermotolerant fungal allergens.^7^, ^8^, ^9^, ^10^

Genus	Species	Thermotolerant/ non‐thermotolerant	Indoor/ outdoor/ commensal^b^	No. of allergens identified^c^	Peak season^d^
***Alternaria***	***alternata***	Non‐thermotolerant	Outdoor	12	Mid‐July–mid September
***Aspergillus***	*clavatus*	Thermotolerant	Indoor/outdoor	0	Late August–October, January–February
	***fumigatus***			30	
	*niger*			3	
	*oryzae*			2	
*Candida*	*albicans*	Thermotolerant	Commensal	2	
***Cladosporium***	*cladosporioides*	Non‐thermotolerant	Outdoor/indoor	2	June–mid‐September
	*herbarum*			8	
	*sphaerospermum*			0	
*Curvularia*		Non‐thermotolerant	Outdoor	0	
*Epicoccum*	*nigrum*	Non‐thermotolerant	Outdoor/indoor	0	July–September
	*purpurascens*			2	
***Penicillium***	*chrysogenum*	Thermotolerant	Indoor	6	Late August–October, January–February
	*citrinum*			7	
*Pleospora*		Non‐thermotolerant	Outdoor	0	Late March to early June

*Note*: Genus and species highlighted in bold are predominantly looked for in sampling and epidemiology studies.

^a^ Thermotolerant refers to the ability to grow at 37°C.

^b^ Where there is evidence that spores are present in both environments, the predominant environment is listed first.

^c^ Allergens that are officially approved and listed by the Nomenclature Subcommittee of the International Union of Immunological Societies http://www.allergen.org/committee.php.

^d^ Spore season may be longer than listed and is dependent on weather conditions.

Fungi have specific features that are recognisable to the human immune system, termed pathogen‐associated molecular patterns (PAMPs) and include β‐glucan, chitin and mannans. In addition, fungi produce proteases and glycosidases to degrade material for nutrient release, which can also degrade epithelial tight junctions in the lung and induce allergic responses.^1^, ^4^, ^11^, ^13^

### 1.2 Allergic airway disease

Allergic airway disease (AAD) is a collective term for respiratory disorders that can be exacerbated upon exposure to airborne allergens, including fungal agents, pollen and house dust mite (HDM).^11^ These disorders include asthma, chronic rhinosinusitis (CRS) and allergic bronchial pulmonary aspergillosis (ABPA), which affect millions of people globally.^1^, ^14^, ^15^, ^16^, ^17^

Typically, AAD is defined as an allergic response in individuals characterised by increased serum IgE, airway inflammation, airway remodelling (including peribronchial fibrosis and increased collagen deposition), increased type 2 cytokine profile (e.g. Interleukin (IL) 4, IL5, IL13, IL25, IL33, thymic stromal lymphopoietin (TSLP)), airway eosinophilia, mucus hypersecretion, and airway hyperresponsiveness (AHR).^1^, ^13^, ^18^

Upon inhalation, the airway epithelium is the first cellular barrier that fungal allergens encounter and thus has important protective functions, including a physical barrier to inhaled particles and microbes, immunological functions and mucociliary clearance.^18^, ^19^, ^20^, ^21^ Allergens can become trapped in the nasal cavity, trachea, bronchi, small airways, and alveoli^5^, ^22^, ^23^ depending on their size, with the majority of particles being deposited within the nasal cavity and upper airways (due to size and turbination of the air), and some smaller particles (<5 μm) penetrating into the smaller airways.^4^ The upper airway has a columnar pseudostratified epithelium made up of cilia, goblet cells and/or club cells and basal cells,^20^, ^21^ whilst the smaller airways and terminal bronchioles are more cuboidal.^24^ Various structural and functional differences exist between the large and small airways.^24^ Goblet cells produce mucus, which in combination with the cilia, trap particles that impact the epithelium and move them up the airway to the mouth via the mucociliary escalator.^20^ Pattern recognition receptors (PRRs) on the epithelium can detect fungal allergens/PAMPs or danger associated molecular patterns (DAMPs), triggering signalling cascades that induce the release of several cytokines and chemokines^14^, ^19^, ^21^, ^25^ that drive allergic responses.

The prevalence of allergic respiratory conditions has increased over the last few decades^4^, ^26^ and fungal allergens can play a significant role in the development of AAD^4^ with several species of fungi also recognised as ‘sensitisers’.^1^, ^3^, ^27^ Sensitisation refers to the process by which the body becomes abnormally sensitive to an allergen through repeated exposures and is the first stage in the development of allergy.^28^ Repeated inhalation of fungal allergens can contribute to the development and exacerbations of asthma and allergic rhinitis.^29^ In particular, exacerbations of allergic asthma have been linked with fungal spore exposure^4^, ^29^ and there is a correlation between severe asthma and fungal sensitisation^17^ with 35%–75% of severe asthmatics having some form of fungal sensitisation (compared to the general population with a rate of 3%–10%).^7^, ^27^ A 2007 review attributed approximately 4.6 million cases of asthma in the United States to indoor dampness and mould exposure alone, with an economic impact of approximately $3.5 billion annually,^30^ and in the United Kingdom, it is estimated that 5.4 million people have asthma (including 1.1 million children).^27^, ^31^ A 2014 position paper posited that potentially >6.5 million people globally suffer from severe asthma with fungal sensitisation.^29^

Understanding the biological processes driving the contribution of fungal allergens to AAD is essential for improving disease management, treatment strategies, interventions, and ultimately reducing the burden of disease.

### 1.3 Aim

This review aimed to collate the current understanding of mechanisms involved in the allergic response to fungi in the airway epithelium, identify knowledge gaps and make recommendations for future research.

## 2 METHODS

### 2.1 Search strategy

A comprehensive Preferred Reporting Items for Systematic Reviews and Meta‐Analyses (PRISMA)‐based literature search was conducted using the PubMed database in January 2022. The search string comprised fungal, allergy, respiratory and epithelial/macrophage search terms (Supporting Information S1).

### 2.2 Study selection

The selection of studies occurred in three stages: first by titles, then by abstracts and then by full text. All titles and abstracts were reviewed independently by two reviewers. Table 2 details the inclusion and exclusion criteria. Only data from relevant studies within the last 15 years were extracted at the full text stage to focus on the most current literature and avoid repetition of information. However, these papers were checked for any relevant data and referenced in the discussion where appropriate.

**TABLE 2:** Inclusion/exclusion criteria for selected studies.

Inclusion criteria	Exclusion criteria
Written in English Contained original peer‐reviewed research Referred to human or rodent (rat, mouse) models. Focused on respiratory (nasal, bronchial/tracheal, alveolar) epithelial cells or lung‐resident tissue macrophages Focused on respiratory allergy and/or allergic lung inflammation from fungal allergens^a^ Included one or more of the following fungal allergens—*Alternaria* spp., *Cladopsorium* spp., *Aspergillus* spp., *Epicoccum* spp., *Penicillium* spp., *Didymella* spp., *Pleospora* spp., *Sporobolomyces* spp., *Tilletiopsis* spp.^b^	Review papers, book chapters or letters Focused on non‐allergic fungal disease (infection, immune modulation, clinical presentation etc). Focused on fungal biology, antifungal medication or therapeutics Focused on immune cells not present at the air interface under normal circumstances (i.e. migratory immune cells such as neutrophils, eosinophils and dendritic cells) or other lung resident tissues (i.e. lung fibroblasts or smooth muscle) Focused on other body systems Used fungal allergens as part of the triple allergen (DRA)^c^ sensitisation procedure rather than fungi as the experimental focus. Used other types of macrophage rather than alveolar macrophages Only used OVA as an allergen model and did not include a fungal allergen

Abbreviation: OVA, ovalbumin.

^a^ Includes allergic respiratory disorders that is, asthma, chronic obstructive puolmonary disease (COPD), cystic fibrosis, acute and chronic rhinitis, and allergic bronchopulmonary aspergillosis /mycosis.

^b^ Studies containing other fungal species were considered provided there was evidence they caused allergy.

^c^ DRA = dust mite, ragweed, *aspergillus*.

### 2.3 Data extraction

Using a pre‐designed template, the following study data were extracted: author(s) and year, fungal allergens, potential target mechanisms, experimental models, and experimental outcomes. Only data relating to fungal allergens and epithelial cells were extracted. Other exposure compounds or cell types were noted when relevant to fungal allergen activity (either for confirmation of allergic models or when related to events surrounding the epithelium). Potential target mechanisms were defined as any molecule or pathway that was either (a) the specific focus of the study, (b) a molecule or pathway that was further validated using knockouts (KOs), inhibitors or gene silencing/overexpressing techniques, or (c) an endpoint molecule where pathways or genes were manipulated to observe the effects on said molecule. Molecules that were investigated using appropriate validation techniques but found not to be involved in specific pathways were still included as potential target mechanisms. Molecules that were used as endpoints to confirm allergic responses in models were not included as potential target mechanisms unless they underwent further investigation.

### 2.4 Quality scoring

A quality‐scoring tool was adapted from a previous review.^27^ This enabled the assessment of seven domains deemed relevant for understanding the mechanisms of fungal exposure, providing an overall quality score for each study. A detailed explanation of the quality‐scoring tool is available in Supporting Information S2. Briefly, the seven domains were:

- route of exposure: physiologically relevant applications of fungal exposures. Higher scores were given if fungal allergens were applied apically to cells in ALI systems (in vitro) or if fungal allergens were applied via an inhalational route (in vivo).
- cell model specificity: physiologically relevant models, tissues, or cells of the airways. Higher scores were given if cells or ex‐vivo tissues were primary human epithelial cells or tissues (either derived directly from patient specimens or purchased) for in vitro studies, or if the study focused on lung epithelial cells or lung epithelial tissues from animal models (in vivo).
- confirmation of targets: using inhibitors/agonists. Higher scores were given if studies confirmed targets using a combination of activating and inhibiting compounds or techniques.
- gene and protein expression: confirming gene expression changes with protein expression assays. Higher scores were given if studies used a combination of protein expression methods to follow‐up gene expression results.
- Th2 responses: measuring specific Th2 cytokines. Higher scores were given if studies measured known Th2 cytokines (IL‐4, IL‐5, IL‐13, IL25, IL33 or TSLP).
- cytotoxicity: measuring cytotoxicity after application of fungal allergens (*in vitro* only). Higher scores were given if studies used assays to determine whether the application of fungal allergens caused cytotoxicity in cells.
- induction of allergy: physiologically relevant methods of fungal sensitisation and challenge (in vivo only). Higher scores were given if protocols used consisted of fungal sensitisation through an inhalational route followed by fungal challenge, and allergic inflammation was confirmed in the model.

Scores for each domain were classified as high, moderate, low, or very low. Each study was scored individually against the criteria for each domain. Only the data that was extracted for the final analysis was quality assessed. These scores were then averaged, giving each domain and study an overall quality score.

## 3 RESULTS

### 3.1 Overview of included studies

**FIGURE 1:**
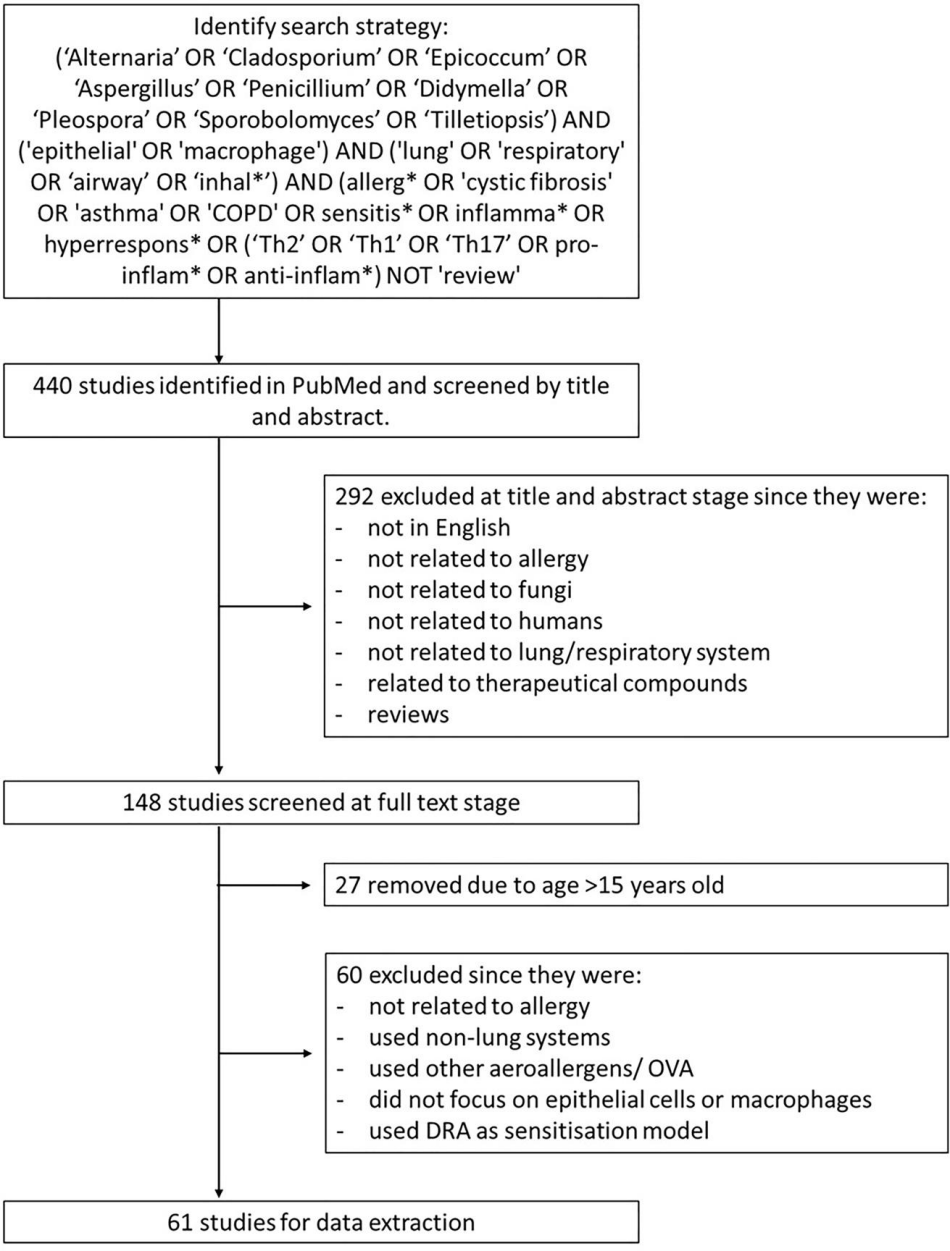
Flow chart summarising the selection process for studies identified through database searches. The number of studies was limited based on age; therefore, studies older than 15 years were discarded to stay on top of current literature and avoid repetition of information. However, studies older than 15 years were included in the wider literature where appropriate and where more recent evidence was lacking. COPD, chronic obstructive pulmonary disease; DRA, dutmite ragweed *aspergillus*; OVA, ovalbumin.

The search string provided 440 results from the PubMed database. Study selection according to PRISMA guidelines is outlined in Figure 1, which resulted in 61 studies for data extraction (Table 3). See Supporting Information S3 for further details of each study.

**TABLE 3:** Overview of included studies including experimental model, fungal species and outcomes. A more detailed version of this table is available in Supporting Information S4.

Author(s)	Fungus/fungi	Outcomes
Anagnostopoulou et al., 2010^32^	*Aspergillus fumigatus* extract	Allergic inflammation reduced α, β, *γ* ENaC expression, but reduction abolished in STAT6^−/−^ mice Bronchial tissues were more absorptive under normal conditions cAMP mediated Cl^−^ secretion was attenuated by allergic inflammation STAT6 was directly involved in expression of epithelial ion transport channels in AAD
Babiceanu et al., 2013^33^	*A. alternata* conidia	AA induced selective activation of different innate immunological pathways in ECs Multiple functional related chemokines and cytokines and related signalling pathways AA proteins and/or secreted metabolites were potent induces of inflammation
Bains et al., 2012^34^	*A. fumigatus* (mycelia extract and culture filtrate)	Demonstrated loss of Cav‐1 in asthmatics versus to controls Evidence of hypercellularity and peri‐bronchial fibrosis observed in AF‐sensitised mice versus control mice Staining for Cav‐1 decreased in AF sensitised mice versus control mice
Bankova et al., 2016^35^	*A. alternata* culture filtrate	AA induced marked swelling, no detectable cellular infiltration, increased mucus secretion in WT mice versus controls LTC_4_ KO reduced swelling and mucin release Submucosal oedema and mucin release were absent in AA‐treated mast cell‐deficient mice but present in AA‐treated *Fcer1g* ^−/−^ mice Absence of submucosal swelling and reduced mucin release in *Cysltr1* ^*−/−*^ but intact in *Cystlr2* ^*−/−*^ mice versus WT Submucosal swelling and mucin release inhibited in *gpr99* ^*−/−*^ mice versus WT GPR99 was the dominant CysLTR for LTE4, elicited EC secretory function
Bickford et al., 2012^36^	*A. fumigatus* extract	AF sensitisation caused Th2 responses, goblet cell hyperplasia, airway eosinophilia, increased total serum IgE IFNγ, TNFα, IL1B, IL6, IL10, MCP1, MIP‐1α were elevated cPLA2y (phospholipase A) was induced in a model of allergic asthma (cytosolic phospholipase) via TNFα and NF‐κB, in response to fungal sensitisation
Boitano et al., 2011^37^	*A. alternata*—culture filtrate (also heat‐inactivated [HI‐AA])	AA sensitisation caused a 16‐fold increase in BALF cells, increase in lymphocytes, neutrophils, macrophages and eosinophils HI‐AA & serine protease inhibitor reduced cell counts in BALF Ca^2+^ response started 20–45 s following application of AA PAR2 had highest level of expression versus PAR1, PAR3 & PAR4 in ECs AA serine specific protease activity was required to develop lung inflammation & cell recruitment to airways Serine‐specific proteases were responsible for AA‐induced Ca^2+^ signalling in ECs
Brandt et al., 2008^38^	*A. fumigatus* (form unknown)	*Sftpd* KO mice: Increased BALF cells including increased macrophages and activated T‐cells. No change seen in Th1 and Th2 cytokine levels. Increased CCL17 and CCL2 but IgE and IgG1 lower. SPD deficiency was not associated with decreased IFNγ or increased IL4. Small increase in TNFα AF exposure: Decreased total BALF cells including eosinophils following exposure in KO mice versus WT. IgE undetectable, IgG1 no significant difference in null versus WT. IL13 increased in WT and KO mice versus saline controls but IL13 lower in KO mice versus WT Clinical data shows polymorphism in surfactant protein D at position 11, which can reduce risk of asthma susceptibility *Sfptd* KO increased chemokine expression and SPD deficiency attenuated development of Th2 responses
Buckland et al., 2011^39^	*A. fumigatus* conidia and AF antigens as indicated	AHR and GCM were enhanced when TREM‐1 blocked but reduced in DAP12 overexpression group versus controls. Increased collagen deposition and peri‐bronchial inflammation when TREM‐1 was blocked but inflammation decreased with DAP12 overexpression. TREM‐1 levels increased in BALF and serum during fungal asthma. MMP‐9 levels also increased during fungal asthma BALF IL10 and IL13 lower when TREM‐1 was blocked, both IL12 and CCL22 increased. TNFα, IL12, CCL2, CCL3, CCL5, CCL17 and CCL22 were elevated in whole lung tissue when TREM‐1 blocked Innate and adaptive immune responses mediated by TREM‐1 were critical for clearance of AF conidia from lungs Without cell‐associated TREM‐1, allergic mice exhibited an exacerbated form of lung disease
Causton et al., 2015^40^	*A. alternata*	AA‐induced expression of IL8, CCL20, GM‐CSF and TSLP were attenuated in NHBEs CARMA KO versus controls Predominant GPCRs in mouse AECs include P2Y_2_R, PAR1 and PAR2 CARMA3 mediates proinflammatory cytokine and chemokine production downstream of multiple GPCRs
Causton et al., 2018^41^	*A. alternata*	Reduced inflammation and eosinophil and neutrophil BALF counts in mice with reduced AEC CARMA3 expression Protein levels of IL33 24 h after single AA dose were lower in CARMA deficient mice versus controls. CARMA3 KO of NHBEs showed reduced IL33 RNA levels at 6 h and reduced protein levels AA‐exposed CARMA3 KO reduced expression of Th2 cytokines and ILC2 numbers and AA‐induced Ca^2+^ flux was delayed in CARMA3 KO ECs CARMA3 was associated with several other intracellular signalling cascades including ITPR3‐mediated Ca^2+^ flux CARMA3 promoted allergic airway inflammation in response to AA and was necessary for IL33 production and immediate release after allergen exposure
Chen et al., 2011^42^	*Penicillium citrinum* protease Pen c 13, normal and denatured	Pen c 13 exposed mice showed greater AHR, marked GCM, and increased BALF leukocytes and total IgE versus controls Pen c 13 exposed mice showed increased collagen deposition and elevated lung hydroxyproline versus controls Pen c c13 treatment resulted in time‐dependent cleavage of occludin and E‐cadherin. ZO‐1 was markedly reduced versus control and TEER decreased in a time‐dependent manner 20% differentially expressed proteins were related to oxidoreduction and 8% to protein folding Pen c 13 drove Th2 and IgE associated inflammation in the lung and may contribute to tissue damage associated with AAD such as asthma
Chiu et al., 2007^43^	*P. citrinum* protease Pen c 13	Pen c 13 caused dose‐dependent increase in IL8 secretion, mRNA level for IL1B, IL8, IL6 and GM‐CSF increased in cells. IL8 secretion was abolished by serine protease inhibitors and PAR1 and PAR2 antibodies ERK inhibitor and PLC inhibitor also reduced IL8 release Pen c 13 increased PAR1 and 2 mRNA and protein levels in cells and induced a rapid increase in Ca^2+^ via PAR1 and PAR2. ERK phosphorylation peaks within 10–15 min of exposure and then declined Pen c 13 had serine protease activity that activated PAR1 and PAR2, inducing IL8 release via PLC Pen c 13 induced IL8 expression by Ca^2+^‐dependent signalling and increased in Ca^2+^ is upstream of ERK half activation
Chung et al., 2007^44^	*Penicillium chrysogenum*, crude antigen preparation (PCE)	NGF levels were increased in BALF and serum of mice exposed to 50 and 70 μg PCE at D0 and D1 versus controls. Single exposure did not increase NGF NT4 levels increased in BALF and serum of mice exposed to 50 and 70 μg PCE versus controls. Single exposure did not increase NT4 NT3 levels increased in BALF of mice exposed to 50 and 70 μg PCE versus controls. Single exposure did not increase NT3 There was no change in BDNF levels between PCE‐treated mice versus control
Daines et al., 2020^45^	*A. alternata*, lyophilised cake of filtrate	Double dose of AA was required for response in ALI cultures versus monolayers AA induced dose‐dependent AHR and increased eosinophils and neutrophils in BALF versus control, responses not affected by PAR2 KO Cytokines upregulated in response to AA included: G‐CSF, IL1α, IL6, IL5, IL9 and IL17. PAR2 KO did not affect cytokine levels in BALF EGFR‐specific inhibitor repressed AA‐induced IL6 and IL8 in HBEs PAR2 did not appear to play a role in AA‐induced epithelial cytokine expression but was at least partially mediated by EGFR pathway
De Luca et al., 2017^46^	*A. fumigatus* viable resting conidia and culture filtrate	Increased GCM and peri‐bronchial collagen deposition as well as increased BALF eosinophils and total IgE in AF‐sensitised *IL17ra* ^*−/−*^ mice versus AF‐sensitised WT and *IL17f* ^*−/−*^ Silencing of *IL17rc* resulted in lower inflammatory pathology in all groups Expression of Th2 cytokines and Th17 cytokines were elevated in AF‐sensitised *IL17ra* ^*−/−*^ mice versus AF‐sensitised WT while IL10 was downregulated IL17F/IL17RC axis dysregulation caused a predisposition to allergic inflammation *IL17ra* ^*−/−*^ mice developed type 2 inflammation to AF and IL17RA was required for restraining allergic reactivity to exposure to AF
Doherty et al., 2012^47^	*A. alternata*, *Candida albicans, A. fumigatus* extracts	Only mice that received AA developed significant airway eosinophilia versus AF and CA. AA induced eosinophilia in a dose‐dependent manner. BALF IL33 and lung IL5 & IL13 increased after AA challenge versus control *Alternaria* specifically induced eosinophilia versus to other fungal allergens and induced FIZZ1, which persisted for days FIZZ1 and acute inflammatory events induced by AA were STAT6‐dependent but not PAR2‐dependent FIZZ1 had roles in promoting eosinophilia, epithelial changes and peribronchial fibrosis
Fritzsching et al., 2016^48^	*A. fumigatus*	Reduced MCC and AF clearance in *Scnn1b‐Tg* mice and increased airway eosinophilia, IL4, IL5, IL13, ILC2 numbers and AHR. *Scnn1b‐Tg* mice spontaneously increased IL5, IL13, GCM and AHR versus WT vehicle control. Response to AF exposure was age‐dependent Airway inflammation, GCM, IL13 and eosinophilia were reduced in STAT6 KO *Scnn1b‐tg* mice and were further protected after AF exposure IL33 induced IL13 not dependent on STAT6 but blocked by IL33/ST2 pathway inhibitors STAT6 signalling mediated the inflammatory response to AF Mucociliary dysfunction caused reduced clearance of AF
Gao et al., 2014^49^	*A. fumigatus* conidia	Airway resistance increased in groups C and D versus A, B, E, F, G with increasing levels for longer AF exposure Group A showed scattered airway epithelial injury and shedding as well as GCM, versus Group E, F, G. Group B, C, D progressively severe epithelial shedding and GCM TGF‐α and EGF in BALF increased in groups B, C, D versus A, E, F, G with increasing levels for longer AF exposure EGFR expression increased in groups B, C, D versus A, E, F, G with increasing levels for longer AF exposure Inhalation of AF in asthmatic rats aggravated epithelial injury Chronic exposure upregulated EGFR expression and its ligands and was involved in progressive increase in airway responsiveness
Giridhar et al., 2016^50^	*A. fumigatus* extract	AF exposure increased AHR and inflammatory responses in *Kif3a* null mice. Eosinophils increased in BALF and Th2 cytokines increased in mouse lungs GCM and mucus hyperproduction observed in mice of all genotypes after AF exposure but were more severe in mice lacking *Kif3a* and *Ift88* KIF3A had a role in microtubule assembly and in the pathogenesis of fungal induced inflammation and AHR. KIF3A was required for the suppression of Th2 mediated inflammatory responses, mucus hyperproduction and AHR
Gordon et al., 2011^51^	*A. fumigatus* antigen (culture extract)	Mucin production and peribronchial fibrosis was observed after AF challenge but there was no difference to WT controls Periostin deficient mice had increased AHR and serum IgE levels following allergen challenge versus WT Periostin deficient mice had blunted TGF‐β responses to allergen, which affected differentiation of Treg cells Periostin's role in the airway was to regulate AHR and allergen induced IgE
Haczku et al., 2006^52^	*A. fumigatus*, culture extract	Single AF exposure led to marked cellular influx of neutrophils and eosinophils and increased Th2 cytokines. Eosinophilia persisted over 48 h after challenge, whilst neutrophils returned to normal. BALF SPD protein levels markedly increased 48 h after AF challenge Sensitised mice that received rIL4 and rIL13 but not rIFNy increased SPD in BALF SPD production after AF challenge was absent in *Stat6* ^*−/−*^ versus WT controls SPD production increased in response to IL4 and IL13 and was STAT6‐dependent
Hayes et al., 2018^53^	*A. alternata*, allergen Alt a1	Alt a1 induced MCP‐1, IL8, CXCL1, CXCL2 and CXCL3 cells. IL8 was peaked at 24 h and was dose‐dependent Alt a1 primarily induced cytokine secretion (IL8, MCP‐1 and CXCL1/2/3) through TLR4 receptor activation. TLR2 receptor was also involved but to a lesser extent
Homma et al., 2016^54^	*A. fumigatus* extract, high molecular weight‐AF low molecular weight‐AF heat treated AF extract	AF extract supressed Poly I:C activated IRF‐3 but not NF‐κB. AF extract also triggered rapid dephosphorylation of IFNβ induced phospho‐STAT1 Suppressive effect of AF on induction of CXCL10 by TLR3 activators was mediated via activation of PAR2 and in part by PTPN11 activation PAR2 activation mobilised phosphatases that reduced TLR3 signalling through STAT and IRF
Hristova et al., 2016^55^	*A. alternata* extract	BALF: AA exposure resulted in increased ATP and IL33 in WT versus controls. IL33, IL5, IL13 and IL25 reduced in DUOX1 KO mice. AA induced IL33 reached max levels within 2 h but was not associated with cell necrosis IL33 release was not associated with increased mRNA but showed export of IL33 from nucleus to cytoplasm. IL33 release was dependent on P2Y_2_R and DUOX1 DUOX1 silencing mediated allergen induced H_2_O_2_ production in cells, decreased IL33 but did not affect ATP levels. DUOX1 and IL33 was dependent on Ca^2+^ AA exposure induced rapid phosphorylation of Src, EGFR and ERK1/2 and enhanced EGFR phosphorylation. Response absent in DUOX1 KOs DUOX1 had a specific role in Th2 immune response and was markedly elevated in asthmatic subjects, contributing to exaggerated airway response. IL33 release was mediated by DUOX1 activation of the EGFR pathway. Duox1 was also upregulated in asthmatics
Iijima et al., 2021^56^	*A. alternata* extract	CCSP‐Il33tg: IL33 overexpression upregulated IL5 expression in BALF but not IL13 at baseline IL25, TSLP and IL13 cytokines were comparable in transgenic mice and controls exposed to AA IL33 upregulation increased IL5 production and secretion Transient increased IL33 in the neonatal period enhanced fungal induced airway innate type 2 immune responses later in life
Inoue et al., 2021^57^	*A. alternata* extract	AA exposure caused prominent airway inflammation, increased eosinophils, membrane thickening, increased mucus production and increased airway remodelling RNA‐seq: 403 upregulated genes and 108 downregulated genes after 6 weeks exposure ST2 expression was increased in AA exposure, IL33 was marginally decreased versus controls AA downregulated keratinisation in epithelial cells and upregulated genes relating to Ig expression and receptors as well as serine proteases such as Tpsb2 and Tmprss2 and eosinophil migration
Jeong et al., 2018^58^	*A. fumigatus* crude antigen extract, *A. alternata* crude antigen extract	NLRP3 was significantly increased in patients with ABPA and to a lesser extent, IPF. NLRP3, caspase 1 and ASC increased and co‐localised in AF‐exposed mice and in AF‐exposed epithelial cells, which was reduced by PI3K‐δ inhibitors IL1β inhibition improved AF induced allergic lung inflammation, including reductions in infiltrating cells AA‐induced allergic inflammation: NLRP3 and PI3K‐δ inhibitors reduced increases in eosinophils in BALF, Th2 cytokines (IL4, IL5, IL13) and IL1β in lungs of mice NLRP3 inflammasome assembly and activation was increased in the lungs of AF‐ and AA‐exposed mice PI3K‐δ played a role in NLRP3 inflammasome assembly and activation in bronchial epithelial cells
Kato et al., 2017^59^	*A. alternata* crude allergen extract	Knock‐down of RAGE and TLR4 receptors as well as calprotectin proteins significantly inhibited IL25 and TSLP Calprotectin was involved in AA‐induced TSLP and IL25 production as well as the ATP pathway and played an important role in Th2 airway inflammation Airborne allergens stimulated production and release of calprotectin and protease activity was crucial in this process Allergen induced calprotectin was increased in cells from CRS patients
Khosravi et al., 2018^60^	*A. fumigatus*, conidia and heat‐inactivated conidia (HI‐AF)	A concentration‐dependent increase in TLR2 and TLR4 expression was observed upon exposure to HI‐AF. TLR2 was upregulated by both live AF and HI‐AF. TLR4 was upregulated by HI‐AF but downregulated by live AF AF spores stimulated increased secretion of Th2 cytokines IL25, IL33 and TSLP. IL25 and IL33 induced by live AF was higher than that of HI‐AF. IL25 induction was higher than other cytokines
Kim et al., 2018^61^	*Aspergillus oryzae*, protease solution (active and inactive)	JNK and ERK phosphorylation and AP‐1 expression increased in cells treated with AO versus controls. ROS production and mtROS increased in a concentration‐dependent manner Reducing ROS resulted in reduced ERK phosphorylation, decreased AP‐1 and reduced cytokine secretion AO proteases regulated expression of inflammatory cytokines and induced mtROS, activating MAPK and AP‐1 mtROS production inhibited UPC2 protein expression via TGF‐β and SMAD4
Kouzaki et al., 2009^62^	*A. alternata* culture filtrate extract	Release of TSLP in ECs involved PAR2 and serine proteases TSLP increased in a concentration‐dependent manner in response to AA exposure IFNγ inhibited TSLP release from AA‐exposed ECs
Kouzaki et al., 2011^63^	*A. alternata* culture filtrate extract	IL5 and IL13 increased in lungs of AA exposed mice versus control but decreased within 24 h. After 1 h, IL33 increased markedly in BALF, preceding IL5 and IL13 increases P2 purinergic antagonists and KO suppressed AA induced Ca^2+^ response ECs actively and rapidly released IL33 in response to fungal allergens Ca^2+^ and ATP was involved in the translocation of IL33 from the nucleus to the cytoplasm and IL33 release was dependent on P2Y_2_Rs
Labram et al., 2019^64^	*A. fumigatus*, spores and culture filtrates	Spore inhalation resulted in mild increase in IL4, IL5 and IL6, whereas culture filtrate induced a larger increase Treatment with ET‐1 antagonist reduced extent of peribronchiolar inflammatory infiltration, reduced total BALF cell count and reduced collagen deposition. ET‐1 antagonist treatment also reduced AF‐induced IL4 and IL6 ET‐1 was upregulated in response to aspergillus exposure, but protein levels remain unchanged in the lung. Culture filtrate resulted in enhanced endothelin protein in BALF Culture filtrate exposure showed increased airway remodelling in comparison to spore exposure
Lee et al., 2020^65^	*A. fumigatus* crude antigen extract	AF exposure: Infiltration of eosinophils into bronchioles versus control group. Increased AHR, Th2 cytokines (IL4, IL5, IL13) and PAS staining. ER membrane showed aberrant morphology. Distance between ER and mitochondria was smaller than usual, all reduced by PI3K‐δ inhibitors AF exposure induced increase in protein oxidation, decreased GSH:GSSG ratio, increased HMWC formation and enhanced unfolded protein response. Inhibitors reduced AF‐induced changes Exposure to AF increased mitochondrial ROS and decreased ATP levels while inhibiting COX1 and COX3 activities PI3K‐δ and ER stress contributed to airway inflammation and remodelling in exposure to AF. ER stress marker GRP78 increased by AF exposure and reduced by PI3K‐δ inhibitors AF caused changes in ER membrane fluidity and permeability, close contact between ER and mitochondria, and amplified ROS and Ca^2+^ signalling AF exposure induced inflammasome activation via VDAC1 and caspase1. VDAC silencing reduced ASC, activated caspase 1 and NLRP3
Lee et al., 2016^66^	*A. fumigatus* crude antigen extract	AF exposure: Increased BALF cell numbers (especially eosinophils), IgE, Th2 cytokines and AHR. Th2 cytokines were reduced by NF‐κB inhibitors ER stress was increased in lungs of AF‐exposed mice GRP78 was also upregulated in lungs of ABPA patients, and GRP78 and CHOP were upregulated in AF‐exposed mice ER stress may be involved in pathogenesis of AF‐related allergic lung disorders, ER stress involved PI3K‐δ and mtROS generation
Leino et al., 2013^67^	*A. alternata, Cladosporium herbarum*, culture extracts	Significant dose‐dependent decrease in TEER in polarised cultures observed 1 h post challenge with AA but recovered quickly at lower doses. Higher dose of AA, TEER remained lower at 24 h. CH did not affect TEER. In ALI cultures, AA had no effect on barrier function in healthy donors but severe asthmatics saw a dose‐dependent decrease within 3 h but recovered by 24 h AA significantly induced release of inflammatory cytokines and increased permeability of polarised epithelial cells potentially due to serine and aspartate protease activity Differentiated primary cells had a blunted IL8 response but those from asthmatics were more susceptible to barrier weakening
Matsuwaki et al., 2012^68^	*A. alternata, Aspergillus versicolour, A. fumigatus, C. albicans, C. herbarum*, *Penicillium* spp., *Curvularia* spp., culture filtrate extracts	Cells stimulated with AA showed rapid increase in cytosolic free Ca^2+^, observed after 200 s and peaked between 400 and 600 s Only AA induced IL6, IL8 and GM‐CSF production versus other fungi. AA did not induce eotaxin, eotaxin‐2 or RANTES. HI‐AA did not induce cytokine production Aspartate protease inhibitors inhibited cytokine production and Ca^2+^ response in AA exposed epithelial cells Aspartate proteases but not serine proteases in AA activated PAR2 in epithelial cells AA but not other fungal extracts activated IL6 production/release
Munitz et al., 2011^69^	*A. fumigatus,* culture extract	AF induced GCM and mucus production as well as production of CCL17 and CCL22 AF induced upregulation in RELM‐α expression but *Il13ra1* ^*−/−*^mice showed no increase IL13Rα1 critically regulated RELM‐α expression after AF challenge Baseline RELM‐α expression was restricted to airway cells. AF challenge increased expression in macrophages RELM‐α did not have a marked role in the production of Th2 cytokines after allergen challenge
Murai et al., 2012^70^	*A. alternata, Aspergillus, Penicillium* extract (Asp and Pen species unknown)	AA increased cell permeability and induced necrosis AA rapidly induced increases in IL18 levels in cells (26‐fold) and BALF (8.6‐fold). AA induced much greater IL18 release versus *Aspergillus* and *Penicillium.* IL18 induced Th2 differentiation, which was dependent on NF‐κB AA induced specific IL18 release from AECs versus to other allergens. IL18 release occurred through activation of caspase 1 and induced necrosis
Murai et al., 2015^71^	*A. alternata* extract	AA treatment increased IL18 levels in culture supernatants. Caspase 1 and caspase 8 inhibitors failed to inhibit IL18 release. PI3K inhibitors suppressed AA‐induced IL18 AA treatment augmented formation of autophagosome AA activated an autophagy‐based unconventional secretion pathway in airway epithelial cells and induced the extracellular release of IL18 independent of caspase I and 8 activation
Neveu et al., 2009^72^	*A. fumigatus* extract	Goblet cell numbers were reduced in *Il6* ^*−/−*^ mice and MUC5AC expression was reduced upon AF exposure versus controls IL6 directly induced IL13 production on CD4^+^ T cells and was essential for mucus production IL6 and IL21 deficiency resulted in IgE hypersecretion and increased IgE during allergic inflammation. IL6 contributed to IgG1 production IL6 deficiency impaired IL17 production in induced allergic inflammation IL6 was rapidly produced in the lung upon allergen exposure and played a key role in dictating immune response against allergens
Neveu et al., 2011^73^	*A. fumigatus, Candida albicans* extract	TSLP, IL33, TNFα or eotaxin were not detected in supernatants of LECs in vitro after direct contact with AF allergens β‐glucans predominantly stimulated IL6 production from LECs Direct interaction between LECs and allergens produced IL6. IL6 gene was constitutively expressed in LECs but not in lung resident immune cells p38 MAPK was involved in IL6 production by promoting phosphorylation of Mnk1/2 kinases
O'Grady et al., 2013^74^	*A. alternata* extract and antigen preparation	Cytokeratin 14, 16 and 6RA expression was higher in primary HBE & HBE‐A cells versus 16HBE, however, 16HBE had higher mucin expression Inhibitors reduced AA induced ATP release by reducing intracellular vesicle movement of ATP Serine and cysteine protease inhibitors reduced max levels of ATP in HBE cells, but only Leupeptin reduced ATP levels in HBE‐A cells Inhibition of PAR2 receptors did not block effects of AA treatment on Ca^2+^ signalling HBE‐A cells were more sensitive to AA and produced quicker and higher ATP and Ca^2+^ release versus normal cells ATP release was via PAR2 receptor, but was not fully dependent on it
Oguma et al., 2011^75^	*A. fumigatus* (fungal extract and culture supernatant), *Aspergillus niger*, *Penicillium notatum*, *A. alternata*, *C. albicans* fungal extracts	AF induced prominent mucus production versus other fungi and control. Weaker mucus production by PN extract versus CA, AA and control One hundred and eighty five genes specifically upregulated by AF but not PN or AA EGFR and TGF‐α inhibitor markedly reduced MUC5AC expression in AF‐exposed cells. MMP inhibitor or TACE inhibitor lowered level of MUC5AC mRNA versus control. MUC5AC mRNA was also suppressed by TACE siRNA versus control The robust serine protease activity of AF was essential for mucin synthesis and expression of MUC5AC in AECs via activation of TACE/TGF‐α/EGFR pathway
Patel et al., 2019^76^	*A. fumigatus* fungal extract *A. alternata* fungal extract	Frequency of SCCs among epithelial cells was found to be higher in inflamed mucosa versus non‐inflamed turbinate tissue for all groups Frequency of SCCs in AFRS primary cells were elevated in AF and AA versus control SCCs played a role in detection of fungal antigens causing release of IL25 SCCs were upregulated in inflamed mucosa in non‐invasive allergic fungal conditions when exposed to fungal antigens
Ramu et al., 2017^77^	*A. alternata* allergen extracts	AA caused dose‐dependent release of IL8 and ATP AA showed serine protease activity and IL8 mRNA expression and ATP release was inhibited by serine protease inhibitors but IL8 protein was not Allergens show distinct differences in their ability to induce DAMPs and alarmins in cells
Rivas et al., 2021^78^	*A. alternata* extract	Impedance assay: AA (at lowest dose) caused initial decrease of cell index followed by an increase similar to protease and PAR2 agonist response. Higher dose led to a secondary loss of cell index in normal cells AA induced IL6 and IL8 secretion as well as RANTES, VEGF, PDGF and IP10 at 24 h, which was reduced by PAR2 antagonist. PAR2 KO cells had reduced cytokine secretion HBSS control had increased IL8, IP10, RANTES, and TNFα from apical side, suggesting a PAR2‐independent pathway exists AA acted on the PAR2 receptor, but also induced a PAR2‐independent pathway
Royce et al., 2011^79^	*A. fumigatus* antigens	AF‐exposed *Tff2* ^*−/−*^ mice showed marked increase in epithelial thickness and goblet cell numbers versus WT controls TFF2 attenuated changes in epithelial structure, GCM and subepithelial collagen deposition TFF2 maintained normal homoeostatic structure of airway
Samichuwal et al., 2017^80^	*A. alternata* cellular extract	*mPGES* KO mice showed decreased lung inflammation and reduced ILC2s versus WT *mPGES* KO expressed lower levels of IL33 mRNA versus WT PGE2 supported the amplification of IL33 expression, which in turn drove downstream IL33‐dependent effectors
Schiffers et al., 2020^81^	*A. alternata* culture extract	mRNA expression of P2Y_2_R and TRPV1 was enhanced in asthmatic NECs versus healthy controls. PAR1 and PAR2 were also increased AA induced IL33 release suppressed by TRPV1 but not TRPV4. P2Y_2_R and TRPV1 silencing reduced IL33 release AA exposure produced extracellular H_2_O_2_ (DUOX1 activation), which was supressed by P2Y_2_R inhibitor PAR2 and TRPV1 activation evoked rapid ATP release. Inhibition of TRPV1 strongly attenuated activation of EGFR TRPV1/4 induced IL33 secretion requires P2Y_2_R‐dependent DUOX1 activation AA induced DUOX1 activation, EGFR activation and IL33 secretion involve TRPV1. TRPV1 critically contributed to ATP‐dependent signalling with P2Y_2_R
Schmit et al., 2020^82^	*A. fumigatus* spores and culture extract	AF exposure caused broncho‐interstitial infiltration of immune cells and GCM with higher mucus production and higher levels of eosinophils IL6 deficiency correlated with decrease goblet cell numbers and mucin production, and decreased MUC5AC expression IL6 deficiency drove increased eosinophil recruitment, lung pathology and collagen deposition in asthmatic mice IL6 deficient eosinophils dysregulated lung inflammation by upregulating TGF‐β and STAT3 phosphorylation, compromising barrier integrity
Shibata et al., 2014^83^	*A. fumigatus* antigens and conidia	Gas6^−/−^ mice had suppressed AHR, decreased peribronchial and BALF inflammatory cells, reduced PAS staining and serum IgE Exogenous Gas6 treatment to AF exposed mice led to increased peribronchial inflammation, increased BALF cells, increased serum IgE, and increased PAS staining Gas6 was elevated in asthmatic populations regardless of disease severity Gas6 drove airway inflammation and was involved in airway remodelling
Shin et al., 2019^84^	*A. alternata* culture extract	TEER decreased in a concentration‐dependent manner when exposed to AA but returned to baseline by 24 h, except at highest dose AA strongly induced intracellular ROS in cells, but effect was reduced with serine protease inhibitor and heat‐inactivation AA affected TJ molecule but not AJ molecule expression AA‐induced barrier dysfunctions were associated with serine proteases and intracellular ROS
Shin et al., 2020^85^	*A. alternata, A. fumigatus* culture filtrate	ENP patients had olfactory dysfunction and tissue eosinophilia versus NENP patients. IL25, IL33 and TSLP levels in ENP patients were higher versus NENP and controls AA stimulated IL33 and TSLP production but not IL25 for 24 and 48 h. IL25 was not increased by allergens AA enhanced IL33 and TSLP and IL6 but AF only increased IL6 production. AA enhanced phosphorylated NF‐κB and c‐Jun expression, but AF did not. IL33 and TSLP expression was regulated by NF‐κB, AP‐1 and MAPK
Srisomboon et al., 2020^86^	*A. alternata* extract	AA‐induced IL33 release decreased by inhibition of VDAC1 in a concentration‐dependent manner Inhibitors and silencing of VDAC1 reduced AA‐induced release of ATP and Ca^2+^. VDAC1 was involved in ATP release in epithelial cells exposed to AA Reduced cholesterol inhibited initial ATP release. Cholesterol played a role in ATP release and Ca^2+^ uptake in AECs exposed to AA Plasma membrane localisation of VDAC1 was dependent on cholesterol
Tai et al., 2006^87^	*P. chrysogenum*, protease Pen ch 13	Pen ch 13 induced production of PGE2, IL8 and TGF‐β in AECs Mediator release and COX‐2 were inhibited by a serine protease inhibitor, Pen ch 13 degraded occludin, which can disrupt the epithelial barrier
Uchida et al., 2017^88^	*A. alternata, A. fumigatus* extracts	AA‐exposed mice treated with CDDO‐Me had reduced airway eosinophilia, IgE expression, peribronchial inflammation and GCM. IL5 and IL13 expression were also reduced. CDDO‐Me reduced or abolished IL33 release after AA exposure Full length IL33 remained in tissues, short IL33 was secreted IL33 release was associated with increased ROS, ATP release and Ca^2+^ signalling Activation of Nrf2 pathway reduced allergic pathology, type 2 cytokines and IL33 release
Wiesner et al., 2020^89^	*A. fumigatus* Alp1 (Asp f13) and culture filtrate	Alp1 caused allergic pathology in lung including leucocyte infiltration and GCM TSLP did not increase from baseline after Alp1 exposure but IL25 and IL33 increased post 24h Alp1 did not cleave PAR1 or 2. Alp1 cleaves C3 to form C3a like molecule but does not affect allergic response Elevated TRPV4 expression in asthmatics exacerbated the inflammatory response to proteases Epithelial damage through barrier integrity sensed via TRPV4 prompting Ca^2+^ flux‐ and calcineurin‐dependent inflammation
Wong et al., 2020^90^	*A. fumigatus* crude extract	RGS4 immunoreactivity was more extensive in asthmatic versus normal bronchial epithelium and increased proportionally with disease severity MUC5AC expression correlated with RGS4 expression PGE2 was detected at higher levels in BALF from AF‐exposed mice versus controls and levels were even higher when RSG4 was knocked down. AF challenge caused increased PGE expression through PAR2 activation PAR2 mediated bronchodilation through an RGS4‐regulated pathway in mice. RGS4 inhibited PGE2 secretion by limiting GPCR‐induced G protein activation upstream of PGE2 biosynthesis
Wu et al., 2020^91^	*A. fumigatus* extract	AF dose‐dependently induced MUC5AC expression. Increase occurred as early as 6 h after treatment. MUC5B also dose‐dependently increased but less than MUC5AC AF‐induced mucin expression repressed by EGFR tyrosine kinase inhibitors, EGFR blocking antibody, and serine protease inhibitors. PAR2 neutralising antibody did not block mucin gene expression AF‐induced expression of MUC5AC and MUC5B in ECs independent of PAR2. EGFR activity was required, but not sufficient, to induce mucins. Mucins induced by activation of ERK pathway. Blocking Ca^2+^ but not ROS prevented mucin induction
Zaidman et al., 2017^92^	*A. alternata* culture extract	Epithelial barrier integrity remained intact despite reduced TEER values with AA exposure AA exposure stimulated transepithelial anion secretion through the CTFR and CaCC channels. AA‐induced secretion was blocked by channel inhibitors. ATP release from epithelium activated Ca^2+^ uptake through CaCC mediated pathway in apical membrane Increase in Ca^2+^ stimulated Cl^−^ efflux AA exposure stimulated ATP secretion as early as 5 min and increased up to 20 min Increase in oxidative stress contributed to decrease in epithelial resistance

Abbreviations: AA, *Alternaria alternata*; AAD, allergic airways disease; ABPA, allergic bronchopulmonary aspergillosis; AEC, airway epithelial cells; AF, *Aspergillus fumigatus*; AFRS, Allergic fungal rhinosinusitis; AHR, airway hyperresponsiveness; AJ, adherens junctions; ALI, air‐liquid interface; AO, *Aspergillus oryzae*; AP‐1, activator protein 1; ASC, apoptosis‐associated speck‐like protein containing a CARD; ATP, adenosine triphosphate; BALF, bronchoalveolar lavage fluid; BDNF, brain‐derived neurotrophic factor; CA, *Candida albicans*; Ca^2+^, calcium; CaCC, calcium‐dependent chloride channel; cAMP, cyclic adenosine monophosphate; CARMA, CARD‐containing membrane associated guanylate kinase protein; Cav‐1, caveolin‐1; CCL, chemokine (C‐C motif) ligand; CCSP, club cell secretory protein; CDDO‐Me, bardoxolone methyl; CFTR, cystic fibrosis transmembrane conductance regulator; CH, *Cladosporium herbarum*; CHOP, C/EBP homologous protein; Cl^−^, chloride; COX, cytochrome c oxidase; cPLA2γ, cytosolic phospholipase Α2γ; CRS, chronic rhinosinusitis; CXCL, chemokine (C‐X‐C motif) ligand; CysLTR, cysteinyl leukotriene receptor; DAMP, danger‐associated molecular pattern; DAP12, DNAX‐activation protein of 12 kDa; DUOX, dual oxidase; EC, epithelial cells; EGF, epidermal growth factor; EGFR, epidermal growth factor receptor; ENaC, epithelial sodium channel; ENP, eosinophilic nasal polyps; ER, endoplasmic reticulum; ERK, extracellular signal‐regulated kinase; ET, endothelin; FIZZ1, found inflammatory zone 1; Gas, growth arrest—specific; GCM, goblet cell metaplasia; G‐CSF, granulocyte colony‐stimulating factor; GM‐CSF, granulocyte‐macrophage colony‐stimulating factor; GPCR, G‐protein coupled receptor; GRP, glucose regulated protein; GSH, glutathione; GSSG, glutathione disulphide; HBE, human bronchial epithelial cells; HBE‐A, human bronchial epithelial cells—asthmatic; HBSS, hanks balanced salt solution; HI, heat‐inactivated; HMWC, high molecular weight compound; IFN, interferon; Ig, Immunoglobulin; IL, interleukin; IP10, interferon gamma‐induced protein 10; IPF, idiopathic pulmonary fibrosis; IRF, interferon regulatory factors; ITPR, inositol 1,4,5‐trisphosphate receptors; JNK, c‐jun N‐terminal kinase; KIF3A, kinesin‐like protein 3A; KO, knock‐out; LECs, lung epithelial cells; LTC_4_, leukotriene C; LTE4, leukotriene E4; MAPK, mitogen‐activated protein kinases; MCC, mucociliary clearance; MCP‐1, monocyte Chemoattractant Protein‐1; MIP, macrophage inflammatory protein; MMP, matrix metalloprotease; Mnk, MAPK interacting protein kinases; mt, mitochondrial; NEC, nasal epithelial cells; NENP, non‐eosinophilic nasal polyps; NF‐κB, nuclear factor kappa‐light‐chain‐enhancer of activated B cells; NGF, nerve growth factor; NHBE, normal human bronchial epithelial; NLRP, nucleotide‐binding oligomerization domain, leucine rich repeat and pyrin domain containing; Nrf2, nuclear factor erythroid 2–related factor 2; NT, neurotrophin; P13, *Penicillium chrysogenum* protease 13; P2YR2, P2 purinergic receptor 2; PAR2, protease activated receptor 2; PAS, periodic acid schiff; Pc13, *Penicillium citrinum* protease 13; PCE, *Penicillium chrysogenum* crude antigen preparation; PDGF, platelet‐derived growth factor; PGE, prostaglandin E; PI3K, phosphoinositide 3‐kinase; PLC, phospholipase C; PN, *Penicillium notatum*; PTPN, tyrosine‐protein phosphatase non‐receptor; RAGE, receptor for advanced glycation endproducts; RANTES, regulated on activation, normal T expressed and secreted; RELM, resistin‐like molecules; RGS4, regulator of G protein signalling 4; ROS, reactive oxygen species; SCC, solitary chemosensory cells; SMAD, small mothers against decapentaplegic; SPD, surfactant protein D; Src, proto‐oncogene tyrosine‐protein kinase Src; ST2, suppression of tumorigenicity 2; STAT, signal transducer and activator of transcription; TACE, tumour necrosis factor‐alpha converting enzyme; TEER, trans epithelial electrical resistance; TFF, trefoil factor; TGF, transforming growth factor; Th, T helper; TJ, tight junctions; TLR, toll like receptor; Tmprss, transmembrane protease serine; TNF, tumour necrosis factor; Tpsb2, tryptase beta‐2; TREM, triggering receptor expressed on myeloid cells; TRPV, transient receptor potential vanilloid; TSLP, thymic stromal lymphopoietin; UCP, uncoupling protein; VDAC, voltage‐dependent anion channel; VEGF, vascular endothelial growth factors; WT, wild‐type; ZO, zonal occludens.

### 3.2 Models

**FIGURE 2:**
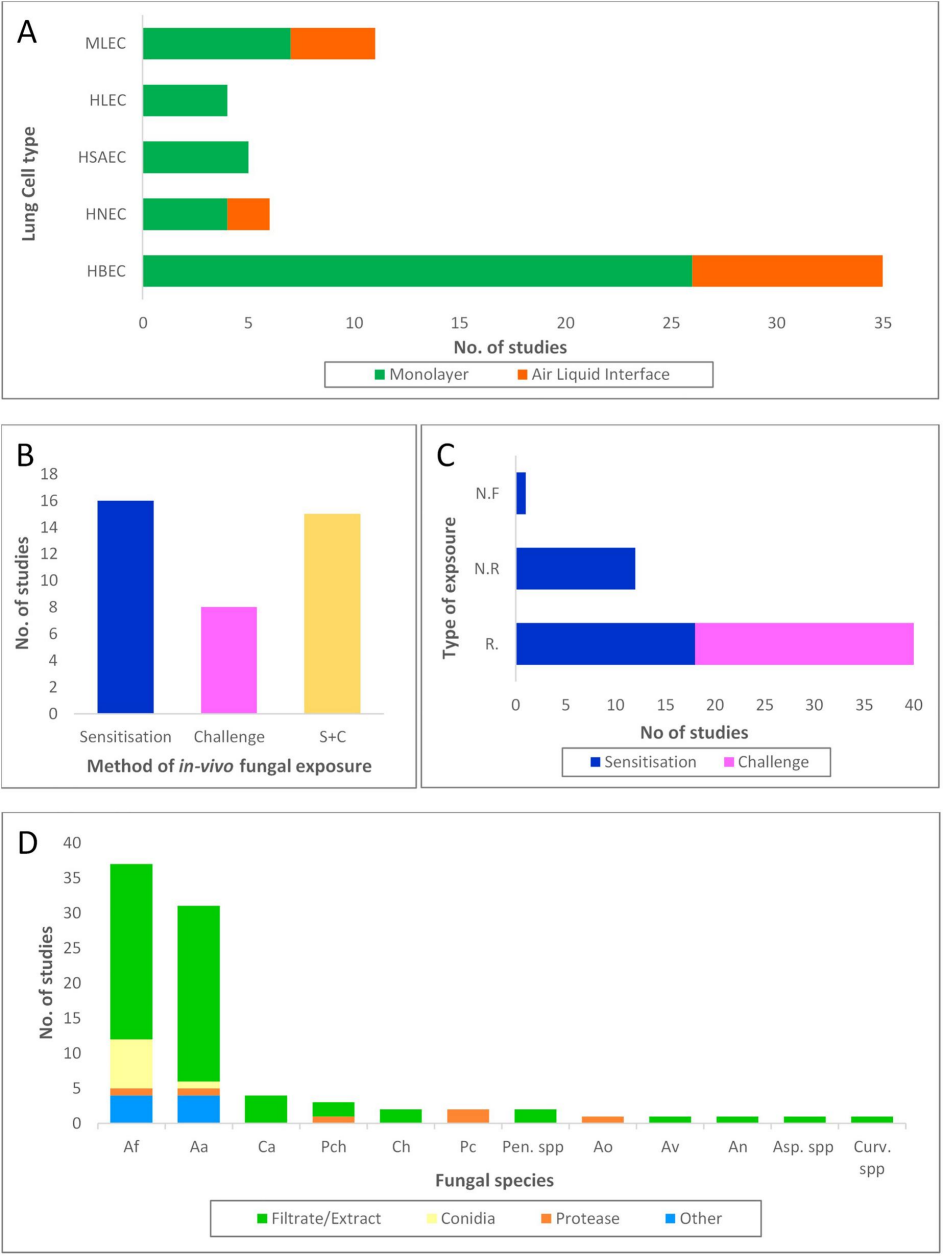
(A) Cell type and culture conditions used in in vitro studies^†^. HBEC, human bronchial epithelial cell; HLEC, human lung epithelial cell; HNEC, human nasal epithelial cell; HSAEC, human small airways epithelial cell; MLEC, mouse lung epithelial cells. (B) The method of fungal exposure used to induce allergic fungal disease in in vivo models. Sensitisation refers to prolonged exposure of several days or weeks and includes ‘chronic’ models^‡^. The challenge refers to a single exposure to a fungal allergen and includes ‘acute’ models^‡^. S + C (sensitisation and challenge) refers to sensitisation with prolonged exposure over a period of time and then a subsequent challenge often with a different method of exposure. (C) The type of exposure used when inducing allergic fungal disease in in vivo models through sensitisation and challenge methods. Respiratory methods (R) include aerosol, inhalational, intranasal, intratracheal and oropharyngeal applications of fungal allergens. Non‐respiratory methods (N.R.) include intraperitoneal and subcutaneous applications of fungal allergens. Where non‐fungal allergens have used this has been given as a separate category (N.F.). (D) Type and species^§^ of fungal allergen applied to models in selected studies. Aa, *Alternaria alternata*; Af, *Aspergillus fumigatus*; An, *Aspergillus Niger*; Ao, *Aspergillus oryzae*; Asp spp., *Aspergillus* species; Ca, *Candida albicans*; Ch, *Cladosporium herbarum*; Culv spp., *Curvularia* species; Pc, *Penicillium citrinum*; Pch, *Penicillium chrysogenum*; Pen spp., *Penicillium* species. ^†^Studies using lung epithelial cells did not describe the region from which lung epithelial cells were derived. ^‡^Studies which referred to prolonged exposure as ‘challenge’ have been recorded as sensitisation and studies which referred to a single exposure as ‘sensitisation’ have been recorded as a challenge. ^§^Studies where only the fungal genus has been given have been recorded as fungal spp (e.g. Aspergillus spp.).

Of the 61 studies, 24 (38%) were performed *in vitro*, 16 (27%) in vivo and 21 (35%) used both in vivo and in vitro models. An overview of in vitro models (*n* = 45) is described in Figure 2A. In vitro cell models included monolayers or differentiated air‐liquid interface (ALI) cultures, with 11 studies (15%) using primary human epithelial cells cultured at ALI for different periods of time. Of the 45 studies which included in vitro experiments, 26 studies used primary human bronchial epithelial (HBE) or human nasal epithelial (HNE) cells, whilst 25 studies used human cell lines or mouse cells (some studies used a combination of cell lines and primary cells). Of the 26 studies using primary cells, 6 (23%) included diseased primary cells (i.e., donors with asthma, allergic rhinitis or nasal polyps). In addition, only four of the in vitro studies (15%) directly compared healthy donor cells with asthmatic or allergic rhinitis cells.^53^, ^67^, ^74^, ^81^ Only 25% (*n* = 15) of the total studies used clinical tissues or diseased primary cells to identify differences in patients with allergic respiratory diseases versus controls.

*In vivo* studies (*n* = 37) predominantly used mouse models, with just a single study^49^ using a rat model. The induction of allergy methods in in vivo models are described in Figure 2B,C. Whilst all in vivo models used allergen exposure compared to a control to model AAD in some way, there was large heterogeneity in both the methods of sensitisation and challenge applied and the disease‐related outcomes investigated.

A range of different fungal species were used in the selected studies with *Aspergillus* (*n* = 41) and *Alternaria* (*n* = 31) being the most studied fungal genera. Fungal allergens were applied in various forms, including culture filtrate or extract (CFE), conidia and purified proteases. The breakdown of fungal allergens can be found in Figure 2D. Only one study compared conidia and CFE directly,^64^ and just 15% of studies (*n* = 9) compared more than one species of fungi, although *Alternaria* and *Aspergillus* were both included in seven of these studies. Nearly a third of studies (*n* = 19) identified the protease activity as being involved in the allergic response to fungi. However, only six studies focused exclusively on fungal proteases as their exposure compound.^42^, ^43^, ^53^, ^61^, ^87^, ^89^

### 3.3 Potential mechanisms of fungal‐induced allergy

**FIGURE 3:**
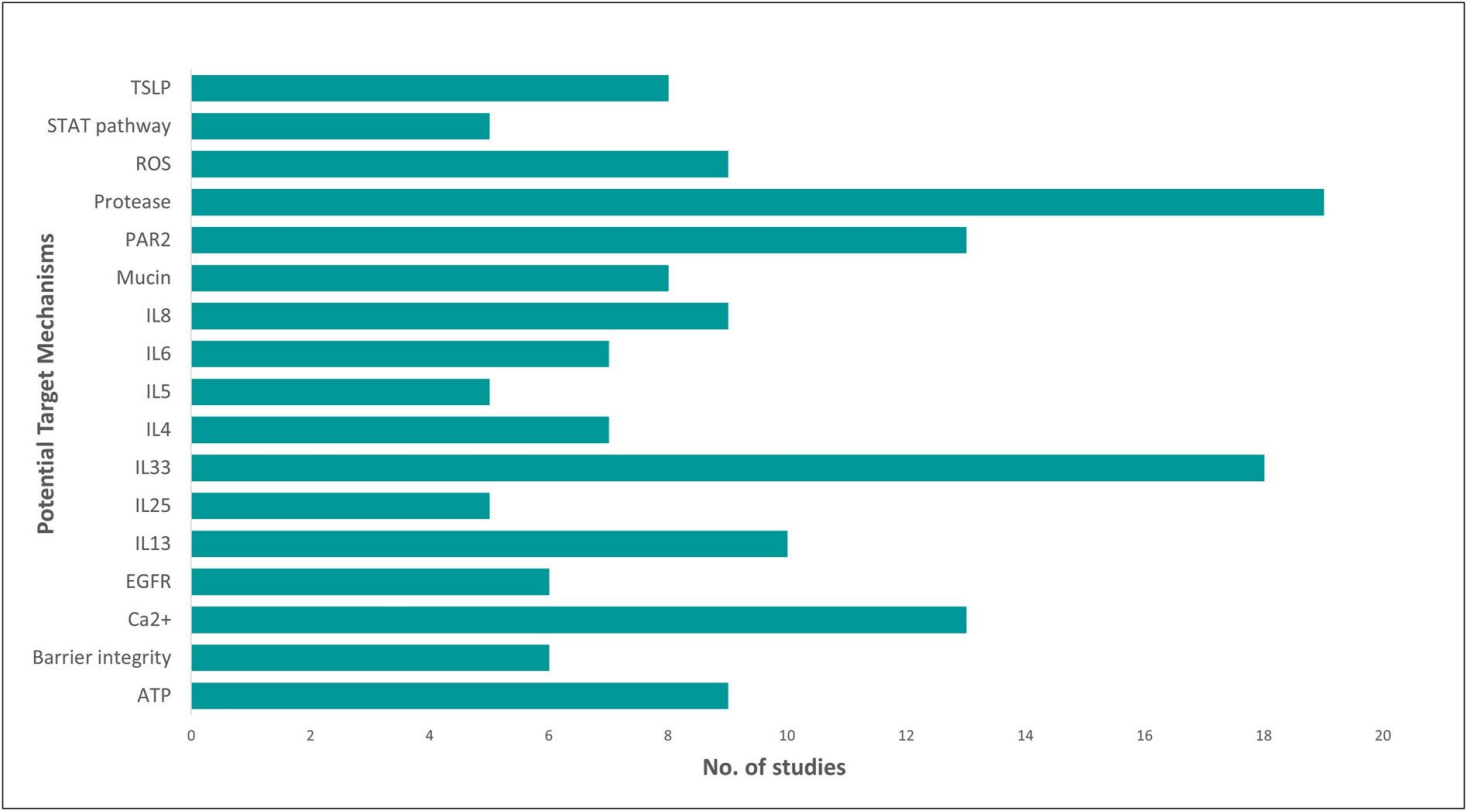
Potential target mechanisms identified in five or more studies. Potential target mechanisms were defined as any molecule or pathway that was either (a) the specific focus of the study, (b) a molecule or pathway that was further validated using knockouts, inhibitors or gene silencing/overexpressing techniques, or (c) an endpoint molecule where pathways or genes were manipulated to observe the effects on said molecule. Molecules that were investigated using appropriate validation techniques but found not to be involved in specific pathways were still included as potential target mechanisms. Molecules that were used as endpoints to confirm allergic responses in models were not included as potential target mechanisms unless they underwent further investigation. ATP, adenosine triphosphate; Ca^2+^, calcium; EGFR, epidermal growth factor receptor; IL, interleukin; PAR, protease‐activated receptor; ROS, reactive oxygen species; STAT, signal transducer and activator of transcription; TSLP, thymic stromal lymphopoietin.

In total, 120 potential target mechanisms (individual molecules or pathways) were identified, 65 (54%) were identified in only one study (Supporting Information S4). Of the 54 (44%) targets identified in more than one study, 17 (28%) of these were identified in five or more studies (Figure 3), giving a greater confidence that these potential target mechanisms are involved in the allergic response to fungi. Proteases were the most investigated target (*n* = 19, 31%), followed by IL33 (*n* = 18, 30%), calcium signalling, and protease‐activated receptor 2 (PAR2) (*n* = 13, 21% each).

### 3.4 Quality assessment

**FIGURE 4:**
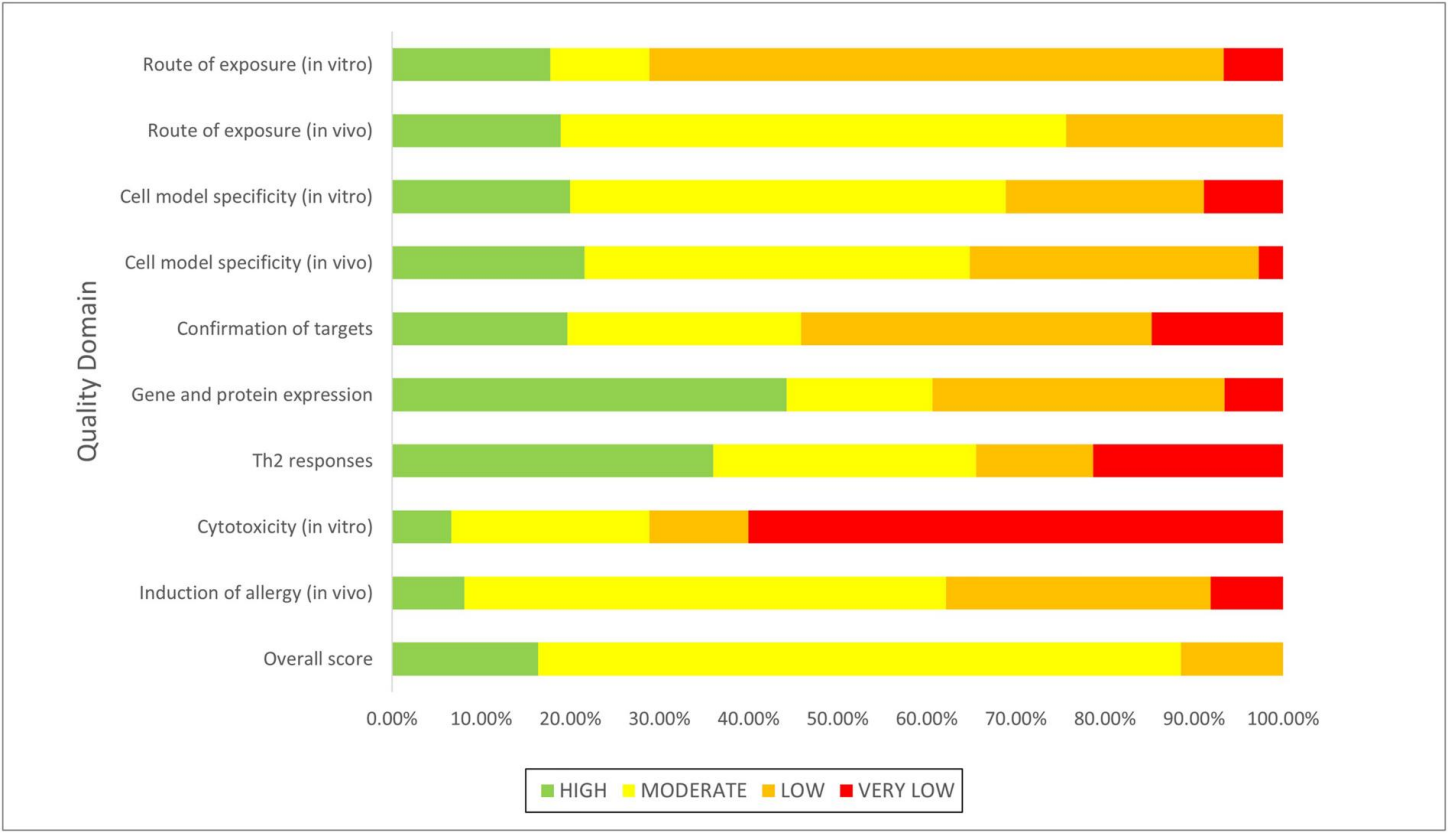
Overall composition of quality scores assigned to studies based on seven quality scoring domains. A detailed explanation of the quality scoring tool is available in Supporting Information S2, and the individual scores for each study are shown in Supporting Information S3. The seven domains were: route of exposure (physiologically relevant applications of fungal exposures), cell model specificity (physiologically relevant models, tissues or cells of the airways), confirmation of targets (using inhibitors/agonists), gene and protein expression (confirming gene expression changes with protein expression assays), Th2 responses (measuring specific Th2 cytokines), cytotoxicity (measuring cytotoxicity after application of fungal allergens [in vitro only]), induction of allergy (physiologically relevant methods of fungal sensitisation and challenge [in vivo only]).

An overview of how studies scored in each domain is shown in Figure 4. The overall scores of most studies were moderate (*n* = 44, 72%) or high (*n* = 10, 16%), with the remaining seven studies scoring as low (12%). The average total score was 2.6 (moderate), with the highest scoring domain being gene and protein expression (*n* = 27, 44% high), followed by Th2 responses (*n* = 22, 36% high). While 15 studies (25%) scored high for their in vitro or in vivo model(s) and 14 studies (23%) scored high for their method of fungal exposure, only four of these (7%)^45^, ^67^, ^84^, ^89^ scored high for both. Less than half of the studies scored moderate or high for confirmation of targets (*n* = 28, 46%), a physiologically relevant route of exposure in vitro (*n* = 13, 29%), or measurement of cytotoxicity in vitro (*n* = 13, 29% with 60% scoring very low). The full breakdown of individual scores can be found in Supporting Information S5.

## 4 DISCUSSION

This review sought to bring together the current understanding of mechanisms involved in the allergic response to fungi in the airway epithelium and identify knowledge gaps for future research. Despite large variations in the experimental models, methods and fungal allergens used, it was possible to identify potential mechanisms of interest in fungal induced allergy. Although, we also included ‘macrophage’ as a search term to understand events occurring at the air‐interface within the lung (including tissue resident lung macrophages), using our weight of evidence approach, none of the included studies looked at the role of tissue resident lung macrophages.

### 4.1 Models

A wide range of in vitro models were used including different cell types with varying degrees of differentiation. Epithelial cells cultured at ALI for at least 21 days have tight junctions, increased barrier integrity and a cytoprotective mucous layer as occurs in vivo^93^ and can respond differently to fungal allergens compared to undifferentiated monolayer cultures even if they are derived from the same cell line.^67^ For example, four times the concentration of *Alternaria* extract was required to elicit a response in ALI versus monolayer epithelial cell cultures.^67^ Differentiated ALI cultures can provide a more physiologically relevant system; however, they are expensive, time consuming and require a greater level of expertise compared to undifferentiated monolayer cell models.

Studies using in vivo models were even more complex, with different sensitisations and/or challenge methods being used. Daines et al. used an asthma model where mice were administered *Alternaria* culture filters every other day for a total of three exposures.^45^ In contrast, Gao et al. used an asthma model where rats were sensitised using ovalbumin  and then administered *Aspergillus fumigatus* spores twice a week for either 1, 3 or 5 weeks^49^ This made comparisons across studies difficult and was further compounded by the use of KO animals.

Mice do not develop allergic airway disease spontaneously, so they must be artificially induced and are often done so in two phases (sensitisation then challenge), representative of the allergic immune response seen in humans.^94^ Therefore, sensitisation to a fungal allergen followed by subsequent challenge(s) with the same fungal allergen may be regarded as the most representative model of fungal allergy development. In addition, inhalation or intranasal instillation of allergens would also best mimic human exposure to airborne fungal allergens^94^, ^95^ and should be taken into account when developing in vivo models for AAD.

### 4.2 Different fungi/fungal components

The few studies (*n* = 9, 15%) that compared different fungal species suggest that they can induce different responses in airway epithelia. For example, while 48 h exposure to both *Aspergillus* and *Alternaria* extracts stimulated IL6 in primary HNE cells, only *Alternaria* extract enhanced IL33 and TSLP.^85^ A similar induction of IL6 and lack of IL33 or TSLP was observed following a 24 h exposure to *Aspergillus* extract in a separate study using mouse lung epithelial cells.^73^ In contrast, a 1 h exposure with the same concentration of *Aspergillus* extract (purchased from the same supplier) increased IL33 in HBE cells.^88^ This discrepancy may be due to the different exposure times and may indicate that *Aspergillus* extract is only capable of inducing IL33 in the short term. Alternatively, it could simply be due to the use of cells from different species and parts of the airways; different batches of extract, which are known to vary widely and lead to diverse responses,^53^, ^96^ and/or different doses in terms of both actual concentrations and how concentrations are reported (e.g. ug/mL preparation, total protein weight etc.).

Different fungi also led to contrasting effects on other pathways. While the serine protease activity within *Aspergillus* extract induced mucin secretion and tumour necrosis factor‐alpha converting enzyme (TACE) activity in a bronchial epithelial cell line (NCI‐H292), *Alternaria* extract did not.^75^ In contrast, *Alternaria* exhibited serine protease activity similar to that of *Aspergillus* extract in immortalised and primary HBE and HNE cells.^67^, ^81^ These differences may be explained by the fact that these studies measured different endpoints in different cell types and used different protease inhibitors.

Different components of the same fungi also induced diverse responses. Immortalised mouse lung epithelial cells exposed to both heat‐inactivated and live *Aspergillus* conidia for 6, 24 and 48 h showed increased IL25, IL33 and TSLP compared to controls.^60^ This is in direct contrast to the results described above where *Aspergillus* extract did not induce IL25, IL33 or TSLP in either primary HNE or mouse lung epithelial cells.^73^, ^85^, ^88^

This suggests that epithelial cells have diverse responses to different fungi and fungal components. Unfortunately, the heterogeneity between studies and lack of studies directly comparing these variables prevents the identification of cell‐, fungi‐, or fungal component‐specific allergic responses.

### 4.3 Potential mechanisms of fungal‐induced allergy

By collating and comparing the data extracted from the 61 studies, potential mechanisms within airway epithelial cells that drive fungal‐induced allergic responses (Figure 3) have been identified. However, it should be noted that many of the studies within this review used fungal CFE as an exposure compound in contrast to spores, which may not be a physiologically relevant method of exposure for humans on a day‐to‐day basis.^16^

Epithelial barrier disruption is a key mechanism in AAD and has been identified as a target mechanism. As many reviews have been published on the disrupted epithelial barrier in allergy and asthma,^18^, ^20^, ^97^ we have not included a section on the epithelial barrier in this review and included information related to fungi in the sections below.

#### 4.3.1 PAR2‐dependent allergic response

Fungal proteases and PAR2 were identified as key contributors to fungal allergy in 19 (31%) and 13 (21%) studies, the 1st and joint 3rd most studied molecules respectively. Fungal proteases have been shown to promote mucin production, goblet cell hyperplasia and AHR.^98^ Heat inactivation and protease inhibition confirmed that fungal proteases activated the PAR2 receptor in both bronchial and small airway cell lines as well as primary HNE cells.^37^, ^43^, ^81^ *PAR2* had increased expression in lung epithelia compared to *PAR1*, *PAR3* and *PAR4*,^37^, ^43^, ^81^ suggesting it is the key PAR within the airways. *PAR2* was upregulated in primary HNE cells from asthmatic patients compared to healthy controls.^81^ PAR2 is known to play a role in inflammation and inflammatory cell recruitment as part of the disease processes within multiple organ systems, including the respiratory system.^99^

PAR2 is a G protein coupled receptor and fungal proteases cleave the NH_2_ terminus, which then binds to a region within extracellular loop 2 to activate the G‐protein.^11^, ^100^ While trypsin‐like serine proteases are generally regarded as the main activators of PAR2,^62^, ^99^, ^101^ fungal cysteine^74^ and aspartate^68^ as well as serine^37^, ^43^ proteases all activated the PAR2 receptor in a range of HBE cell lines. In keeping with the wider literature on inflammatory disease, which suggests that matrix metalloprotease‐9 (MMP9) can activate PAR2 in airway cells, *MMP9* was upregulated in *A. fumigatus* sensitised mice.^39^

**FIGURE 5:**
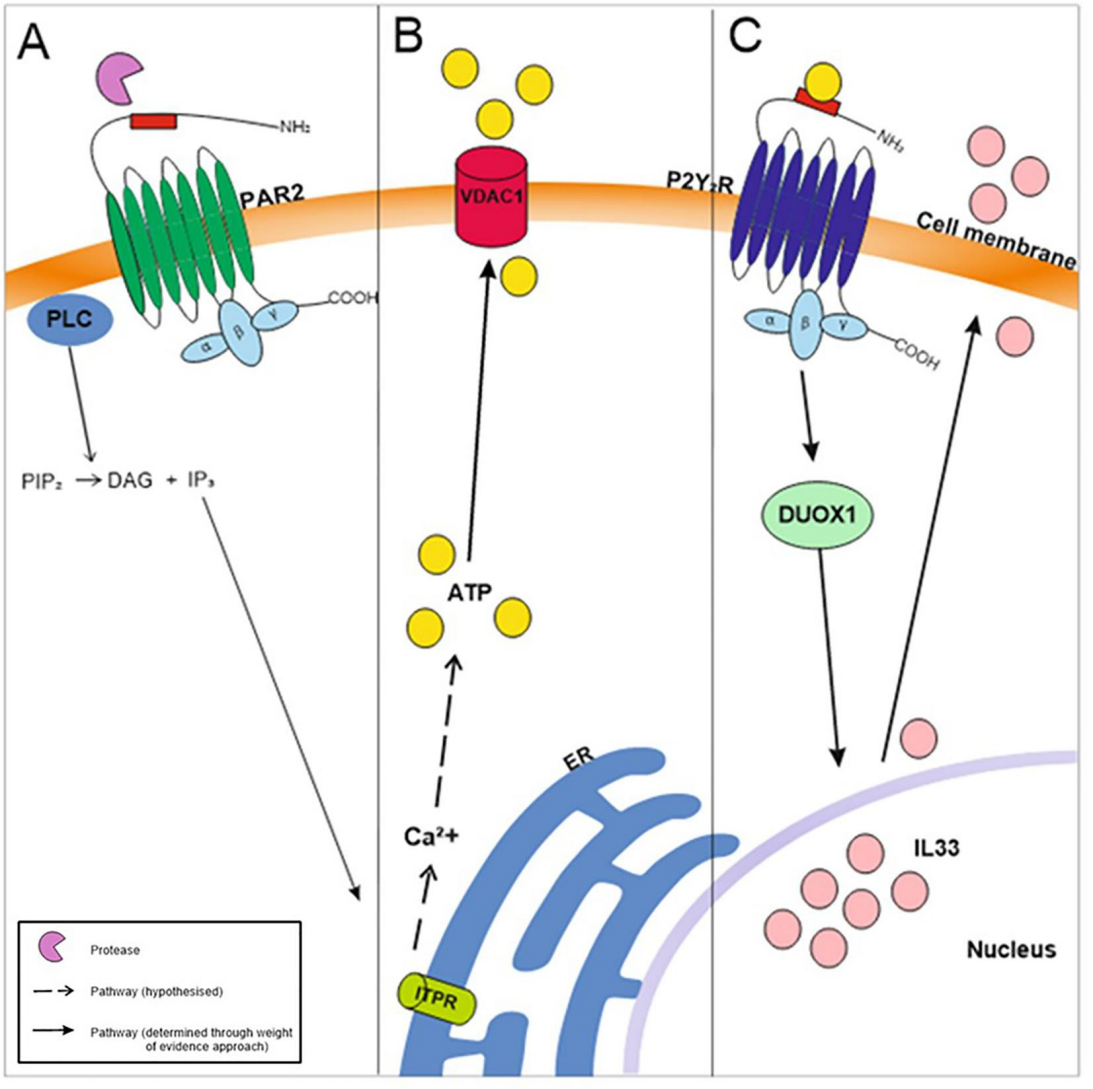
Overview of PAR2 activation and purinergic receptor‐dependent release of IL33. (A) PAR2 is activated by fungal proteases cleaving the extracellular domain. PAR2 activates PLC which cleaves PIP_2_ into DAG and IP_3_. IP_3_ binds to ITPR receptors on the ER membrane. (B) Increases in intracellular calcium induce the release of ATP through the VDAC1 transmembrane channel. (C) Extracellular ATP binds to and activates the P2Y_2_R receptor. This activates DUOX1, which mediates the movement of IL33 from the nucleus into the cytoplasm, where it is ultimately released from the cell. ATP, adenosine triphosphate; Ca^2+^, calcium; DAG, diacylglycerol; DUOX1, dual oxidase 1; ER, endoplasmic reticulum; IL33, interleukin 33; IP_3_, inositol triphosphate; ITPR, inositol 1,4,5‐trisphosphate receptor; P2Y_2_R, P2 purinergic receptor 2; PAR2, protease activated receptor 2; PLC, phospholipase C; PIP_2_, phosphatidylinositol 4,5‐bisphosphate; VDAC1, voltage‐dependent anion channel 1.

Activated PAR2 G protein induces intracellular calcium release^37^, ^41^, ^43^, ^68^ via phospholipase C (PLC),^43^ and adenosine triphosphate (ATP) release^74^, ^77^, ^81^ via exocytosis.^74^ Both intracellular calcium and ATP release was dependent on *Alternaria* dose, indicating that a minimum dose of protease is needed to activate the pathway.^68^, ^74^ *Alternaria alternata* extract also stimulated the generation of inositol trisphosphate (IP_3_) and subsequent release of calcium from the endoplasmic reticulum (ER) through inositol 1,4,5‐trisphosphate receptor (ITPR) channels^41^ (Figure 5A), and ATP via the voltage‐dependent anion channel 1 (VDAC1).^86^ However, whether either of these occurred via protease induced PAR2 activation was not investigated.

Fungal protease‐induced intracellular calcium release led to the production and secretion of cytokines (IL8, IL6) and mucins (MUC5AC, MUC5B).^43^, ^68^, ^77^, ^91^ IL8 release was dependent on both PLC activation and extracellular signal‐regulated kinase (ERK) phosphorylation,^43^ part of the mitogen‐activated protein kinase (MAPK) signalling pathway. The role of MAPK signalling is further supported by a more recent study in which direct activation of PAR2 by fungal proteases led to eosinophil recruitment and mucus overproduction.^98^

Both calcium signalling and ATP release following fungal exposure were also involved in the subsequent secretion of IL33^41^, ^63^, ^81^, ^88^ and other cytokines (IL5, IL13).^88^ It is possible that such cytokine release is a direct downstream consequence of PAR2 activation, and earlier work has demonstrated that the release of TSLP in a human bronchia cell line (BEAS‐2B) exposed to *Alternaria* extract involved PAR2.^62^ However, since the included studies did not investigate the dependence of the initial calcium or ATP release on fungal proteases or PAR2, their corresponding downstream pathways are discussed in separate sections below.

Fungal proteases also disrupted tight junctions between epithelial cells and reduced epithelial barrier integrity in HBE and HNE cells,^42^, ^67^, ^84^ with reduced tight junction (TJ) molecule expression (zonal occludins [ZO‐1], occludin and claudin)^84^ and degraded occludin^87^ and e‐cadherin^89^ proteins (adherens junction [AJ]). While these studies linked disruption of TJs and AJs to fungal proteases, activation of PAR2 was not investigated.

It should be noted, however, that inhibiting PAR2 in HNE and HBE cells did not completely reduce intracellular calcium or ATP release, cytokine or mucin secretion, or inflammation, suggesting additional PAR2‐independent mechanism(s) for allergic responses.^45^, ^74^, ^81^, ^91^ This is supported by recent studies showing non‐protease activation of the MAPK pathway,^98^ and attenuated not abrogated responses in PAR‐deficient mice.^13^

#### 4.3.2 EGFR pathway

A role for the epidermal growth factor receptor (EGFR) pathway in fungal allergy was supported in 6 (10%) of the studies.^45^, ^49^, ^55^, ^75^, ^81^, ^91^ The EGFR pathway plays a role in mucin production, airway epithelial repair and airway remodelling in response to disruption or injury to the epithelial layer.^102^, ^103^, ^104^, ^105^ Activation of EGFR through fungal allergens increased expression of *MUC5AC* and mucus production,^75^, ^91^ induced release of cytokines IL33, IL6 and IL8^45^, ^55^ and was associated with increased injury, shedding of epithelial cells, airway resistance and hyperresponsiveness.^49^ Although EGFR expression in the epithelium is limited,^102^ EGFR protein expression was upregulated in rats in response to *A. fumigatus* spore inhalation^49^ and is upregulated in asthmatics^106^ and other chronic airway diseases.^105^ While the EGFR pathway is associated with epithelial barrier wound repair and disruption in barrier integrity following exposure to other allergens, cigarette smoke or mechanical injury,^103^, ^107^, ^108^, ^109^, ^110^ none of the studies identified here examined EGFR signalling and barrier integrity simultaneously.

EGFR is a transmembrane tyrosine kinase that is activated by several ligands, including epidermal growth factor (EGF), heparin‐binding EGF (hb‐EGF), transforming growth factor α (TGF‐α) and amphiregulin.^102^, ^103^, ^111^ An increased level of both EGF and TGF‐α was observed in the bronchoalveolar lavage fluid (BALF) of rats exposed to *A. fumigatus* spores, with increasing levels of EGF and TGF‐α corresponding to prolonged spore exposure.^49^ EGFR ligands are formed as pro‐ligands or transmembrane precursors and are cleaved by proteases, namely MMPs and ‘a disentegrin and metalloprotease’ (ADAM) proteases, to release the mature ligand.^102^, ^105^, ^108^ It is not clear from the studies identified here whether fungal proteases are capable of cleaving these ligands, and therefore activating the EGFR pathway directly. However, the protease TACE, a member of ADAM family (ADAM 17), was activated by the serine protease activity of *A. fumigatus* in lung carcinoma cells, which in turn cleaved TGF‐α from the cellular membrane.^75^ This is in keeping with a study of the cellular responses to cigarette smoke exposure, where ADAM17 and MMP9 were shown to cleave pro‐EGFR ligands and induce mucin expression.^108^ It is therefore possible that, in addition to cleaving and activating PAR2, fungal proteases can also cleave and activate EGFR ligands.

Following ligand‐dependent activation, autophosphorylation of the receptor occurs at specific tyrosine sites, leading to dimerisation with another EGFR receptor.^105^, ^111^ EGFR activation and subsequent phosphorylation in epithelial cell physiology, disease and repair can also be ligand‐independent,^102^, ^105^ potentially mediated by oxidative stress mechanisms.^102^ Evidence for fungal‐induced ligand‐independent EGFR activation was provided in mouse tracheal cells, where EGFR was rapidly phosphorylated (within 15 min) in response to *Alternaria* extract in the absence of EGFR ligands.^55^ Mechanical and chemical injury and wound repair studies have reported that such ligand‐independent activation of EGFR is mediated by nicotinamide adenine dinucleotide phosphate‐reduced (NADPH) oxidase dual oxidase 1 (DUOX1)^110^ with non‐receptor tyrosine kinases of the src family (Src) playing a role.^109^ DUOX1‐dependent phosphorylation of Src and EGFR in mice exposed to *Alternaria* extract demonstrates a similar role for DUOX1 in fungal‐induced allergy.^55^

As shown in a wide range of airway physiology and disease studies, phosphorylated tyrosine residues on activated EGFR act as docking sites for signalling molecules that initiate downstream signalling cascades such as MAPK,^108^, ^111^ Src, class 1 phosphoinositide 3‐kinases (PI3K)/Protein kinase B (Akt), and PLC pathways.^105^, ^111^ While activation of the MAPK pathway through EGFR phosphorylation has been implicated in mucin production in asthma^111^ and in response to cigarette smoke exposure,^108^ the mechanism appears more complex in fungal‐induced allergy. EGFR activity was required for mucin expression in lung carcinoma cells treated with *A. fumigatus* extract, but no increase in the level of phosphorylated EGFR was observed.^91^ Instead, *Aspergillus* proteases induced increased mucin production by activating Ras and phosphorylating ERK, with ERK1/2 activation likely achieved through intracellular calcium flux.^91^ The downstream effects of activation of signalling pathways PI3K and PLC are discussed in other sections. In addition, EGFR activation was associated with fungal‐induced secretion of IL33^55^, ^81^ (see section below), and ATP has also been associated with activating EGFR in the wider literature.^109^

#### 4.3.3 ATP and purinergic receptor‐dependent release of IL33

IL33 was identified as the second most studied molecule in this review (*n* = 18, 30%). It is an epithelial‐derived cytokine that plays an integral role in activating type 2 immunity in response to allergens.^112^ IL33 was rapidly released upon exposure to *Alternaria*, reaching maximal levels within 2 h in HBE cells^55^ and within 1 h in BALF in an *Alternaria*‐exposed mouse model.^63^ In addition, IL33 was released in response to PAR2 agonists, and both protease and PAR2 inhibitors reduced IL33 secretion,^81^ suggesting that fungal protease‐induced IL33 secretion may (at least in part) involve activation of PAR2 receptors.^11^, ^113^

IL33 was linked with ATP,^63^, ^81^, ^86^, ^88^ calcium signalling^41^, ^55^, ^63^, ^88^ and P2Y_2_R^55^, ^63^, ^81^ in 4, 4 and 3 studies, respectively, and all were shown to be upstream of IL33 release.^55^, ^63^, ^81^, ^88^ Exposure of HBE cells to *Alternaria* led to a sustained increase in ATP^63^, ^74^, ^77^ and a rapid increase in intracellular calcium levels starting in the nuclear and perinuclear region and expanded to the entire cell within 6 min^63^ When calcium influx was abolished, the *Alternaria* associated response was reduced.^63^, ^74^ There is a strong evolutionary link between calcium and ATP signalling,^114^ and these signalling molecules were identified in 13 (21%) and 9 (15%) studies, respectively.

ATP is known as a cellular energy store. However, it has also signalling functions within the inflammatory response as a DAMP and is released from the cell in response to cellular injury.^18^, ^115^, ^116^ ATP production and release is induced by fungal proteases^11^ and the use of serine and cysteine protease inhibitors reduced *Alternaria*‐associated increases in ATP release from HBE cells.^74^, ^77^ ATP release across the cell membrane can occur through exocytosis and transmembrane channels.^116^ In response to fungal allergens, ATP was released via both exocytosis^74^ or VDAC1 in the apical membrane of HBE cells^86^ (Figure 5B).

ATP is part of purinergic signalling and upon release activates purinergic receptors on the cell surface.^116^, ^117^ The epithelial P2Y_2_ receptor subtype 2 (P2Y_2_R) was the most studied purinergic receptor, identified in 4 (7%) studies. ATP activated P2Y_2_R in both primary HNE and HBE cells^55^, ^81^ and was shown to be the predominant purinergic receptor in asthmatic mouse tracheal cells.^40^ P2Y receptors are another family of membrane bound, G‐protein coupled receptors that, upon activation, mobilise intracellular calcium stores via PLC.^117^ Inhibition and siRNA studies confirmed *Alternaria*‐induced IL33 release was dependent on P2Y_2_R activation, through DUOX1‐dependent production of hydrogen peroxide (H_2_O_2_).^55^, ^63^, ^81^ Silencing of *DUOX1* decreased *Alternaria*‐induced IL33 release but not ATP levels in mice,^55^ demonstrating that DUOX1 is downstream of ATP signalling. DUOX1 stimulated the rapid production of H_2_O_2_ and a dose‐dependent relationship between H_2_O_2_ and IL33 release was observed in HBE cells by direct stimulation with H_2_O_2_ ^55^ (Figure 5C). Activation of DUOX1 and IL33 release was also shown to be dependent on calcium signalling.^55^ Since H_2_O_2_ production and subsequent IL33 release were blocked by transient receptor potential vanilloid 1 (TRPV1) inhibitors,^81^ TRPV1 calcium channels also appear to be involved in IL33 release via purinergic receptor activated calcium signalling. In addition to IL33 release, wound repair studies show that ATP‐dependent activation of DUOX1 can also activate EGFR through production of H_2_O_2_ and direct oxidation of Src.^109^, ^110^

IL33 is constitutively expressed in epithelial cells within the lung and resides, under normal conditions, in the cell nucleus in its full length form.^11^, ^112^ Expression of *IL33* was shown to be mediated by nuclear factor kappa‐light‐chain‐enhancer of activated B cells (NF‐κB), activator protein 1 (AP‐1) and MAPK transcription factors.^85^ Indeed, IL33 was confirmed to be localised within the nuclei of HBE cells at rest.^63^ Upon exposure to *Alternaria*, IL33 rapidly moved into the cytoplasm, where it was then released into the cell supernatant.^63^ This is in agreement with other studies demonstrating induction of IL33 release by proteolytic allergens.^18^, ^113^ IL33 was originally thought to be released upon cell injury or necrosis.^11^, ^112^ However, following fungal allergen exposure, IL33 was released into supernatant without apparent cell necrosis.^55^, ^63^ It is not clear from the included studies whether IL33 can be activated by fungal proteases through proteolytic cleavage, however, other studies have shown that environmental allergens including *Alternaria* can cleave full length IL33 directly after release from the cell.^118^, ^119^ Active IL33 recruits innate lymphoid cells (ILC2) to the airway, and binds to the suppression of tumorigenicity 2 (ST2) receptor (also known as IL1RL‐1) located on the surface of ILC2s, stimulating further cytokine production including IL13 and IL5 (identified in 10 [16%] and 5 [8%] studies, respectively).^120^, ^121^, ^122^ Other epithelial derived cytokines such as IL25 and TSLP (both identified in 10 [16%] studies) are also capable of recruiting ILC2s.^120^, ^121^ These cytokines, along with IL4 (identified in 8 [13%] studies), all play a role in driving specific characteristics of allergic inflammation (as described in Figure S1),^120^, ^121^ with some evidence that these cytokines were dependent on signal transducer and activator of transcription 6 (STAT6) signalling^47^, ^48^ and NF‐κB.^66^, ^70^ Inhibition of IL33 in various studies also reported subsequent decreases in IL13, IL5 and ILC2 numbers, following exposure to *Alternaria*.^41^, ^55^, ^63^ This is in keeping with reviews that describe *Alternaria* inducing type 2 cytokines through IL33.^18^, ^122^

#### 4.3.4 Oxidative stress

Oxidative stress pathways, including reactive oxygen species (ROS), ER stress and the unfolded protein response (UPR), were identified in nine studies (15%). Oxidative stress has long been thought to be involved in inflammatory diseases, and our current understanding of how oxidative stress affects AAD is continuing to evolve.^123^, ^124^

**FIGURE 6:**
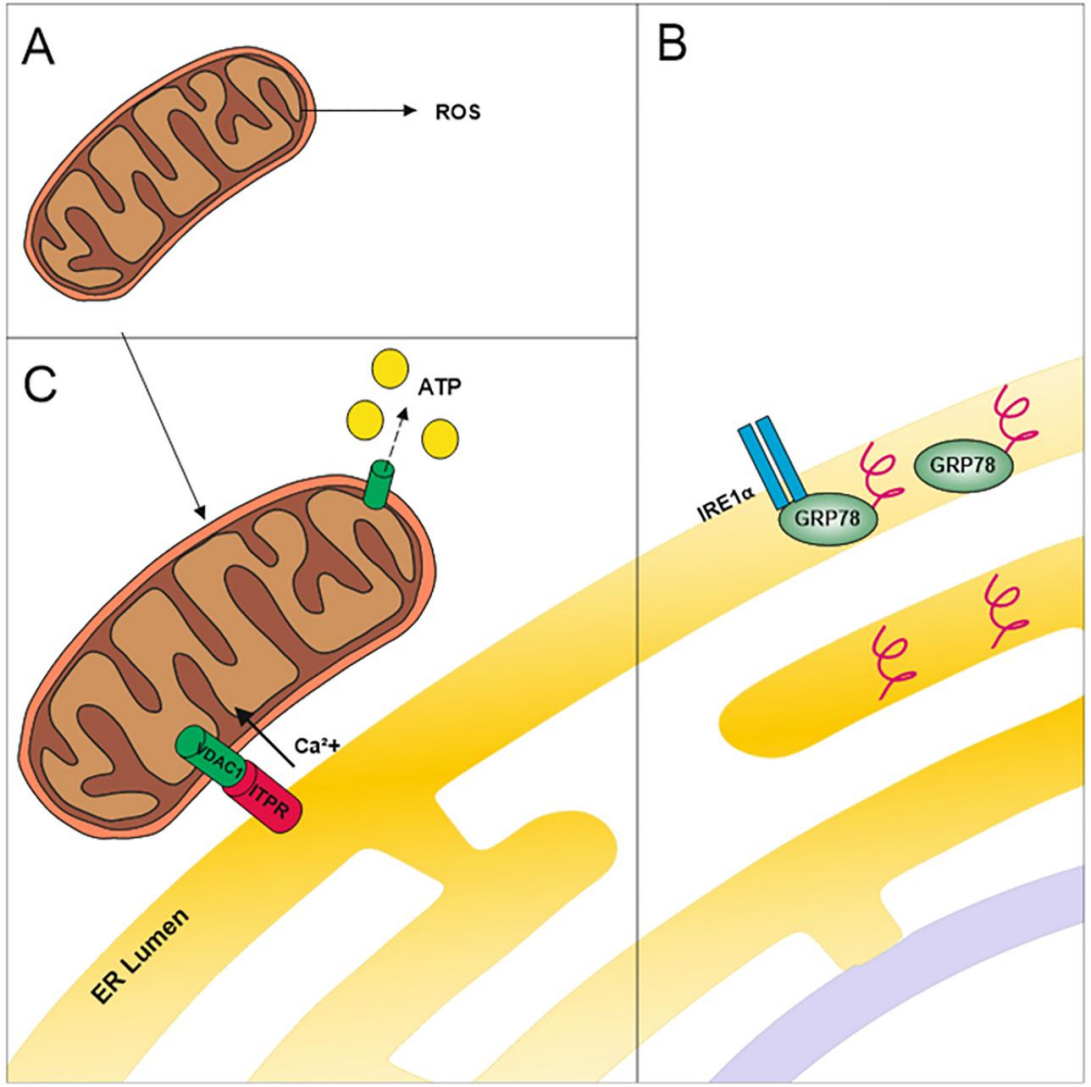
Oxidative stress pathways upon exposure to fungal allergens. (A) Upon stimulation with fungal allergens, levels of mitochondrial ROS increase, which have roles in other signalling pathways. (B) Increase in unfolded proteins in the ER increases expression of GRP78, a protein chaperone, which binds to the unfolded proteins and activates the ER transmembrane channel, IRE1α. (C) Mitochondria swell and move closer to the ER coming into close proximity, where the ITPR ER channel and the VDAC1 mitochondrial channel, co‐localise and transfer calcium from the ER to the mitochondria, potentially inducing the release of ATP. ATP, adenosine triphosphate; Ca^2+^, calcium; ER, endoplasmic reticulum; GRP78, glucose regulated protein 78; IRE1α, inositol‐requiring enzyme‐1α; ITPR, inositol 1,4,5‐trisphosphate receptor; ROS, reactive oxygen species; VDAC1, voltage‐dependent anion channel 1.

Oxidative stress is a term used to describe the overproduction of ROS and impaired redox balance.^124^, ^125^ Upon stimulation by exposure to allergens (pollen and HDM), air pollution and other inhaled particles, levels of ROS increase and are often part of the signalling cascades within the inflammatory response^124^ (Figure 6A). Little is known about the induction of ROS through fungal allergens (with the exception of H_2_O_2_ production, a ROS molecule, as discussed in the previous section), although seven studies have shown that ROS levels increase in response to fungal exposure in lung epithelial cells^61^, ^84^, ^88^, ^91^, ^92^ and in mouse models.^65^, ^66^ Here we will focus on these studies and refer the interested reader to other recent reviews for a more in‐depth look into oxidative stress associated with non‐fungal allergens and air pollution.^124^, ^126^, ^127^, ^128^

Fungal proteases were implicated in the increased expression of ROS.^42^, ^61^, ^84^, ^92^ Analysis of protein expression changes in response to *Penicillium* protease exposure in a mouse model found that 20% of differentially expressed proteins were related to oxidoreduction and 8% to protein folding.^42^ In other studies, *Alternaria* serine proteases strongly induced ROS production in differentiated primary HNE cells^84^ and *Aspergillus oryzae* proteases induced mitochondrial ROS (mtROS) in HBE cells.^61^ However, protease inhibitors did not attenuate the downstream effects of *Alternaria* exposure in HBE cells, indicating that other fungal components can also generate ROS.^92^ Downstream effects of increased ROS include epithelial barrier disruption, release of cytokines, ER stress and the UPR, and inflammasome assembly.^124^, ^126^, ^129^, ^130^

Two studies looked at the role of fungal‐induced oxidative stress in barrier integrity.^84^, ^92^ A decrease in trans‐epithelial resistance (TEER) was observed in HNE^84^ and HBE^92^ cells following exposure to *Alternaria* extract, which was reversed by the ROS scavenger glutathione in both studies. While reduction of TJ and AJ proteins was implicated in loss of barrier integrity in HNE cells,^84^ the levels of both the TJ and AJ proteins remained unchanged in HBE cells.^92^ This suggests that ROS can affect the epithelial barrier in multiple ways.

One study identified ROS as an inducer of transforming growth factor β (TGF‐β) which can inhibit antioxidant enzymes, further increasing oxidative stress in asthma.^125^ *Aspergillus* proteases increased inflammatory mediators, including TGF‐β and other cytokines (IL6 and IL1β), in HBE cells, which were at least partially driven by increased mtROS.^61^ In addition, ROS can activate the MAPK, small mothers against decapentaplegic (SMAD) and PI3K pathways.^125^ The same *Aspergillus* proteases also activated MAPK signalling through increased c‐Jun N‐terminal kinases (JNK) and ERK phosphorylation.^61^ This activation was reduced with the use of a ROS scavenger, confirming that mtROS‐induced MAPK signalling.^61^ Exposure to *Alternaria* extract also produced rapid increases in ATP, intracellular calcium and IL33 secretion, all of which were inhibited by ROS scavengers.^88^, ^92^

Normal cellular defences against oxidative stress include mitochondrial uncoupling protein 2 (UCP2), which mediates proton leakage^131^ and may reduce mtROS. Silencing of *UCP2* in HBE cells resulted in increased production of mtROS, and inhibition of both TGF‐β and SMAD4 reversed the decrease in UCP2 induced by *Aspergillus* proteases.^61^ This implies that *Aspergillus* proteases inhibit UCP2 and stimulate mtROS by upregulating TGF‐β and SMAD4.

Outside the mitochondria, unfolded or misfolded proteins can accumulate in the ER, causing ER stress. This activates the UPR, which has been implicated in asthma.^128^, ^132^, ^133^ The UPR contains a number of proteins and transcription factors including the protein chaperone glucose‐regulating protein 78 (GRP78) that binds to misfolded proteins to activate the UPR (identified in 2 studies, 3%),^65^, ^66^ the transcription factor C/EBP homologous protein (CHOP) that can regulate apoptosis (identified in 1 study)^66^; and the transmembrane receptor inositol‐requiring enzyme‐1α (IRE1α) that senses misfolded proteins through GRP78 (identified in 1 study)^66^ (Figure 6B).

The ER stress markers GRP78 and CHOP were increased in mice^65^, ^66^ and murine tracheal cells exposed to *Aspergillus* extract.^66^ These increases were reduced in the presence of a ROS scavenger,^66^ suggesting that ROS may also play a role in inducing ER stress. Wider evidence suggests that PI3K signalling is involved in Type 2 immune responses, with the PI3K‐δ subunit in particular mediating ROS‐driven allergic inflammation and ER stress.^128^, ^134^ Evidence for a similar mechanism in fungal allergy was provided by two studies demonstrating increased PI3K‐δ mRNA, ER stress markers and impaired protein folding in murine tracheal cells following *Aspergillus* exposure that were reduced upon inhibition of PI3K‐δ.^65^, ^66^ As well as reducing ER stress, inhibition of PI3K‐δ also decreased *Aspergillus*‐induced mtROS, protein oxidation, and membrane lipid peroxidation, which restored redox balance, and, in turn, reduced AHR, infiltration of eosinophils and mucin expression.^58^, ^65^, ^66^ The same was seen when a ROS scavenger was used,^66^ further confirming the role of PI3K‐δ and mtROS in oxidative stress and the allergic response to fungi.

Allergen exposure can also affect ER and mitochondrial morphology, resulting in potential crosstalk between the ER and mitochondria.^124^ In *Aspergillus* exposed mice, there was a decrease in membrane fluidity, and an aberrant morphology in the ER membrane as well as swollen mitochondria with a decreased distance between the two organelles.^65^ Further investigation in mouse lung tissue confirmed that ER‐associated ITPR transmembrane channels and the mitochondrial outer membrane channel VDAC1 became more closely associated in response to Aspergillus exposure.^65^ Such sites of physical contact are known as mitochondria‐associated ER membranes (MAMs)^127^ (Figure 6C) and ER‐mitochondrial crosstalk may play a role in the allergic response to fungi. One hypothesis is that the movement of calcium between the ER and the mitochondria causes the release of ATP from the mitochondria into the cytosol. In support of this, calcium was released from the ER, uptake of calcium into the mitochondria increased and mitochondrial ATP levels were reduced upon exposure of HBE cells (BEAS‐2B) to *Aspergillus*.^65^ All of these *Aspergillus*‐induced changes were reversed or reduced in the presence of PI3K‐δ or ER stress inhibitors.^65^

**FIGURE 7:**
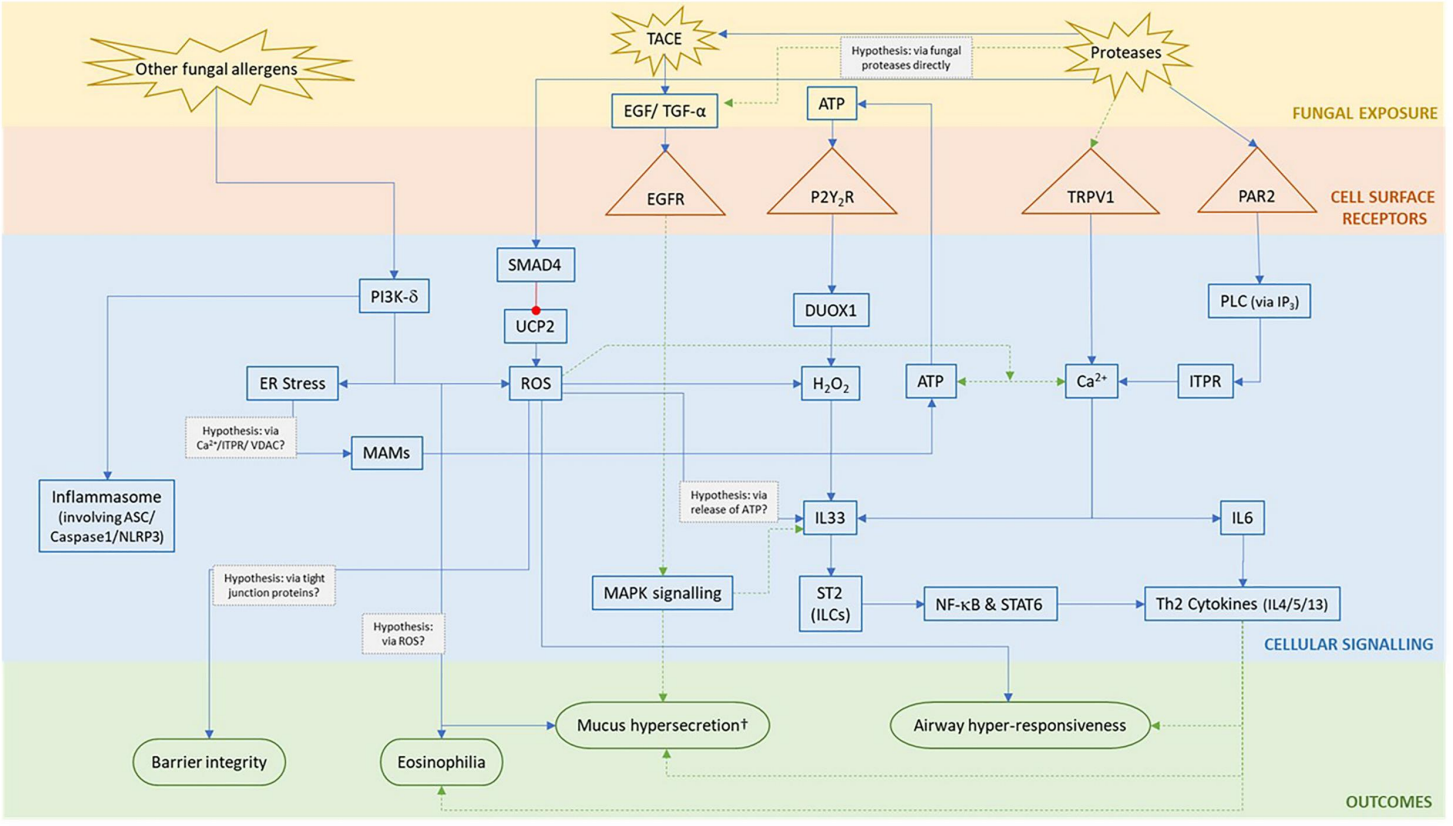
Graphical summary of the cellular mechanisms in response to fungal allergens identified in this review. Blue lines indicate where studies have provided direct evidence of these pathways. Green dotted lines indicate potential pathways where there is no direct evidence. ^†^Within this figure, mucus hypersecretion also includes other mucus‐related outcomes include goblet cell hyperplasia and goblet cell metaplasia. ASC, apoptosis‐associated speck‐like protein containing a CARD; ATP, adenosine triphosphate; Ca^2+^, calcium; DUOX, dual oxidase; EGF, epidermal growth factor; EGFR, epidermal growth factor receptor; ER, endoplasmic reticulum; H_2_O_2_, hydrogen peroxide; IL, interleukin; ITPR, inositol 1,4,5‐trisphosphate receptors; MAM, mitochondria associated ER membranes; MAPK, mitogen‐activated protein kinases; NF‐κB, nuclear factor kappa‐light‐chain‐enhancer of activated B cells; NLRP, nucleotide‐binding oligomerization domain, leucine rich repeat and pyrin domain containing; P2YR2, P2 purinergic receptor 2; PAR, protease activated receptor; PI3K, phosphoinositide 3‐kinase; PLC, phospholipase C; ROS, reactive oxygen species; SMAD, small mothers against decapentaplegic; ST2, suppression of tumourigenicity 2; STAT, signal transducer and activator of transcription; TACE, tumour necrosis factor‐alpha converting enzyme; Th, T helper; TRPV, transient receptor potential vanilloid; UCP, uncoupling protein; VDAC, voltage‐dependent anion channel.

An overview of all the potential mechanisms driving fungal‐induced allergy in the lung epithelium and how they interact can be found in Figure 7.

### 4.4 Recommendations for future work

This review of the current literature, combined with the quality assessment designed to highlight the strengths and weaknesses of the included studies in understanding the cellular mechanisms that drive the allergic inflammatory response to fungal allergens in the lung epithelium, has identified several knowledge gaps, which the following recommendations aim to address:

1. Comparison of fungal species and allergenic fungal components
  There are a large number of fungal species that are considered to be allergenic, however, the majority of studies focused on *Aspergillus* and *Alternaria*. At present, there is no comprehensive comparison of responses to different fungal allergens or species. Studies should be expanded to include more species and use direct comparisons within the same physiologically relevant model to determine whether there are common and/or specific mechanisms driving fungal allergy. In addition, studies need to directly compare the allergic response(s) to different allergenic components (spores, live fungus, hyphal fragments, culture extract or purified proteases) to determine which components are allergic and how they activate allergic pathways. Many studies have used fungal extracts, with proteases being identified as a major allergen. However, it is unclear if and how individuals are exposed to fungal proteases. Have proteases been identified as a major contributor to allergy as an artefact of the experimental design? Can individuals inhale proteases, and can we sample this? Or is it other allergens on inhaled spores and hyphal fragments that trigger these allergic responses? Better characterisation of what fungal allergens individuals are exposed to is essential to interpret, and design future mechanistic studies with greater relevance to human health.
2. Understanding allergic response in AAD in vitro models
  Surprisingly few in vitro studies included and compared the allergic responses to fungi in normal compared to disease states *in vitro*. The disease status of individuals can markedly change the way in which the immune system responds to fungal allergens. Those studies that did compare AAD with normal cells demonstrated that epithelial cells from sufferers of AAD responded differently to ‘healthy’ donors. Thus, future in vitro studies should include models of AAD or donor cells/tissue from AAD donors for direct comparison with ‘healthy’ cell (in appropriate and relevant models to the research question i.e. ALI, co‐cultures, organoids, etc.) to fully understand how normal and potentially more susceptible individuals/populations respond to fungal exposures.
3. PAR2‐IL33 mechanisms
  IL33 was one of the top identified mechanisms, along with fungal proteases, within this review. However, it is not clear whether the activation of PAR2 by fungal proteases directly leads to release of IL33. Although there have been studies that imply a link through the release of ATP, further work is required to determine whether fungal proteases induce the release of IL33 through PAR2‐dependent and/or independent mechanisms. This should include determining whether fungal proteases themselves can directly activate IL33 and/or components within the IL33 pathway.
4. EGFR‐mediated barrier integrity
  EGFR signalling was not investigated with respect to fungal‐induced disruption of the epithelial barrier. In the wider literature, there is evidence of a key role for the EGFR pathway in the loss of epithelial barrier integrity and associated repair resulting from non‐fungal allergen‐related injury. Whether EGFR signalling plays a similar role in the disruption of the epithelial barrier following fungal allergy requires further examination.
5. Induction of ROS from fungal allergens
  While there is limited information on how fungal allergens lead to ROS generation and associated oxidative stress responses in fungal‐induced allergy (outside of the role of hydrogen peroxide in IL33 signalling), evidence suggests that ROS contributes to epithelial barrier integrity, protein folding and the formation of MAMs. Both the generation of ROS and the subsequent mechanisms by which it acts upon epithelial cells requires further investigation.

## 5 CONCLUSIONS

In summary, this review has brought together the current literature on fungal allergy in airway epithelia, identifying mechanisms including PAR2, EGFR, ATP and IL33 signalling, and oxidative stress. These pathways can induce the classic hallmarks of allergy including increased mucus production, eosinophilia and AHR. Ambiguity remains, however, due to the heterogeneity of current models and methods and specific gaps in our understanding. Future research should focus on directly comparing allergenic fungal species and fungal components in appropriate and relevant models (i.e. air‐liquid interface, co‐cultures, organoids, etc.) to better understand what is driving the fungal allergy, and the role that the epithelium plays, addressing specific knowledge gaps such as PAR2‐dependent release of IL33, mediation of barrier integrity by EGFR signalling, oxidative stress and ROS generation, and assessing key pathways in AAD models. A better understanding of the mechanisms involved in the allergic response to fungi in both normal and AAD sufferers will help inform future interventions and ultimately reduce the burden of disease.

## AUTHOR CONTRIBUTIONS

**Emma‐Jane Goode**: Data curation (lead); formal analysis (lead); methodology (equal); writing – original draft (lead); writing – review & editing (Lead). **Emma Marczylo**: Conceptualization (lead); data curation (equal); formal analysis (supporting); supervision (lead); writing – original draft (supporting); writing – review & editing (supporting).

## CONFLICT OF INTEREST STATEMENT

The authors declare that they have no relevant conflicts of interest.

## Supporting information

Supporting Information S1

## Data Availability

Data sharing is not applicable to this article as no new data were created or analysed in this study.
