# Supplementary material for: A scoping review: What are the cellular mechanisms that drive the allergic inflammatory response to fungal allergens in the lung epithelium?
Source: Clin Transl Allergy. 2023 Jun 1;13(6):e12252. doi: 10.1002/clt2.12252 (PMC10234180; doi:10.1002/clt2.12252)

**Supplementary information for:**

**What drives allergic inflammatory responses to the known fungal allergens at the epithelial barrier in the lungs?**

Emma-Jane Goode and Emma Marczylo

Toxicology Department, UK Health Security Agency, Harwell Campus, Chilton, Oxfordshire, OX11 0RQ, UK

Contents: Pages:

Supplementary Information 1: Search strategy 2

Supplementary Information 2: Quality Scoring Tool 3

Supplementary Information 3: Overview of included studies – full table 6

Supplementary Information 4: List of target mechanisms 28

Supplementary Information 5: Quality scores for selected studies 30

Supplementary Figure S1: Overview of IL33 induction of Th2 cytokines 33

**Supplementary Information 1:** Search strategy for terms used to perform review of the literature using PubMed database.

(‘Alternaria’ OR ‘Cladosporium’ OR ‘Epicoccum’ OR ‘Aspergillus’ OR ‘Penicillium’ OR ‘Didymella’ OR ‘Pleospora’ OR ‘Sporobolomyces’ OR ‘Tilletiopsis’) AND ('epithelial' OR 'macrophage') AND ('lung' OR 'respiratory' OR ‘airway’ OR ‘inhal*’) AND (allerg* OR 'cystic fibrosis' OR 'asthma' OR 'COPD' OR sensitis* OR inflamma* OR hyperrespons* OR (‘Th2’ OR ‘Th1’ OR ‘Th17’ OR pro-inflam* OR anti-inflam*) NOT 'review'

Inclusion of ‘epithelial’ or ‘macrophage’ search terms were required to focus in on relevant papers. However this meant that some papers with relevant information which did not specifically use this term were not included. Such papers (along with more recent relevant papers or papers investigating general (not allergic) responses to fungal exposure) were subsequently obtained through specific searches of the literature on the key pathways identified.

While a number of different cell types are involved in the allergic response, we focussed primarily on the events occurring at the airway epithelium (including tissue resident macrophages) and mechanisms involved in other cell types will not be discussed in further detail. More information on responses in other cell types are discussed in recent review articles (1, 19, 34, 35).

**Supplementary Information 2: (A)** Quality-scoring tool for assessment of seven domains deemed necessary for understanding the mechanisms of fungal exposure. Table (B) further elaborate on domains D1 and D2 for *in vivo* and *in vitro* studies.

|  | 1. Domain name, description, and quality score classification | | | | | | |
| --- | --- | --- | --- | --- | --- | --- | --- |
| Domain name | D1: Route of exposure | D2: Model Specificity | D3: Inhibition or activation of target mechanisms | D4: Gene and protein expression | D5: Th2 response to fungal allergens | D6: Cytotoxicity (*in vitro* only) | D7: Induction of allergic airways (*in vivo* only) |
| Domain description | Physiologically relevant application of fungal allergens to the experimental model | Physiologically relevant model, cells or tissues of the airways used in the study | Confirmation of target using inhibitors, ligands, knockouts or transfection techniques | Confirmation of gene expression assays with protein expression assays | Evaluation of Th2 response to fungal allergens | Measurement of cytotoxicity in cells after application of fungal allergens | Physiologically relevant method of fungal sensitisation and challenge with confirmation of induced airway inflammation |
| Quality score classification | See Table (B) below for score classification | See Table (B) below for score classification | Higher scores were assigned to studies that confirmed targets using a combination of activating and inhibiting compounds or techniques | Higher scores were assigned to studies which used a combination of protein expression methods to follow up gene expression results | Higher scores were assigned to studies that measured known Th2 cytokines (IL-4, IL-5, IL-13, IL25, IL33 or TSLP) | Higher scores were assigned to studies that used assays to determine if application of fungal allergens caused cytotoxicity in cells | Higher scores were assigned to protocols consisting of fungal sensitisation through an inhalational route followed by fungal challenge, and where allergic inflammation was confirmed in the model |
| HIGH | See Table (B) below for score classification | See Table (B) below for score classification | Used a combination of inhibiting compounds or techniques (i.e., gene silencing) and activating ligands or compounds to confirm the target studied as well as validating further downstream pathways/processes | Used a combination of protein expression methods to follow up gene expression result | Measured 3 or more known type 2 cytokines (IL-4, IL-5, IL-13, IL25, IL33 or TSLP) | Assessed cytotoxicity in cells *in vitro* using two or more viability assays | Animals were sensitised to fungal allergens over a time period followed by challenge with a dose or doses of fungal allergen different to the sensitisation protocol. Allergic inflammation was confirmed using histology/IgE and eosinophil counts |
| MODERATE | See Table (B) below for score classification | See Table (B) below for score classification | Used both inhibiting compounds or techniques (i.e., gene silencing) and activating ligands or compounds to confirm the main target gene/pathway studied but did not validate further downstream pathways/processes | Used one protein expression method to follow up gene expression results | Measured 1 or 2 type 2 cytokines (IL-4, IL-5, IL-13, IL25, IL33 or TSLP) | Assessed cytotoxicity in cells *in vitro* using one viability assay | Animals were sensitised to fungal allergens (without a subsequent challenge) **OR** challenged with one only dose of fungal allergen (without prior sensitisation). Allergic inflammation was confirmed using histology/IgE and eosinophil counts |
| LOW | See Table (B) below for score classification | See Table (B) below for score classification | Used either inhibiting or activating techniques to confirm targets but not both | Only used protein expression methods | Measured non-specific or type 1/17 cytokines/ chemokines including IL6, IL8, IL-1β, TNF-α, IL17 | Assessed cytotoxicity but did not provide methods | Animals were sensitised and challenged with fungal allergens along with other compounds (e.g.alum, OVA, Freud’s adjuvant etc). Allergic inflammation was confirmed using histology, IgE or eosinophil counts |
| VERY LOW | See Table (B) below for score classification | See Table (B) below for score classification | Did not confirm targets with inhibiting or activating techniques | Only looked at gene expression | Did not measure cytokines | Did not assess cytotoxicity | Animals were sensitised (without subsequent challenge) with compounds known or suspected to be involved in the allergic response to fungi **OR** Allergic inflammation was not confirmed in the model |

## **(B)** Domain name, description and quality score classification for D1 and D2

| Domain name | D1: Route of exposure (*in vitro* only) | D1: Route of exposure (*in vivo* only) | D2: Model Specificity *(In vitro* only) | D2: Model Specificity (*in vivo* only) |
| --- | --- | --- | --- | --- |
| Domain description | Physiologically relevant application of fungal allergens to the experimental model | Physiologically relevant model, tissue or cells of the airways used in the study | Physiologically relevant cells or tissues of the airways used in the study | Physiologically relevant model used in the study |
| Quality score classification | Higher scores were given if fungal allergens were applied apically to cells in ALI systems | Higher scores were given if fungal allergens were applied via inhalational route | Higher scores were given if cells or *ex-vivo* tissues were primary human epithelial cells or tissues (either derived directly from patient specimens or purchased) | Higher scores were given if the study focussed on lung epithelial cells or lung epithelial tissues from animal models |
| HIGH | Fungal allergens were applied apically to cells in ALI systems | Fungal allergens were applied via intranasal aspiration or aerosol (nose only) inhalation | Cells/tissues used in the study were primary human epithelial cells and cultured at ALI for 21 days or more **OR** human *ex vivo* tissues (patient biopsies including polyps) or primary animal epithelial cells for further downstream experiments/challenge | Isolated and studied lung epithelial tissues or lung epithelial cells (isolated by FACs or other specific method) |
| MODERATE | Fungal allergens were applied to cells in ALI systems basally/method does not mention how allergens were applied to ALI systems **OR** *ex vivo* tissues were stimulated directly with fungal allergens | Fungal allergens were applied via oropharyngeal or intratracheal aspiration/ instillation. | Cells/tissues used in the study were either primary human epithelial cells cultured at ALI for less than 21 days or monolayer **OR** immortalised human epithelial cell lines (non-tumorigenic) cultured at ALI for 14-21 days **OR** human nasal/bronchial/tracheal *ex vivo* tissues for downstream experiments/challenge not specifically targeting the epithelium | Isolated and studied lung epithelial cells (isolated through non-specific nasal/tracheal/bronchial digestion) or nasal/tracheal/bronchial tissues for staining/downstream experiments |
| LOW | Fungal allergens were applied in media to undifferentiated cells in monolayer **OR** were applied to *in vivo* sensitised models and the subsequent *ex vivo* tissues (used for experimentation) were not further stimulated with fungal allergens | Fungal allergens were applied via intraperitoneal route AND other intranasal, intratracheal or oropharyngeal route | Cells/tissues used in the study were tumorigenic human epithelial cell lines cultured at monolayer **OR** *ex vivo* non-lung tissues for downstream experiments/challenge | Whole lung was used for further experiments/staining **OR** studies homogenised whole lung and analysed lysates |
| VERY LOW | Compounds known or suspected to be involved in the allergic response to fungi were applied (that were not fungal allergens). | Fungal allergens were applied via intraperitoneal route OR compounds known or suspected to be involved in the allergic response to fungi were applied (that were not fungal allergens) | Cells used in the study were tumorigenic human epithelial cell lines cultured at monolayer OR studies used non-lung tissues for downstream challenge or further experiments. | Studies only studied at BALF and AHR |

AHR: airway hyperresponsiveness, ALI: Air-liquid interface, BALF: bronchoalveolar lavage fluid, FACS: fluorescence-activated cell sorting, Ig: Immunoglobulin, IL: interleukin, OVA: ovalbumin, Th: T helper, TSLP: thymic stromal lymphopoietin

**Supplementary Information 3:** Full study data for all 61 studies included in review including target mechanisms, models and outcomes

Potential target mechanisms were defined as any molecule or pathway that was either a) the specific focus of the study, b) a molecule or pathway that was further validated using knockouts, inhibitors or gene silencing/overexpressing techniques, or c) an endpoint molecule where pathways or genes were manipulated to observe the effects on said molecule. Molecules that were investigated using appropriate validation techniques but found not to be involved in specific pathways were still included as potential target mechanisms. Molecules that were used as endpoints to confirm allergic responses in models were not included as potential target mechanisms unless they underwent further investigation.

| Author(s) | Fungus/Fungi | Target mechanisms | Clinical Tissues | Cell/Tissue Model (In Vitro) | Animal Model  (In Vivo) | Outcomes |
| --- | --- | --- | --- | --- | --- | --- |
| Anagnostopoulou et al, 2010 | *Aspergillus fumigatus* extract (AF) | ENaC ion channel, CFTR channel, CaCC channel  STAT6 pathway | N/A | N/A | BALB/c WT and STAT6^-/-^ BALB/c,  adult females 6-8 weeks.  Sensitisation: 20ul of 2mg/ml AFE in 0.9% NaCl, i.t administration 3/ week for 3 weeks.  Controls: 0.9% NaCl | - Allergic inflammation reduced α, β, γ ENaC expression, but reduction abolished in STAT6^-/-^ mice - Bronchial tissues were more absorptive under normal conditions - cAMP mediated Cl^-^ secretion was attenuated by allergic inflammation - STAT6 was directly involved in expression of epithelial ion transport channels in AAD |
| Babiceanu et al, 2013 | *Alternaria alternata* (AA) conidia | Gene expression upon AA exposure | N/A | BEAS-2B cells cultured at monolayer  Fungal dose: 5 x 10^5^ conidia added to media for 24hrs. | N/A | - AA induced selective activation of different innate immunological pathways in ECs - Multiple functional related chemokines and cytokines and related signalling pathways - AA proteins and/or secreted metabolites were potent induces of inflammation |
| Bains et al, 2012 | *Aspergillus fumigatus* (mycelia extract and culture filtrate) | Caveolin-1 (Cav-1) | Patients: mild to severe asthma (n=6), controls (n=6).  Endobronchial biopsies - mild to moderate asthma (n=5), controls (n=5). | Primary bronchial epithelial cells (HpBEC)  Cells not stimulated with fungus. | 6 BALB/c female mice, 6 control mice.  Sensitisation: 200ug AF i.p injection  Challenge: i.n administration 200ug AF in 10ul PBS - 3/ week for 4 weeks. Control: saline | - Demonstrated loss of Cav-1 in asthmatics vs to controls - Evidence of hypercellularity and peri-bronchial fibrosis observed in AF-sensitised mice vs control mice - Staining for Cav-1 decreased in AF sensitised mice vs control mice |
| Bankova et al, 2016 | *Alternaria alternata* culture filtrate | LTE4,  cysteinyl leukotrienes, GPR99 | N/A | *In vitro work not included in final analysis* | Ltc4s^-/^, Cysltr1^-/-^, Cystlr2^-/-^ , Gpr99^-/-^, Fcer1g^-/-^ , Mcpt5/DTA mice. WT litter mates or C57BL/6 age and sex matched, 3-8months of age  Challenge: 30ug AA in 20ul PBS, i.n. application  Controls: PBS only | - AA induced marked swelling, no detectable cellular infiltration, increased mucus secretion in WT mice vs controls - LTC_4_ KO reduced swelling and mucin release - Submucosal oedema and mucin release were absent in AA-treated mast cell-deficient mice but present in AA-treated *Fcer1g*^-/-^ mice - Absence of submucosal swelling and reduced mucin release in *Cysltr1^-/-^* but intact in *Cystlr2^-/-^* mice vs WT - Submucosal swelling and mucin release inhibited in *gpr99^-/-^* mice vs WT - GPR99 was the dominant CysLTR for LTE4, elicited EC secretory function |
| Bickford et al, 2012 | *Aspergillus fumigatus* extract (aerosolised) | cPLA2y, TNFα, ATF-2, c-Jun, p65, USF1 & 2 | N/A | A549 cells, S9 (HBEC) cell line, cultured at monolayer  Fungal dose: 100ug/ml AF applied via media for 12 hrs | C57BL/6J mice  Sensitisation: i.p. injection on day 0, 14 Challenge: aerosolised AF extract on d28, d29 & d30  Controls: sensitised with PBS, challenged with aerosolised AF | - AF sensitisation caused Th2 responses, goblet cell hyperplasia, airway eosinophilia, increased total serum IgE - IFNγ, TNFα, IL1B, IL6, IL10, MCP1, MIP-1α were elevated - cPLA2y (phospholipase A) was induced in a model of allergic asthma (cytosolic phospholipase) via TNFα and NF-κB, in response to fungal sensitisation |
| Boitano et al, 2011 | *Alternaria alternata* – culture filtrate (also heat-inactivated (HI-AA) | PAR2, Ca^2+^,  protease | N/A | 16HBE14o- cells,  HeLa cells (PAR2-containing plasmid), cultured at monolayer Fungal dose: not given but applied apical membrane of monolayer cell culture | BALB/c mice, 6 weeks old, male  Sensitisation: 10ug of AA filtrate in 100ul HBSS, i.n. application, 3 times on d1, d4, d8. Control: HBSS only | - AA sensitisation caused a 16-fold increase in BALF cells, increase in lymphocytes, neutrophils, macrophages and eosinophils - HI-AA & serine protease inhibitor reduced cell counts in BALF - Ca^2+^ response started 20-45s following application of AA - PAR2 had highest level of expression vs PAR1, PAR3 & PAR4 in ECs - AA serine specific protease activity was required to develop lung inflammation & cell recruitment to airways - Serine-specific proteases were responsible for AA-induced Ca^2+^ signalling in ECs |
| Brandt et al, 2008 | *Aspergillus fumigatus* (form unknown) | Surfactant protein D, IL13, IL4, IFNγ | 226 caucasian children with asthma  Control group: 120 caucasians, no history of asthma  No fungal allergens used. | N/A | Sftpd^-/-^ mice, swiss black sftpd^+/+^ used as controls.  Sensitisation: 100ug AF in 50ul saline, i.n. delivery, 2 days/week for two weeks.  Controls: Saline only | - *Sftpd* KO mice: Increased BALF cells including increased macrophages and activated T-cells. No change seen in Th1 and Th2 cytokine levels. Increased CCL17 and CCL2 but IgE and IgG1 lower. SPD deficiency was not associated with decreased IFNγ or increased IL4. Small increase in TNFα - AF exposure: Decreased total BALF cells including eosinophils following exposure in KO mice vs WT. IgE undetectable, IgG1 no sig difference in null vs WT. IL13 increased in WT and KO mice vs saline controls but IL13 lower in KO mice vs WT - Clinical data shows polymorphism in surfactant protein D at position 11, which can reduce risk of asthma susceptibility - *Sfptd* KO increased chemokine expression and SPD deficiency attentuated development of Th2 responses |
| Buckland et al, 2011 | *Aspergillus fumigatus* conidia and , AF antigens as indicated | TREM-1, DAP12, MMP-9, TLR2, CCL17 | N/A | *In vitro work not included in final analysis* | CBA/J, C57BL/6 (TLR2^+/+^), TLR2^-/-^ , female 6-8 weeks old  Sensitisation: i.p. & s.c. injection of AF antigens in FA.  Challenge: 14 days later, weekly i.n. challenge with AF antigen  Experimental: intratracheal exposure to 5.0x10^6 AF conidia  Controls: Saline | - AHR and GCM were enhanced when TREM-1 blocked but reduced in DAP12 overexpression group vs controls. Increased collagen deposition and peri-bronchial inflammation when TREM-1 was blocked but inflammation decreased with DAP12 overexpression. TREM-1 levels increased in BALF and serum during fungal asthma. MMP-9 levels also increased during fungal asthma - BALF IL10 and IL13 lower when TREM-1 was blocked, both IL12 and CCL22 increased. TNFα, IL12, CCL2, CCL3, CCL5, CCL17 and CCL22 were elevated in whole lung tissue when TREM-1 blocked - Innate and adaptive immune responses mediated by TREM-1 were critical for clearance of AF conidia from lungs - Without cell-associated TREM-1, allergic mice exhibited an exacerbated form of lung disease |
| Causton et al, 2015 | *Alternaria alternata* | CARMA3, TSLP, CCL20, GM-CSF, IL8, PAR2, P2Y_2_R | N/A | CARMA3 KO NHBEs, cultured at ALI  Fungal dose: 100ug for 6 hrs Primary Mouse Tracheal Epi Cells (MTEC) cultured at ALI, unstimulated | *In vivo work not used in final analysis* | - AA-induced expression of IL8, CCL20, GM-CSF and TSLP were attenuated in NHBEs CARMA KO vs controls - Predominant GPCRs in mouse AECs include P2Y_2_R, PAR1 and PAR2 - CARMA3 mediates proinflammatory cytokine and chemokine production downstream of multiple GPCRs |
| Causton et al, 2018 | *Alternaria alternata* | CARMA3, ITPR, IL33, Ca^2+^, ILC2, | N/A | NHBEs with CARMA3 KO using shRNA  Cultured at ALI.  Fungal dose: 100ug for 6 hrs | SPC(cre)/CARMA3(F/F), (CARMA3 F) SPC(cre)/CARMA3(+/+), (CARMA3+) 6-8 weeks old, sex matched.  Sensitisation: 100ug of AA (2mg/ml) in 50ul PBS intranasally on Day 1-4.  Challenge only: single dose of 100ug AA  Control: PBS only | - Reduced inflammation and eosinophil and neutrophil BALF counts in mice with reduced AEC CARMA3 expression - Protein levels of IL33 24h after single AA dose were lower in CARMA deficient mice vs controls. CARMA3 KO of NHBEs showed reduced IL33 RNA levels at 6h and reduced protein levels - AA-exposed CARMA3 KO reduced expression of Th2 cytokines and ILC2 numbers and AA-induced Ca^2+^ flux was delayed in CARMA3 KO ECs - CARMA3 was associated with several other intracellular signalling cascades including ITPR3-mediated Ca^2+^ flux - CARMA3 promoted allergic airway inflammation in response to AA and was necessary for IL33 production and immediate release after allergen exposure |
| Chen et al, 2011 | *Penicillium citrinum* protease 13 (Pc13), normal and denatured | Protease, barrier integrity, NRF2, oxidative stress, actin cytoskeleton | N/A | NCI-H441, cultured at monolayer  Fungal dose: 30nM, applied in media. Controls: PBS | Female BALB/c mice age 6-8 weeks  Sensitisation: 25ug/ml Pc13 in PBS 40ul i.t inhalation 10 consecutive  Controls: PBS | - Pc13 exposed mice showed greater AHR, marked GCM, and increased BALF leukocytes and total IgE vs controls - Pc13 exposed mice showed increased collagen deposition and elevated lung hydroxyproline vs controls - Pc13 treatment resulted in time-dependent cleavage of occludin and E-cadherin. ZO-1 was markedly reduced vs control and TEER decreased in a time-dependent manner - 20% differentially expressed proteins were related to oxidoreduction and 8% to protein folding - Pc13 drove Th2 and IgE associated inflammation in the lung and may contribute to tissue damage associated with AAD such as asthma |
| Chiu et al, 2007 | *Penicillium citrinum* protease 13 (Pc13) | PAR1, PAR2, Ca^2+^, protease, IL8, ERK1/2, PLC | N/A | A549 cells, cultured at monolayer  NHBE from human bronchus, cultured at ALI  Fungal dose: A549s – 0-8nM for 0-24h, applied in media  HAECs – 0-2nM for various timepoints, applied apically. | N/A | - Pc13 caused dose-dependent increase in IL8 secretion, mRNA level for IL1B, IL8, IL6 and GM-CSF increased in cells. IL8 secretion was abolished by serine protease inhibitors and PAR1 and PAR2 antibodies - ERK inhibitor and PLC inhibitor also reduced IL8 release - Pc13 increased PAR1 and 2 mRNA and protein levels in cells and induced a rapid increase in Ca^2+^ via PAR1 and PAR2. ERK phosphorylation peaks within 10-15mins of exposure and then declined - Pc13 had serine protease activity that activated PAR1 and PAR2, inducing IL8 release via PLC - Pc13 induced IL8 expression by Ca^2+^-dependent signalling and increased in Ca^2+^ is upstream of ERK 1/2 activation |
| Chung et al, 2007 | *Penicillium chrysogenum*, crude antigen preparation (PCE) | Nerve Growth factor (NGF), Brain Derived Neurotrophic Factor (BDNF), Neurotrophin 3 (NT3), Neurotrophin 4 (NT4) | N/A | N/A | 50 day old BALB/c mice  Sensitisation: 10, 20, 50, or 70 ug PCE in 50ul HBSS, o.p involuntary aspiration 4/ 4 week.  Controls: 3/ HBSS and final single dose of PCE or HBSS only | - NGF levels were increased in BALF and serum of mice exposed to 50ug and 70ug PCE at D0 and D1 vs controls. Single exposure did not increase NGF - NT4 levels increased in BALF and serum of mice exposed to 50ug and 70ug PCE vs controls. Single exposure did not increase NT4 - NT3 levels increased in BALF of mice exposed to 50ug and 70ug PCE vs controls. Single exposure did not increase NT3 - There was no change in BDNF levels between PCE-treated mice vs control |
| Daines et al, 2020 | *Alternaria alternata*, lyophilised cake of filtrate | PAR2, EGFR, IL6, IL8, | N/A | Primary HBEs, cultured at monolayer and ALI  Primary MTEs cultured at monolayer  Fungal dose: 0, 7.5 or 25ug/ml for 6 or 16hrs | C57BL/6 WT and C57BL/6 PAR2-KO, 5 weeks old.  Sensitisation: 10ug AA filtrate in HBSS every other day 3 times, i.n. instillation  Control: HBSS | - Double dose of AA was required for response in ALI cultures vs monolayers - AA induced dose-dependent AHR and increased eosinophils and neutrophils in BALF vs control, responses not affected by PAR2 KO - Cytokines upregulated in response to AA included: G-CSF, IL1α, IL6, IL5, IL9 and IL17. PAR2 KO did not affect cytokine levels in BAL - EGFR-specific inhibitor repressed AA-induced IL6 and IL8 in HBEs - PAR2 did not appear to play a role in AA-induced epithelial cytokine expression but was at least partially mediated by EGFR pathway |
| De Luca et al, 2017 | *Aspergillus fumigatus* viable resting conidia and culture filtrate | IL17A, IL17F, IL17RA, IL17RC, IL33 | N/A | Primary HBEs cultured at ALI, Mouse lung epithelial cells cultured at monolayer  Fungal dose: 1:1 ratio of conidia to cells | C57BL/6, IL17ra^-/-^, IL17a^-/-,^ IL17f^-/-^, 8-10 weeks old.  Sensitisation: i.p and s.c. injection of 100ug AF culture filtrate extract in Freunds Adjuvant, plus 2 x 20ug culture filtrate extract in PBS i.n. instillations 2 weeks apart.  Challenge: 2 x 10^7 conidia i.n. delivery. | - Increased GCM and peri-bronchial collagen deposition as well as increased BALF eosinophils and total IgE in AF-sensitised *IL17ra^-/-‑^* mice vs AF-sensitised WT and *IL17f^-/-^* - Silencing of *IL17rc* resulted in lower inflammatory pathology in all groups - Expression of Th2 cytokines and Th17 cytokines were elevated in AF-sensitised *IL17ra^-/-‑^* mice vs AF-sensitised WT while IL10 was downregulated - IL17F/IL17RC axis dysregulation caused a predisposition to allergic inflammation - *IL17ra^-/-‑^* mice developed type 2 inflammation to AF and IL17RA was required for restraining allergic reactivity to exposure to AF |
| Doherty et al, 2012 | *Alternaria alternata*,  *Candida albicans*  *Aspergillus fumigatus*  extracts | FIZZ1, STAT6, PAR2, IL33, IL5, IL13 | N/A | N/A | C57BL/6, PAR2^-/-‑^, STAT6^-/-^, 6-8 weeks old  Fungal challenge: 100ug in 80ul PBS once, i.n. delivery  Controls: PBS only | - Only mice that received AA developed significant airway eosinophilia vs AF and CA. AA induced eosinophilia in a dose-dependent manner. BALF IL33 and lung IL5 & IL13 increased after AA challenge vs control - *Alternaria* specifically induced eosinophilia vs to other fungal allergens and induced FIZZ1, which persisted for days - FIZZ1 and acute inflammatory events induced by AA were STAT6-dependent but not PAR2-dependent - FIZZ1 had roles in promoting eosinophilia, epithelial changes and peribronchial fibrosis |
| Fritzsching et al, 2016 | *Aspergillus fumigatus* | ENaC, mucin, IL13, IL33, STAT6, IL25, TSLP, ST2, MyD88, MAPK, Periostin | N/A | Primary Mouse Tracheal Epithelial Cells (MTECs) cultured at ALI (not stimulated with fungus) | Scnn1b-Tg mice, Scnn1b -Tg/STAT6 -/- and WT littermates – neonatal (10 days), juvenile (2-3 weeks) & adult (6 weeks)  Sensitisation: 50ul AF (2mg/ml in 0.9%NaCl) every 48hrs, 4 times via i.t. instillation (juveniles and adults only)  Controls: 0.9% NaCl only | - Reduced MCC and AF clearance in *Scnn1b-Tg* mice and increased airway eosinophilia, IL4, IL5, IL13, ILC2 numbers and AHR. *Scnn1b-Tg* mice spontaneously increased IL5, IL13, GCM and AHR vs WT vehicle control. Response to AF exposure was age-dependent - Airway inflammation, GCM, IL13 and eosinophilia were reduced in STAT6 KO *Scnn1b-tg* mice and were further protected after AF exposure - IL33 induced IL13 not dependent on STAT6 but blocked by IL33/ST2 pathway inhibitors - STAT6 signalling mediated the inflammatory response to AF - Mucociliary dysfunction caused reduced clearance of AF |
| Gao et al, 2014 | *Aspergillus fumigatus* conidia | EGF, EGFR, TGFα | N/A | N/A | Wistar Rats, 5-6 weeks of age  A: chronic asthma (CA)  B: CA + 1w AF, C: CA + 3w AF, D: CA + 5w AF, E: CA + 5w saline, F: OVA + saline, G: OVA + saline + 5w AF.  Chronic Asthma: OVA 1mg + 100mg Alum +1ml saline via i.p. and i.n. delivery 3/week  Fungal challenge: 50ul spores (1x 10^5^/ml), 2/ week via i.n. delivery. | - Airway resistance increased in groups C and D vs A, B, E, F, G with increasing levels for longer AF exposure - Group A showed scattered airway epithelial injury and shedding as well as GCM, vs Group E, F, G. Group B, C, D progressively severe epithelial shedding and GCM - TGFα and EGF in BALF increased in groups B, C, D vs A, E, F, G with increasing levels for longer AF exposure - EGFR expression increased in groups B, C, D vs A, E, F, G with increasing levels for longer AF exposure - Inhalation of AF in asthmatic rats aggravated epithelial injury - Chronic exposure upregulated EGFR expression and its ligands and was involved in progressive increase in airway responsiveness |
| Giridhar et al, 2016 | *Aspergillus fumigatus* extract | KIF3A, cilia, ift88, IL13, IL4, mucin | N/A | N/A | Kif3a^Scg+/+^, Kif3a^fl/fl^, Kif3z^ScgΔ/+^, Kif3a^ScgΔ/Δ^ , Ift88^fl/fl^ , Ift88^ScgΔ/Δ^ mice, 6-8 weeks old  Sensitisation: 10ug AF in 50ul saline, i.t instillation 3/week for 3 weeks.  Controls: Saline | - AF exposure increased AHR and inflammatory responses in *Kif3a* null mice. Eosinophils increased in BALF and Th2 cytokines increased in mouse lungs - GCM and mucus hyperproduction observed in mice of all genotypes after AF exposure but were more severe in mice lacking *Kif3a* and *Ift88* - KIF3A had a role in microtubule assembly and in the pathogenesis of fungal induced inflammation and AHR. KIF3A was required for the suppression of Th2 mediated inflammatory responses, mucus hyperproduction and AHR |
| Gordon et al, 2011 | *Aspergillus fumigatus* antigen (culture extract) | Periostin, TGFβ, | N/A | *In vitro results omitted from final analysis* | Pn^-/-^ mice, 12-13 weeks old  Sensitisation: 100ug AF in 40ul saline. 3/week for 3 weeks, i.n inhalation  Controls: saline | - Mucin production and peribronchial fibrosis was observed after AF challenge but there was no difference to WT controls - Periostin deficient mice had increased AHR and serum IgE levels following allergen challenge vs WT - Periostin deficient mice had blunted TGFB responses to allergen, which affected differentiation of Treg cells - Periostin’s role in the airway was to regulate AHR and allergen induced IgE |
| Haczku et al, 2006 | *Aspergillus fumigatus*, culture extract | Surfactant protein D, IL4, IL5, IL13, IFNγ, STAT6 | N/A | N/A | BALB/c, Stat6^-/-^  IL4/IL13^-/-^, SP-D^-/-^ mice, female 8-10 weeks  Sensitisation; 20ug AF + 20mg alum in 100ul PBS + 21% glycerol, i.p injection, D1 & D14.  Challenge: 25ul AF in PBS (12.5mg/ml in PBS + 21% glycerol), i.n. delivery either single challenge or multiple on D0, D7, D14, D21  Controls: glycerol | - Single AF exposure led to marked cellular influx of neutrophils and eosinophils and increased Th2 cytokines. Eosinophilia persisted over 48h after challenge, whilst neutrophils returned to normal. BALF SPD protein levels markedly increased 48h after AF challenge - Sensitised mice that received rIL4 and rIL13 but not rIFNy increased SPD in BALF - SPD production after AF challenge was absent in *Stat6^-/-^* vs WT controls - SPD production increased in response to IL4 and IL13 and was STAT6-dependent |
| Hayes et al, 2018 | *Alternaria alternata*, protease Alt a1 | TLR2, TLR4, MyD88, TIRAP, IL8 | N/A | BEAS-2B cells, NHE, DHBE, cultured at monolayer  Fungal dose: 50ug or 100ug Alt a1 for 24hrs | N/A | - AA a1 induced MCP-1, IL8, CXCL1, CXCL2 and CXCL3 cells. IL8 was peaked at 24h and was dose-dependent - AA a1 primarily induced cytokine secretion (IL8, MCP-1 and CXCL1/2/3) through TLR4 receptor activation. TLR2 receptor was also involved but to a lesser extent |
| Homma et al, 2016 | *Aspergillus fumigatus* extract, High Molecular Weight-AF Low Molecular Weight-AF Heat treated AF extract. | PAR2, STAT1, IFN, IRF3, NF-κB, PTPN11 | N/A | NHBEs (from 3 donors) cultured at ALI and submerged monolayer  Fungal dose: 1:320 wt/vol applied to cells apically or in media. | N/A | - AF extract supressed Poly I:C activated IRF-3 but not NF-KB. AF extract also triggered rapid dephosphorylation of IFNβ induced phospho-STAT1 - Suppressive effect of AF on induction of CXCL10 by TLR3 activators was mediated via activation of PAR2 and in part by PTPN11 activation - PAR2 activation mobilised phosphatases that reduced TLR3 signalling through STAT and IRF |
| Hristova et al, 2016 | *Alternaria alternata* extract | DUOX1, EGFR, IL33, Src, Calpain, P2Y_2_R, H_2_O_2_, ST2, Ca^2+^, ERK1/2 | N/A | NHBE cells, HBE1 cells, MTEC (isolated from WT and DUOX1^-/-^), HNE cells (isolated from healthy or asthma donors)  cultured at monolayer.  Fungal dose: 50ug/ml added to media for 2 or 8 hrs. | C57BL/6J, DUOX1^-/-^ mice, 8-12 weeks  Challenge: o.p. delivery of AA, 50ug in 50ul PBS  Controls: PBS only | - BALF: AA exposure resulted in increased ATP and IL33 in WT vs controls. IL33, IL5, IL13 and IL25 reduced in DUOX1 KO mice. AA induced IL33 reached max levels within 2h but was not associated with cell necrosis - IL33 release was not associated with increased mRNA but showed export of IL33 from nucleus to cytoplasm. IL33 release was dependent on P2Y_2_R and DUOX1 - DUOX1 silencing mediated allergen induced H_2_O_2_ production in cells, decreased IL33 but did not affect ATP levels. DUOX1 and IL33 was dependent on Ca^2+^. - AA exposure induced rapid phosphorylation of Src, EGFR and ERK1/2 and enhanced EGFR phosphorylation. Response absent in DUOX1 KOs - DUOX1 had a specific role in Th2 immune response and was markedly elevated in asthmatic subjects, contributing to exaggerated airway response. IL33 release was mediated by DUOX1 activation of the EGFR pathway. DUOX 1 was also upregulated in asthmatics. |
| Iijima et al, 2021 | *Alternaria alternata* extract | IL33, IL5, IL13, ILC2 | N/A | *In vitro work not used in final analysis* | CCSP-Il33tg mice, non-Tg or single-Tg littermates used as controls  Chronic exposure:  AA (20ug) and OVA (10ug) in 50ul PBS, 2/week, 4 weeks  i.n. instillation.  Acute exposure: 50ug in 50ul PBS, once, i.n instillation  Controls: 50ul PBS only | - CCSP-Il33tg: IL33 overexpression upregulated IL5 expression in BALF but not IL13 at baseline - IL25, TSLP and IL1 cytokines were comparable in transgenic mice and controls exposed to AA - IL33 upregulation increased IL5 production and secretion - Transient increased IL33 in the neonatal period enhanced fungal induced airway innate type 2 immune responses later in life |
| Inoue et al, 2021 | *Alternaria alternata* extract | IL33, ST2, SPRR, TMPRSS2 | N/A | 16HBE14o-, cultured at monolayer  Fungal dose: 0.1ug/ml and 1ug/ml for 24 hours | BALB/c mice, female, 7 weeks old  Sensitisation: 20ul of 50ug AA in PBS, i.n. delivery 2/ week for 6 weeks.  Controls: PBS | - AA exposure caused prominent airway inflammation, increased eosinophils, membrane thickening, increased mucus production and increased airway remodelling - RNA-seq: 403 upregulated genes and 108 downregulated genes after 6 weeks exposure - ST2 expression was increased in AA exposure, IL33 was marginally decreased vs controls - AA downregulated keratinisation in epithelial cells and upregulated genes relating to Ig expression and receptors as well as serine proteases such as Tpsb2 and Tmprss2 and eosinophil migration |
| Jeong et al, 2018 | *Aspergillus fumigatus* crude antigen extract,  *Alternaria alternata* crude antigen extract | NLRP3, PI3Kδ, mtROS, Caspase-1, ASC, IL-1β | Human lung tissue sections,  Patients with:  IPF (n = 4), ABPA (n=6) or healthy (n=3) | NHBE cultured at monolayer, MTEC cultured at monolayer  Fungal dose: 5ug/ml AF antigens for 12 hrs. | C57BL/6 mice, female 8-10 weeks. P110δ KO (deficient for catalytic subunit of PI3Kδ.  AF model: 10ug crude antigen extract + 0.2ml Freunds adjuvant in saline, s.c. + i.p. 20ug AF antigens in saline via i.n. route after 2 weeks. 4 days later, 20ug in saline via i.t. route  AA model: 25ug in saline, i.n. route on D0, 3, 6, and 9. | - NLRP3 was significantly increased in patients with ABPA and to a lesser extent, IPF. NLRP3, caspase 1 and ASC increased and co-localised in AF-exposed mice and in AF-exposed epithelial cells, which was reduced by PI3K-δ inhibitors - IL1β inhibition improved AF induced allergic lung inflammation, including reductions in infiltrating cells - AA-induced allergic inflammation: NLRP3 and PI3K-δ inhibitors reduced increases in eosinophils in BAL, Th2 cytokines (IL4, IL5, IL13) and IL1β in lungs of mice - NLRP3 inflammasome assembly and activation was increased in the lungs of AF- and AA-exposed mice - PI3K-δ played a role in NLRP3 inflammasome assembly and activation in bronchial epithelial cells |
| Kato et al, 2017 | *Alternaria alternata* crude allergen extract | Calprotectin, TSLP, IL25, protease, ATP, TLR4, RAGE | 66 patients with nasal or paranasal sinus disease (classified as ECRS or NECRS).  Inferior turbinate, nasal polyp and uncinate process tissues obtained from patients during endoscopic sinus surgery. | NHBE, pNECs, cultured at monolayer  Fungal dose: 200ug/ml for 24hours | N/A | - Knock-down of RAGE and TLR4 receptors as well as calprotectin proteins significantly inhibited IL25 and TSLP - Calprotectin was involved in AA-induced TSLP and IL25 production as well as the ATP pathway and played an important role in Th2 airway inflammation - Airborne allergens stimulated production and release of calprotectin and protease activity was crucial in this process - Allergen induced calprotectin was increased in cells from CRS patients |
| Khosravi et al, 2018 | *Aspergillus fumigatus*, conidia and heat-inactivated conidia (HI-AF) | TSLP, IL25, IL33  TLR2, TLR4 | N/A | Mouse lung epithelial cell line (TC1 JHU1), cultured at monolayer  Fungal doses:  HI-AF: 3x10^4^, 3x10^6^, 3x10^7^ cells/ ml  Live: 3x10^4^  Conidia in PBS with 0.1% Tween, applied to monolayers in media | N/A | - A concentration-dependent increase in TLR2 and TLR4 expression was observed upon exposure to HI-AF. TLR2 was upregulated by both live AF and HI-AF. TLR4 was upregulated by HI-AF but downregulated by live AF - AF spores stimulated increased secretion of Th2 cytokines IL25, IL33 and TSLP. IL25 and IL33 induced by live AF was higher than that of HI-AF. IL25 induction was higher than other cytokines |
| Kim et al, 2018 | *Aspergillus oryzae*, protease solution (active and inactive) | Protease, UCP2, mtROS, MAPK, ERK, JNK, AP-1, SMAD4, TGF-β | N/A | Primary HBEC, cultured at monolayer  Fungal dose: 10 and 100ng/ml, applied in media | N/A | - JNK and ERK phosphorylation and AP-1 expression increased in cells treated with AO vs controls. ROS production and mtROS increased in a concentration-dependent manner - Reducing ROS resulted in reduced ERK phosphorylation, decreased AP-1 and reduced cytokine secretion - AO proteases regulated expression of inflammatory cytokines and induced mtROS, activating MAPK and AP-1 - mtROS production inhibited UPC2 protein expression via TGF-β and SMAD4 |
| Kouzaki et al, 2009 | *Alternaria alternata* culture filtrate extract | Protease, PAR2, TSLP, IL4, IFNγ | N/A | BEAS2B & NHBE cells cultured at monolayer  Fungal dose: 25-75ug/ml AA in media | N/A | - Release of TSLP in ECs involved PAR2 and serine proteases - TSLP increased in a concentration-dependent manner in response to AA exposure - IFNγ inhibited TSLP release from AA-exposed ECs |
| Kouzaki et al, 2011 | *Alternaria alternata* culture filtrate extract | ATP, IL33, Ca^2+^, P2Y_2_R, P2X_7_R, ST2, MyD88, IL5, IL13 | Human nasal tissues from 12 normal individuals | NHBE cells cultured at monolayer  Fungal dose: 50ug/ml AA in media | BALB/c, C57BL/6, Rag^-/-^, P2X_7_R^-/-^, P2Y_2_R^-/-^, MyD88^-/-^, ST2^-/-^ mice, female, 7-13 weeks old  Challenge: single dose either 50ug or 100ug AA in 50ul PBS, i.n. delivery  Controls: 50ul PBS | - IL5 and IL13 increased in lungs of AA exposed mice vs control but decreased within 24h. After 1h, IL33 increased markedly in BALF, preceding IL5 and IL13 increases - P2 purinergic antagonists and KO suppressed AA induced Ca^2+^ response - ECs actively and rapidly released IL33 in response to fungal allergens - Ca^2+^ and ATP was involved in the translocation of IL33 from the nucleus to the cytoplasm and IL33 release was dependent on P2Y_2_Rs |
| Labram et al, 2019 | *Aspergillus fumigatus*, spores and culture filtrates | ET-1, | N/A | HBECs, cultured at monolayer  Fungal dose: 1x10^5^ spores for 12 or 24hrs or 1ug/ml culture filtrate for 24hrs. | Male C57BL/6J, 8 weeks old  Sensitisation: Spores – 40ul of 4x10^5^ in PBS Tween 80, 9 times over 3 weeks via i.n. delivery. Culture filtrate – 25ul (containing 50ug protein) via i.n. delivery  Controls: PBS tween or PBS only | - Spore inhalation resulted in mild increase in IL4, IL5 and IL6, whereas culture filtrate induced a larger increase - Treatment with ET-1 antagonist reduced extent of peribronchiolar inflammatory infiltration, reduced total BALF cell count and reduced collagen deposition. ET-1 antagonist treatment also reduced AF-induced IL4 and IL6 - ET-1 was upregulated in response to aspergillus exposure, but protein levels remain unchanged in the lung. Culture filtrate resulted in enhanced endothelin protein in BALF - Culture filtrate exposure showed increased airway remodelling in comparison to spore exposure |
| Lee et al, 2020 | *Aspergillus fumigatus* crude antigen extract | PI3Kδ, oxidative stress, Ca^2+^, ATP, VDAC1, IRE1α, GRP78, PDIA6, NLRP3, ASC, Caspase-1, |  | BEAS-2B cells cultured at monolayer  Fungal dose: 100ug/ml AF for 24h, applied in media | C57BL/6 mice, female, 7-8 weeks old  Sensitisation: 10ug crude antigen extract + 0.2ml Freunds adjuvant in saline, s.c. + i.p.  Challenge: 20ug AF antigens in saline via i.n. route after 2 weeks. 4 days later, 20ug in saline via i.t. route  Controls: saline | - AF exposure: Infiltration of eosinophils into bronchioles vs control group. Increased AHR, Th2 cytokines (IL4, IL5, IL13) and PAS staining. ER membrane showed aberrant morphology. Distance between ER and mitochondria was smaller than usual, all reduced by PI3K-δ inhibitors - AF exposure induced increase in protein oxidation, decreased GSH:GSSG ratio, increased HMWC formation and enhanced unfolded protein response. Inhibitors reduced AF-induced changes - Exposure to AF increased mitochondrial ROS and decreased ATP levels while inhibiting COX1 and COX3 activities - PI3K-δ and ER stress contributed to airway inflammation and remodelling in exposure to AF. ER stress marker GRP78 increased by AF exposure and reduced by PI3K-δ inhibitors - AF caused changes in ER membrane fluidity and permeability, close contact between ER and mitochondria, and amplified ROS and Ca^2+^ signalling - AF exposure induced inflammasome activation via VDAC1 and caspase1. VDAC silencing reduced ASC, activated caspase 1 and NLRP3 |
| Lee et al, 2016 | *Aspergillus fumigatus* crude antigen extract | PI3Kδ, mtROS, Akt, GRP78, CHOP, NF-κB | Human lung tissues:  Healthy (n=3), ABPA (n=6) | MTECs cultured at monolayer.  Fungal dose: 5ug/ml AF for 12h, applied in media | C57BL/6 mice, female, 8-10 weeks old  Sensitisation: 10ug crude antigen extract + 0.2ml Freunds adjuvant in saline, s.c. + i.p.  Challenge: 20ug AF antigens in saline via i.n. route after 2 weeks. 4 days later, 20ug in saline via i.t. route  Controls: saline | - AF exposure: Increased BALF cell numbers (especially eosinophils), IgE, Th2 cytokines and AHR. Th2 cytokines were reduced by NF-κB inhibitors - ER stress was increased in lungs of AF-exposed mice - GRP78 was also upregulated in lungs of ABPA patients, and GRP78 and CHOP were upregulated in AF-exposed mice - ER stress may be involved in pathogenesis of AF-related allergic lung disorders, ER stress involved PI3K-δ and mtROS generation |
| Leino et al, 2013 | *Alternaria alternata,*  *Cladosporium herbarum*,  culture extracts | Barrier integrity, IL8, protease, IL33, TSLP | N/A | 16-HBE14o-, polarised cultures on transwells (non-ALI), pHBEcs (from non-asthmatic or severe asthmatics), cultured at ALI  Fungal dose: 25, 50, 100 or 400ug/ml for 24hrs  Controls: untreated | N/A | - Significant dose-dependent decrease in TEER in polarised cultures observed 1h post challenge with AA but recovered quickly at lower doses. Higher dose of AA, TEER remained lower at 24h. CH did not affect TEER. In ALI cultures, AA had no effect on barrier function in healthy donors but severe asthmatics saw a dose-dependent decrease within 3h but recovered by 24h - AA significantly induced release of inflammatory cytokines and increased permeability of polarised epithelial cells potentially due to serine and aspartate protease activity - Differentiated primary cells had a blunted IL8 response but those from asthmatics were more susceptible to barrier weakening |
| Matsuwaki et al, 2012 | *Alternaria alternata, aspergillus versicolor, aspergillus fumigatus, candida albicans, cladosporium herbarum*, *Penicillium spp, Curvularia spp*, culture filtrate extracts | PAR2, GM-CSF, IL6, IL8, Ca^2+^, protease | Isolation of eosinophil, healthy, asthmatic, or allergic rhinitis donors *(exp data does not distinguish between disease type)*.  Fungal dose: 200μg/ml AA | BEAS-2B, Calu-3, cultured at monolayer  Fungal dose: 12.5 – 200ug/ml for up to 24hr for each species. | N/A | - Cells stimulated with AA showed rapid increase in cytosolic free Ca^2+^, observed after 200s and peaked between 400s and 600s - Only AA induced IL6, IL8 and GM-CSF production vs other fungi. AA did not induce eotaxin, eotaxin-2 or RANTES. HI-AA did not induce cytokine production - Aspartate protease inhibitors inhibited cytokine production and Ca^2+^ response in AA exposed epithelial cells - Aspartate proteases but not serine proteases in AA activated PAR2 in epithelial cells - AA but not other fungal extracts activated IL6 production/release. |
| Munitz et al, 2011 | *Aspergillus fumigatus,* culture extract | RELM-α, IL13, IL4, IL13Rα1 | N/A | N/A | Retnla^-/-^, Retnlb^-/-^, Il13ra1^-/-^, BALB/c, C57BL/6  Sensitisation: 100 μg (50 μl) AF, 3/ week for 3 weeks, i.n. delivery  Followed by 2 x i.n. challenges | - AF induced GMC and mucus production as well as production of CCL17 and CCL22 - AF induced upregulation in RELM-α expression but *Il13ra1^-/-^*mice showed no increase - IL13Rα1 critically regulated RELM-α expression after AF challenge - Baseline RELM-α expression was restricted to airway cells. AF challenge increased expression in macrophages - RELM-α did not have a marked role in the production of Th2 cytokines after allergen challenge |
| Murai et al, 2012 | *Alternaria alternata, Aspergillus, Penicillium* extract  (Asp and Pen species unknown) | IL-18, NF-Κb, Caspase-1 | N/A | NHBE cells, A549 cultured at monolayer  Fungal dose: 30ug/ml applied to media | BALB/C, 8 weeks old  Challenge: AA (20ug/ml) in PBS, i.n. administration  Controls: PBS  *Other in vivo models were not included in the final analysis* | - AA increased cell permeability and induced necrosis - AA rapidly induced increases in IL18 levels in cells (26-fold) and BALF (8.6-fold). AA induced much greater IL18 release vs *Aspergillus* and *Penicillium.* IL18 induced Th2 differentiation, which was dependent on NF-κB - AA induced specific IL18 release from AECs vs to other allergens. IL18 release occurred through activation of caspase 1 and induced necrosis |
| Murai et al, 2015 | *Alternaria alternata* extract | IL18, Autophagy | N/A | NHBE cells, A549 cultured at monolayer  Fungal dose: 30ug/ml applied to media | N/A | - AA treatment increased IL18 levels in culture supernatants. Caspase 1 and caspase 8 inhibitors failed to inhibit IL18 release. PI3K inhibitors suppressed AA-induced IL18 - AA treatment augmented formation of autophagosome - AA activated an autophagy-based unconventional secretion pathway in airway epithelial cells and induced the extracellular release of IL18 independent of caspase I and 8 activation |
| Neveu et al, 2009 | *Aspergillus fumigatus* extract | IL6, mucin, IL13, eotaxin, IL17, IL21 | N/A | N/A | C57BL/6, IL6^-/-^, IL21^-/-^ mice  Sensitisation: 5ug in PBS, o.p delivery on D0, 7 & 14.  Serum analysis: i.p. injection with 15ug of AF (and OVA) | - Goblet cell numbers were reduced in *Il6^-/-^* mice and MUC5AC expression was reduced upon AF exposure vs controls - IL6 directly induced IL13 production on CD4^+^ T cells and was essential for mucus production - IL6 and IL21 deficiency resulted in IgE hypersecretion and increased IgE during allergic inflammation. IL6 contributed to IgG1 production - IL6 deficiency impaired IL17 production in induced allergic inflammation - IL6 was rapidly produced in the lung upon allergen exposure and played a key role in dictating immune response against allergens |
| Neveu et al, 2011 | *Aspergillus fumigatus, Candida albicans* extract | IL6, TSLP, MAPK, IL33 | N/A | Mouse lung epithelial cells (from unexposed mice), cultured at ALI for 4 days.  Dose: 1ug/ml AF extract for 24 hours | C57BL/6, MKK3^-/-^, MKK6^-/-^ and Dectin 1^-/-^  Challenge: 1ug/mouse in PBS (and 10ug scleroglucan), o.p delivery | - TSLP, IL33, TNFα or eotaxin were not detected in supernatants of LECs *in vitro* after direct contact with AF allergens - β-glucans predominantly stimulated IL6 production from LECs - Direct interaction between LECs and allergens produced IL6. IL6 gene was constitutively expressed in LECS but not in lung resident immune cells - p38 MAPK was involved in IL6 production by promoting phosphorylation of Mnk1/2 kinases |
| O'Grady et al, 2013 | *Alternaria alternata* extract and antigen preparation | ATP, Ca^2+^, Cytokeratin, protease, PAR2 | N/A | HBE and HBE-A (asthmatic),  16HBE14o-  Cultured at monolayer  Fungal dose: 200ug/ml applied to media (range of doses used for validation initially) | N/A | - Cytokeratin 14, 16 and 6RA expression was higher in primary HBE & HBE-A cells vs 16HBE, however, 16HBE had higher mucin expression - Inhibitors reduced AA induced ATP release by reducing intracellular vesicle movement of ATP - Serine and cysteine protease inhibitors reduced max levels of ATP in HBE cells, but only Leupeptin reduced ATP levels in HBE-A cells - Inhibition of PAR2 receptors did not block effects of AA treatment on Ca^2+^ signalling - HBE-A cells were more sensitive to AA and produced quicker and higher ATP and Ca^2+^ release vs normal cells - ATP release was via PAR2 receptor, but was not fully dependent on it |
| Oguma et al, 2011 | *Aspergillus fumigatus* (fungal extract and culture supernatant), *Aspergillus niger*, *Penicillium notatum*, *Alternaria alternata*, *Candida albicans* fungal extracts | Mucin, TACE, TGF-α, EGFR, protease | N/A | NHBE cells, MTEC (from C57BL/6), cultured at ALI  H292 cells, cultured at monolayer  Fungal dose: 50-1000 PNU/ml for various timepoints | N/A | - AF induced prominent mucus production vs other fungi and control. Weaker mucus production by PN extract vs CA, AA and control - 185 genes specifically upregulated by AF but not PN or AA - EGFR and TGFα inhibitor markedly reduced MUC5AC expression in AF-exposed cells. MMP inhibitor or TACE inhibitor lowered level of MUC5AC mRNA vs control. MUC5AC mRNA was also suppressed by TACE siRNA vs control - The robust serine protease activity of AF was essential for mucin synthesis and expression of MUC5AC in AECs via activation of TACE/TGFα/EGFR pathway |
| Patel et al, 2019 | *Aspergillus fumigatus* fungal extract *Alternaria alternata* fungal extract | solitary chemosensory cells, IL25, GNAT3, T2R8 | N/A | Primary nasal epithelial cells (PNECs) isolated from tissues samples (patients with mycetoma (n=3), AFRS (n=6) and CRSsNP (n=5)), cultured at monolayer.  Fungal dose: 26ug/ml applied in media | N/A | - Frequency of SCCs among epithelial cells was found to be higher in inflamed mucosa vs non-inflamed turbinate tissue for all groups - Frequency of SCCs in AFRS primary cells were elevated in AF and AA vs control - SCCs played a role in detection of fungal antigens causing release of IL25 - SCCs were upregulated in inflamed mucosa in non-invasive allergic fungal conditions when exposed to fungal antigens |
| Ramu et al, 2017 | *Alternaria alternata* allergen extracts | UA, ATP, IL8, IL33, protease | N/A | BEAS-2B, cultured at monolayer  Dose: 10-100ug/ml AA for 1, 6 and 24hrs (HDM used as a comparison) | *In vivo work not included in the final analysis* | - AA caused dose-dependent release of IL8 and ATP - AA showed serine protease activity and IL8 mRNA expression and ATP release was inhibited by serine protease inhibitors but IL8 protein was not - Allergens show distinct differences in their ability to induce DAMPs and alarmins in cells |
| Rivas et al, 2021 | *Alternaria alternata* extract | PAR2, protease, IL6, IL8, RANTES, IP10, VEGF, PDGF | N/A | 16HBE14o- (PAR2 expressing cells and PAR KO), cultured at ALI.  Fungal dose: 30ng - 10ug AA in 100ul HBSS added apically  Controls: HBSS only | N/A | - Impedance assay: AA (at lowest dose) caused initial decrease of cell index followed by an increase similar to protease and PAR2 agonist response. Higher dose led to a secondary loss of cell index in normal cells - AA induced IL6 and IL8 secretion as well as RANTES, VEGF, PDGF and IP10 at 24h, which was reduced by PAR2 antagonist. PAR2 KO cells had reduced cytokine secretion - HBSS control had increased IL8, IP10, RANTES, and TNFα from apical side, suggesting a PAR2-independent pathway exists - AA acted on the PAR2 receptor, but also induced a PAR2-independent pathway |
| Royce et al, 2011 | *Aspergillus fumigatus* antigens | TFF2 | N/A | N/A | Balb/C, TFF2^-/-^ mice  Sensitisation: 100ug AF in 50ul saline via inhalational delivery. 3 treatments per week for 3 weeks  Control: 50ul saline | - AF-exposed *Tff2^-/-^* mice showed marked increase in epithelial thickness and goblet cell numbers vs WT controls - TFF2 attenuated changes in epithelial structure, GCM and subepithelial collagen deposition - TFF2 maintained normal homeostatic structure of airway |
| Samichuwal et al, 2017 | *Alternaria alternata* cellular extract | PGE_2_, IL33, EPR, | N/A | *In vitro work not used in final analysis* | C57BL/6 WT, mPGES-1 KO, 9-11 weeks old  Sensitisation: 12ug in 20ul PBS on D0, 3, 6, 9, i.n. inhalation  Controls: PBS only | - *mPGES* KO mice showed decreased lung inflammation and reduced ILC2s vs WT - *mPGES* KO expressed lower levels of IL33 mRNA vs WT - PGE2 supported the amplification of IL33 expression, which in turn drove downstream IL33-dependent effectors |
| Schiffers et al, 2020 | *Alternaria alternata* culture extract | TRPV1, P2YR2, PAR2, PAR1, EGFR, protease, DUOX1, ATP, IL33, H_2_O_2_ |  | pNECs (healthy and allergic asthma and rhinitis) cultured at ALI, HBE1, MTECs (WT and TRPV1^-/-^ mice)  cells cultured in monolayer  Fungal dose: 3-30ug/ml extract, applied apically | *In vivo work not included in the final analysis* | - mRNA expression of P2Y_2_R and TRPV1 was enhanced in asthmatic NECs vs healthy controls. PAR1 and PAR2 were also increased - AA induced IL33 release suppressed by TRPV1 but not TRPV4. P2Y_2_R and TRPV1 silencing reduced IL33 release - AA exposure produced extracellular H_2_O_2_ (DUOX1 activation), which was supressed by P2Y_2_R inhibitor - PAR2 and TRPV1 activation evoked rapid ATP release. Inhibition of TRPV1 strongly attenuated activation of EGFR - TRPV1/4 induced IL33 secretion requires P2Y_2_R-dependent DUOX1 activation - AA induced DUOX1 activation, EGFR activation and IL33 secretion involve TRPV1. TRPV1 critically contributed to ATP-dependent signalling with P2Y_2_R |
| Schmit et al, 2020 | *Aspergillus fumigatus* spores and culture extract | IL6, TGF-β, STAT3, mucin | N/A | N/A | C57BL/6, IL6^-/-^ mice, 6-8 weeks old  Sensitisation: 10μg AF extract in 100μl PBS and 100μl Alum, s.c.. and o.p. injection  Challenge: 20μg AF extract in 20μl PBS, i.n. delivery, 1 week intervals for 4 weeks, followed by live AF spore aerosol. | - AF exposure caused broncho-interstitial infiltration of immune cells and GCM with higher mucus production and higher levels of eosinophils - IL6 deficiency correlated with decrease goblet cell numbers and mucin production, and decreased MUC5AC expression - IL6 deficiency drove increased eosinophil recruitment, lung pathology and collagen deposition in asthmatic mice - IL6 deficient eosinophils dysregulated lung inflammation by upregulating TGFβ and STAT3 phosphorylation, compromising barrier integrity |
| Shibata et al, 2014 | *Aspergillus fumigatus* antigens and conidia | Gas6, FIZZ1, IL13, Mucin, IL4, Axl | Plasma samples obtained from asthma patients (normal = 8, mild = 15, moderate = 25, severe = 26) | *In vitro work not included in final analysis* | C57BL/6, Gas6^-/-^ mice, 6-8 weeks of age.  Sensitisation: AF antigens in incomplete Freund’s adjuvant, i.p. and s.c. injection.  Challenge: 3 x AF antigen challenge, i.n. delivery followed by 5.0 × 10^6^ spores suspended in 30 μl 0.1% Tween-80, i.t. route | - Gas6^-/-^ mice had suppressed AHR, decreased peribronchial and BAL inflammatory cells, reduced PAS staining and serum IgE - Exogenous Gas6 treatment to AF exposed mice led to increased peribronchial inflammation, increased BAL cells, increased serum IgE, and increased PAS staining - Gas6 was elevated in asthmatic populations regardless of disease severity - Gas6 drove airway inflammation and was involved in airway remodelling |
| Shin et al, 2019 | *AlternarIa alternata* culture extract | Barrier integrity, protease, ROS, | N/A | Primary NECs, cultured at ALI  Dose: 10- 100μg/ml applied apical | N/A | - TEER decreased in a concentration-dependent manner when exposed to AA but returned to baseline by 24h, except at highest dose - AA strongly induced intracellular ROS in cells, but effect was reduced with serine protease inhibitor and heat-inactivation - AA affected TJ molecule but not AJ molecule expression - AA-induced barrier dysfunctions were associated with serine proteases and intracellular ROS |
| Shin et al, 2020 | *Alternaria alternata, Aspergillus fumigatus* culture filtrate | IL25, IL33, TSLP, IL6, NF-κB, AP-1, MAPK, c-Jun, | 30 nasal polyps obtained from 14 ENP, 16 NENP. 8 healthy controls. | Primary NECs, cultured at monolayer  Dose: AA 100 μg/ml, AF 50μg/ml, various timepoints | N/A | - ENP patients had olfactory dysfunction and tissue eosinophilia vs NENP patients. IL25, IL33 and TSLP levels in ENP patients were higher vs NENP and controls - AA stimulated IL33 and TSLP production but not IL25 for 24h and 48h. IL25 was not increased by allergens - AA enhanced IL33 and TSLP and IL6 but AF only increased IL6 production. AA enhanced phosphorylated NF-κB and c-Jun expression, but AF did not. IL33 and TSLP expression was regulated by NF-κB, AP-1 and MAPK |
| Srisomboon et al, 2020 | *Alternaria alternata* extract | VDAC1, ATP, Cav-1, IL33, Ca^2+^, BNIP3, | N/A | HBE cells (immortalised), cultured at monolayer  Fungal dose: 100ug/ml applied in media | N/A | - AA-induced IL33 release decreased by inhibition of VDAC1 in a concentration-dependent manner - Inhibitors and silencing of VDAC1 reduced AA-induced release of ATP and Ca^2+^. VDAC1 was involved in ATP release in epithelial cells exposed to AA - Reduced cholesterol inhibited initial ATP release. Cholesterol played a role in ATP release and Ca^2+^ uptake in AECs exposed to AA - Plasma membrane localisation of VDAC1 was dependent on cholesterol |
| Tai et al, 2006 | *Penicillium chrysogenum*, protease Pen ch 13 (P13) | PGE2, IL8, TGFβ, protease, COX-2, barrier integrity | N/A | A549 cells, 16HBE14o-  NHBE, cultured at  monolayer  Fungal dose: various concentrations between 0.01 – 1ug/ml applied to media over 16hrs | N/A | - P13 induced production of PGE2, IL8 and TGF-β in AECs - Mediator release and COX-2 were inhibited by a serine protease inhibitorP13 degraded occluding, which can disrupt the epithelial barrier |
| Uchida et al, 2017 | *Alternaria alternata, Aspergillus fumigatus* extracts | IL33, ROS, ATP, Ca^2+^, Nrf2 | N/A | NHBE cells, cultured at monolayers  Fungal dose:  AA: 100-200ug/ml,  AF: 100-400 ug/ml | BALB/c, ST2^-/-^ mice  Acute model: 20, 50 or 100ug AA, i.n. delivery  Chronic model: 10ug AA 3/week for 2 weeks, i.n. delivery | - AA-exposed mice treated with CDDO-Me had reduced airway eosinophilia, IgE expression, peribronchial inflammation and GCM. IL5 and IL13 expression were also reduced. CDDO-Me reduced or abolished IL33 release after AA exposure - Full length IL33 remained in tissues, short IL33 was secreted - IL33 release was associated with increased ROS, ATP release and Ca^2+^ signalling - Activation of Nrf2 pathway reduced allergic pathology, type 2 cytokines and IL33 release |
| Wiesner et al, 2020 | *Aspergillus* fumigatus rALP1 and culture filtrate | TRPV4, protease, IL5, ILC2, IL33, barrier integrity | Paraffin embedded bronchial biopsies, asthma (n=3) and healthy (n=5). | Primary BECs, cultured at ALI  Dose: various doses for 1hr | C57BL/6 mice, 8-16 weeks old  Sensitisation: 25ug rALP1 in 25ul PBS, or 25ul culture filtrate, inhalational delivery on D1,2,7,14. | - Alp1 caused allergic pathology in lung including leukocyte infiltration and GCM - TSLP did not increase from baseline after Alp1 exposure but IL25 and IL33 increased post 24h - Alp1 did not cleave PAR1 or 2. Alp1 cleaves C3 to form C3a like molecule but does not affect allergic response - Elevated TRPV4 expression in asthmatics exacerbated the inflammatory response to proteases - Epithelial damage through barrier integrity sensed via TRPV4 prompting Ca^2+^ flux- and calcineurin-dependent inflammation |
| Wong et al, 2020 | *Aspergillus fumigatus* crude extract | RGS4, PGE2, Ca^2+^, PAR2, protease | Mild/moderate (n=19), severe (n=24) asthma lung tissues and controls (n=10) | N/A | Balb/c, C57BL/6, Rgs4^-/-^, ptges1^-/-^, 6-12 weeks old  Sensitisation: 20ug AF with 20mg Alum, i.p. injection on D0 and D14  Challenge:25ug AF i.n. delivery  Controls: PBS | - RGS4 immunoreactivity was more extensive in asthmatic vs normal bronchial epithelium and increased proportionally with disease severity - MUC5AC expression correlated with RGS4 expression - PGE2 was detected at higher levels in BALF from AF-exposed mice vs controls and levels were even higher when RSG4 was knocked down. AF challenge caused increased PGE expression through PAR2 activation - PAR2 mediated bronchodilation through an RGS4-regulated pathway in mice. RGS4 inhibited PGE2 secretion by limiting GPCR-induced G protein activation upstream of PGE2 biosynthesis |
| Wu et al, 2020 | *Aspergillus fumigatus* extract | Mucin, EGFR, protease, Ras/Raf1/ERK, ROS, Ca^2+^, PAR2, | N/A | NCI-H292, cultured at monolayer  Fungal dose: 7.5ug/ml, added via media for various timepoints | N/A | - AF dose-dependently induced MUC5AC expression. Increase occurred as early as 6h after treatment. MUC5B also dose-dependently increased but less than MUC5AC - AF-induced mucin expression repressed by EGFR tyrosine kinase inhibitors, EGFR blocking antibody, and serine protease inhibitors. PAR2 neutralising antibody did not block mucin gene expression - AF-induced expression of MUC5AC and MUC5B in ECs independent of PAR2. EGFR activity was required, but not sufficient, to induce mucins. Mucins induced by activation of ERK pathway. Blocking Ca^2+^ but not ROS prevented mucin induction |
| Zaidman et al, 2017 | *Alternaria alternata* culture extract | CFTR, CaCC, ROS, Barrier integrity, Ca^2+^, ATP, H_2_O_2_, protease | N/A | 16HBE14o-, cultured at monolayer  Fungal dose: 50-200 ug/ml for various times | N/A | - Epithelial barrier integrity remained intact despite reduced TEER values with AA exposure - AA exposure stimulated transepithelial anion secretion through the CTFR and CaCC channels. AA-induced secretion was blocked by channel inhibitors. ATP release from epithelium activated Ca^2+^ uptake through CaCC mediated pathway in apical membrane - Increase in Ca^2+^ stimulated Cl^-^ efflux - AA exposure stimulated ATP secretion as early as 5 min and increased up to 20mins - Increase in oxidative stress contributed to decrease in epithelial resistance |

AA: *Alternaria alternata*, AAD: allergic airways disease, ABPA: allergic bronchopulmonary aspergillosis, AEC: airway epithelial cells, AF: *Aspergillus fumigatus,* AFRS: Allergic fungal rhinosinusitis, AHR: airway hyperresponsiveness, AJ: adherens junctions, ALI: air-liquid interface, AO: *Aspergillus oryzae,* AP-1: activator protein 1, ASC: apoptosis-associated speck-like protein containing a CARD, ATP: adenosine triphosphate, BALF: bronchoalveolar lavage fluid, BDNF: brain-derived neurotrophic factor, CA: *Candida albicans,* Ca^2+^: calcium, CaCC: calcium-dependent chloride channel, cAMP: cyclic adenosine monophosphate, CARMA: CARD-containing membrane associated guanylate kinase protein, Cav-1: caveolin-1, CCL: chemokine (C-C motif) ligand, CCSP: club cell secretory protein, CDDO-Me: bardoxolone methyl, CFTR: cystic fibrosis transmembrane conductance regulator, CH: *Cladosporium herbarum,* CHOP: C/EBP homologous protein, Cl^-^: chloride, COX: cytochrome c oxidase, cPLA2γ: cytosolic phospholipase Α2γ, CRS: chronic rhinosinusitis, CXCL: chemokine (C-X-C motif) ligand, CysLTR: cysteinyl leukotriene receptor, DAMP: danger-associated molecular pattern, DAP12: DNAX-activation protein of 12kDa, DUOX: dual oxidase, EC: epithelial cells, EGF: epidermal growth factor, EGFR: epidermal growth factor receptor, ENaC: epithelial sodium channel, ENP: eosinophilic nasal polyps, ER: endoplasmic reticulum, ERK: extracellular signal-regulated kinase, ET: endothelin, FIZZ1: found inflammatory zone 1, Gas: growth arrest – specific, GCM: goblet cell metaplasia, G-CSF: granulocyte colony-stimulating factor, GM-CSF: granulocyte-macrophage colony-stimulating factor, GPCR: G-protein coupled receptor, GRP: glucose regulated protein, GSH: glutathione, GSSG: glutathione disulfide, HBE: human bronchial epithelial cells, HBE-A: human bronchial epithelial cells – asthmatic, HBSS: hanks balanced salt solution, HI: heat-inactivated, HMWC: high molecular weight compound, Ig: Immunoglobulin, IFN: interferon, IL: interleukin, IP10: interferon gamma-induced protein 10, IPF: idiopathic pulmonary fibrosis, IRF: interferon regulatory factors, ITPR: inositol 1,4,5-trisphosphate receptors, JNK: c-jun N-terminal kinase, KIF3A: kinesin-like protein 3A, KO: knock-out, LEC: lung epithelial cell, LTC_4_: leukotriene C, LTE4: leukotriene E4, MAPK: mitogen-activated protein kinases, MCC: mucociliary clearance, MCP-1: monocyte Chemoattractant Protein-1, MIP: macrophage inflammatory protein, MMP: matrix metalloprotease, Mnk: MAPK interacting protein kinases, mt: mitochondrial, NEC: nasal epithelial cellsNENP: non-eosinophilic nasal polyps, NF-κB: nuclear factor kappa-light-chain-enhancer of activated B cells, NGF: nerve growth factor, NHBE: normal human bronchial epithelial, NLRP: nucleotide-binding oligomerization domain, leucine rich repeat and pyrin domain containing, Nrf2: nuclear factor erythroid 2–related factor 2, NT: neurotrophin, P13: *Penicillium chrysogenum* protease 13, P2YR2: P2 purinergic receptor 2, PAR: protease activated receptor, PAS: periodic acid schiff, Pc13: *Penicillium citrinum* protease 13, PCE: *Penicillium chrysogenum* crude antigen preparation, PDGF: platelet-derived growth factor, PGE: prostaglandin E, PI3K: phosphoinositide 3-kinase, PLC: phospholipase C, PN: *Penicillium notatum,* PTPN: tyrosine-protein phosphatase non-receptor, RAGE: receptor for advanced glycation endproducts, RANTES: regulated on activation, normal T expressed and secreted, RELM: resistin-like molecules, RGS4: regulator of G protein signalling 4, ROS: reactive oxygen species, SCC: solitary chemosensory cells, SMAD: small mothers against decapentaplegic, SPD: surfactant protein D, Src: proto-oncogene tyrosine-protein kinase Src, ST2: suppression of tumorigenicity 2, STAT: signal transducer and activator of transcription, TACE: tumor necrosis factor-alpha converting enzyme, TEER: trans epithelial electrical resistance, TFF: trefoil factor, TGF: transforming growth factor, Th: T helper, TJ: tight junctions, TLR: toll like receptor, Tmprss: transmembrane protease serine, TNF: tumour necrosis factor, Tpsb2: tryptase beta-2, TREM: triggering receptor expressed on myeloid cells, TRPV: transient receptor potential vanilloid, TSLP: thymic stromal lymphopoietin, UCP: uncoupling protein, VDAC: voltage-dependent anion channel, VEGF: vascular endothelial growth factors, WT: wild-type, ZO: zonal occludens.

**Supplementary Information 4:** Frequency of potential target mechanisms (individual genes or pathways) identified in studies.

| Target | No of studies | Target | No of studies | Target | No of studies |
| --- | --- | --- | --- | --- | --- |
| Protease | 19 | ENaC | 2 | IL17R | 1 |
| IL33 | 18 | FIZZ1 | 2 | IL1β | 1 |
| Ca^2+^ | 13 | GM-CSF | 2 | IL21 | 1 |
| PAR2 | 13 | GRP78 | 2 | IP10 | 1 |
| IL13 | 10 | IL17 | 2 | IRE1a | 1 |
| ATP | 9 | IL18 | 2 | IRF3 | 1 |
| IL8 | 9 | NLRP3 | 2 | JNK | 1 |
| ROS | 9 | NRF2 | 2 | KIF3A | 1 |
| Mucin | 8 | PAR1 | 2 | LTE4 | 1 |
| TSLP | 8 | Periostin | 2 | MMP9 | 1 |
| IL4 | 7 | SPD | 2 | NGF | 1 |
| IL6 | 7 | TGFα | 2 | NT3 | 1 |
| Barrier integrity | 6 | TRPV1/4 | 2 | NT4 | 1 |
| EGFR | 6 | VDAC1 | 2 | P2X_7_R | 1 |
| IL25 | 5 | ITPR | 2 | p65 | 1 |
| IL5 | 5 | Actin cytoskeleton | 1 | PDGF | 1 |
| STAT pathway | 5 | Akt | 1 | PDIA6 | 1 |
| ERK1/2 | 4 | ATF-2 | 1 | PLC | 1 |
| NF-κB | 4 | Axl | 1 | PTPN11 | 1 |
| P2Y_2_R | 4 | BDNF | 1 | RAF1 | 1 |
| ST2 | 4 | BNIP3 | 1 | RAGE | 1 |
| TGFβ | 4 | Calpain | 1 | RANTES | 1 |
| IFNγ | 4 | Calprotectin | 1 | RAS | 1 |
| Caspase-1 | 3 | CCL17 | 1 | RELMa | 1 |
| H_2_O_2_ | 3 | CCL20 | 1 | RGS4 | 1 |
| ILC2 | 3 | CHOP | 1 | SCC | 1 |
| MAPK | 3 | Cilia | 1 | SMAD4 | 1 |
| MyD88 | 3 | COX2 | 1 | SPRR | 1 |
| PGE_2_ | 3 | cPLA2y | 1 | Src | 1 |
| PI3Kδ | 3 | Cytokeratin | 1 | T2R8 | 1 |
| TLR2 | 3 | DAP12 | 1 | TACE | 1 |
| TLR4 | 3 | EGF | 1 | TFF2 | 1 |
| AP-1 | 2 | Eotaxin | 1 | TIRAP | 1 |
| ASC | 2 | EPR | 1 | TMPRSS2 | 1 |
| CaCC | 2 | Et-1 | 1 | TREM-1 | 1 |
| CARMA3 | 2 | GAS6 | 1 | UA | 1 |
| Caveolin-1 | 2 | GNAT2 | 1 | UCP2 | 1 |
| CFTR | 2 | GPR99 | 1 | USF1 | 1 |
| c-Jun | 2 | IFT88 | 1 | USF2 | 1 |
| DUOX1 | 2 | IL13Ra1 | 1 | VEGF | 1 |

Akt : protein kinase B, AP-1: activator protein 1, ASC: apoptosis-associated speck-like protein containing a CARD, ATF: activating transcription factor, ATP: adenosine triphosphate, BDNF: brain-derived neurotrophic factor, BNIP3: Bcl-2 interacting protein 3, Ca^2+^: calcium, CaCC: calcium-dependent chloride channel, CARMA: CARD-containing membrane associated guanylate kinase protein, CCL: chemokine (C-C motif) ligand, CFTR: cystic fibrosis transmembrane conductance regulator, CHOP: C/EBP homologous protein, COX: cyclooxygenase-2, cPLA2γ: cytosolic phospholipase Α2γ, CysLTR: cysteinyl leukotriene receptor, DAP12: DNAX-activation protein of 12kDa, DUOX: dual oxidase, EGF: epidermal growth factor, EGFR: epidermal growth factor receptor, ENaC: epithelial sodium channel, ERK: extracellular signal-regulated kinase, ET: endothelin, FIZZ1: found inflammatory zone 1, Gas: growth arrest – specific, G-CSF: granulocyte colony-stimulating factor, GM-CSF: granulocyte-macrophage colony-stimulating factor, GNAT2: G Protein Subunit Alpha Transducin 2, GRP: glucose regulated protein, H_2_O_2_: hydrogen peroxide IFN: interferon, IFT88: intraflagellar Transport 88, IL: interleukin, ILC: Innate lymphoid cells, IP10: interferon gamma-induced protein 10, IRF: interferon regulatory factors, ITPR: inositol 1,4,5-trisphosphate receptors, JNK: c-jun N-terminal kinase, KIF3A: kinesin-like protein 3A, LTC_4_: leukotriene C, LTE4: leukotriene E4, MAPK: mitogen-activated protein kinases, MCP-1: monocyte Chemoattractant Protein-1, MIP: macrophage inflammatory protein, MMP: matrix metalloprotease, Mnk: MAPK interacting protein kinases, MyD88: Myeloid differentiation primary response 88, NF-κB: nuclear factor kappa-light-chain-enhancer of activated B cells, NGF: nerve growth factor, NLRP: nucleotide-binding oligomerization domain, leucine rich repeat and pyrin domain containing, Nrf2: nuclear factor erythroid 2–related factor 2, NT: neurotrophin, P2X7R: P2X purinoceptor 7 receptor, P2YR2: P2 purinergic receptor 2, PAR: protease activated receptor, PDGF: platelet-derived growth factor, PDIA: protein disulfide isomerase family A, PGE: prostaglandin E, PI3K: phosphoinositide 3-kinase, PLC: phospholipase C, PTPN: tyrosine-protein phosphatase non-receptor, RAGE: receptor for advanced glycation endproducts, RANTES: regulated on activation, normal T expressed and secreted, RELM: resistin-like molecules, RGS4: regulator of G protein signalling 4, ROS: reactive oxygen species, SCC: solitary chemosensory cells, SMAD: small mothers against decapentaplegic, SPD: surfactant protein D, Src: proto-oncogene tyrosine-protein kinase Src, ST2: suppression of tumorigenicity 2, STAT: signal transducer and activator of transcription, T2R8: Taste receptor type 2, TACE: tumor necrosis factor-alpha converting enzyme, TFF: trefoil factor, TGF: transforming growth factor, Th: T helper, TIRAP: Toll/interleukin-1 receptor domain-containing adapter protein, TLR: toll like receptor, Tmprss: transmembrane protease serine, TNF: tumour necrosis factor, Tpsb2: tryptase beta-2, TREM: triggering receptor expressed on myeloid cells, TRPV: transient receptor potential vanilloid, TSLP: thymic stromal lymphopoietin, UA: uric acid, UCP: uncoupling protein, USF: upstream stimulatory factor, VDAC: voltage-dependent anion channel, VEGF: vascular endothelial growth factors, ZO: zonal occludens

**Supplementary Information 5:** Individual quality scores assigned to each study. Scores were assigned as described in Supplementary Information 2. Overall average was calculated by dividing the total score by the number of domains and subdomains which contained a score. For example an *in-vitro* or *in vivo* only study would have the overall score divided by 6 but a study which contained both *in vivo* and *in vitro* work would be divided by 9. The overall average was then assigned a quality score (very low, low, moderate, high) based on where the average score fell within set bands: very low (0 - 1), low (1.1 – 2), moderate (2.1 – 3), high (3.1 – 4).

| **Author** | D1 | | D2 | | D3 | D4 | D5 | D6 (in vitro) | D7 (in vivo) | Total score | Overall average | Score |
| --- | --- | --- | --- | --- | --- | --- | --- | --- | --- | --- | --- | --- |
|  | in vitro | in vivo | in vitro | in vivo |  |  |  |  |  |  |  |  |
| Anagnostopoulou et al, 2010 |  | 3 |  | 3 | 3 | 1 | 3 |  | 3 | 16 | 2.7 | MODERATE |
| Babiceanu et al, 2013 | 2 |  | 2 |  | 1 | 1 | 4 | 3 |  | 13 | 2.2 | MODERATE |
| Bains et al, 2012 | 1 | 2 | 3 | 2 | 1 | 2 | 1 | 1 | 4 | 17 | 1.9 | LOW |
| Bankova et al, 2016 |  | 3 |  | 3 | 2 | 3 | 1 |  | 3 | 15 | 2.5 | MODERATE |
| Bickford et al, 2012 | 2 | 2 | 2 | 4 | 2 | 3 | 2 | 1 | 4 | 22 | 2.4 | MODERATE |
| Boitano et al, 2011 | 2 | 4 | 2 | 1 | 3 | 1 | 1 | 1 | 3 | 18 | 2 | LOW |
| Brandt et al, 2008 | 1 | 3 | 1 | 2 | 2 | 2 | 3 | 1 | 3 | 18 | 2 | LOW |
| Buckland et al, 2011 |  | 2 |  | 2 | 2 | 3 | 3 |  | 2 | 14 | 2 | MODERATE |
| Causton et al, 2015 | 3 |  | 3 |  | 2 | 4 | 4 | 1 |  | 17 | 2.8 | MODERATE |
| Causton et al, 2018 | 3 | 3 | 3 | 2 | 3 | 4 | 4 | 1 | 3 | 26 | 2.9 | MODERATE |
| Chen et al, 2011 | 2 | 4 | 1 | 3 | 1 | 2 | 1 | 1 | 3 | 18 | 2.0 | LOW |
| Chiu et al, 2007 | 4 |  | 3 |  | 4 | 4 | 2 | 1 |  | 18 | 3.0 | MODERATE |
| Chung et al, 2007 |  | 4 |  | 3 | 1 | 2 | 1 |  | 1 | 12 | 2 | LOW |
| Daines et al, 2020 | 4 | 3 | 4 | 3 | 4 | 4 | 3 | 1 | 3 | 29 | 3.2 | HIGH |
| De Luca et al, 2017 | 3 | 2 | 4 | 4 | 3 | 4 | 1 | 2 | 2 | 25 | 2.8 | MODERATE |
| Doherty et al, 2012 |  | 3 |  | 4 | 3 | 4 | 4 |  | 3 | 21 | 3.5 | HIGH |
| Fritzsching et al, 2016 | 1 | 3 | 3 | 3 | 2 | 4 | 4 | 2 | 3 | 25 | 2.8 | MODERATE |
| Gao et al, 2014 |  | 3 |  | 2 | 1 | 2 | 1 |  | 2 | 11 | 1.8 | LOW |
| Giridhar et al, 2016 |  | 3 |  | 4 | 2 | 3 | 3 |  | 3 | 18 | 3.0 | MODERATE |
| Gordon et al, 2011 |  | 4 |  | 3 | 3 | 4 | 3 |  | 3 | 20 | 3.3 | HIGH |
| Hackzu et al, 2006 |  | 2 |  | 4 | 3 | 4 | 4 |  | 2 | 19 | 3.2 | HIGH |
| Hayes et al, 2018 | 2 |  | 3 |  | 3 | 2 | 3 | 1 |  | 14 | 2.3 | MODERATE |
| Homma et al, 2016 | 3 |  | 4 |  | 4 | 4 | 2 | 2 |  | 19 | 3.2 | HIGH |
| Hristova et al, 2017 | 2 | 3 | 3 | 3 | 4 | 4 | 4 | 2 | 1 | 26 | 2.9 | MODERATE |
| Iijima et al,2021 |  | 3 |  | 3 | 1 | 2 | 4 |  | 2 | 15 | 2.5 | MODERATE |
| Inoue et al, 2021 | 2 | 3 | 2 | 3 | 1 | 1 | 3 | 1 | 3 | 19 | 2.1 | MODERATE |
| Jeong et al, 2018 | 2 | 3 | 3 | 3 | 4 | 2 | 4 | 3 | 3 | 27 | 3.0 | MODERATE |
| Kato et al, 2017 | 2 |  | 3 |  | 4 | 4 | 3 | 3 |  | 19 | 3.2 | HIGH |
| Khosravi et al, 2018 | 2 |  | 2 |  | 1 | 3 | 4 | 3 |  | 15 | 2.5 | MODERATE |
| Kim et al, 2018 | 2 |  | 3 |  | 2 | 4 | 2 | 1 |  | 14 | 2.3 | MODERATE |
| Kouzaki et al, 2009 | 2 |  | 3 |  | 4 | 3 | 3 | 2 |  | 17 | 2.8 | MODERATE |
| Kouzaki et al, 2011 | 2 | 3 | 3 | 2 | 3 | 4 | 4 | 4 | 1 | 26 | 2.9 | MODERATE |
| Labram et al, 2019 | 2 | 4 | 3 | 3 | 2 | 4 | 3 | 1 | 3 | 25 | 2.8 | MODERATE |
| Lee et al, 2016 | 2 | 2 | 4 | 2 | 2 | 4 | 4 | 1 | 2 | 23 | 2.6 | MODERATE |
| Lee et al, 2020 | 2 | 2 | 2 | 3 | 2 | 2 | 4 | 1 | 2 | 20 | 2.2 | MODERATE |
| Leino et al, 2013 | 4 |  | 4 |  | 2 | 2 | 3 | 3 |  | 18 | 3 | MODERATE |
| Matsuwaki et al, 2012 | 2 |  | 2 |  | 3 | 2 | 2 | 3 |  | 14 | 2.3 | MODERATE |
| Munitz et al, 2012 |  | 3 |  | 2 | 2 | 4 | 4 |  | 4 | 19 | 3.2 | HIGH |
| Murai et al, 2012 | 2 | 3 | 3 | 4 | 2 | 2 | 4 | 4 | 3 | 27 | 3.0 | MODERATE |
| Murai et al, 2015 | 2 |  | 3 |  | 3 | 2 | 2 | 3 |  | 15 | 2.5 | MODERATE |
| Neveu et al, 2009 |  | 3 |  | 2 | 2 | 3 | 4 |  | 3 | 17 | 2.8 | MODERATE |
| Neveu et al, 2011 | 3 | 3 | 4 | 4 | 2 | 4 | 3 | 1 | 2 | 26 | 2.9 | MODERATE |
| O'Grady et al, 2013 | 2 |  | 3 |  | 4 | 4 | 1 | 1 |  | 15 | 2.5 | MODERATE |
| Oguma et al, 2021 | 4 |  | 3 |  | 2 | 3 | 1 | 1 |  | 14 | 2.3 | MODERATE |
| Patel et al , 2019 | 2 |  | 4 |  | 1 | 2 | 3 | 3 |  | 15 | 2.5 | MODERATE |
| Ramu et al, 2017 | 2 |  | 2 |  | 2 | 3 | 3 | 1 |  | 13 | 2.2 | MODERATE |
| Rivas et al, 2021 | 4 |  | 3 |  | 3 | 2 | 2 | 1 |  | 15 | 2.5 | MODERATE |
| Royce et al, 2011 |  | 3 |  | 3 | 2 | 2 | 1 |  | 3 | 14 | 2.3 | MODERATE |
| Samichuwal et al, 2017 |  | 4 |  | 2 | 2 | 4 | 3 |  | 3 | 18 | 3.0 | MODERATE |
| Schiffers et al, 2020 | 4 |  | 3 |  | 4 | 4 | 4 | 1 |  | 20 | 3.3 | HIGH |
| Schmit et al, 2020 |  | 2 |  | 4 | 3 | 4 | 3 |  | 2 | 18 | 3.0 | MODERATE |
| Shibata et al, 2014 | 2 | 2 | 1 | 2 | 2 | 4 | 4 | 1 | 2 | 20 | 2.2 | MODERATE |
| Shin et al , 2019 | 4 |  | 4 |  | 2 | 4 | 1 | 1 |  | 16 | 2.7 | MODERATE |
| Shin et al, 2020 | 2 |  | 3 |  | 2 | 2 | 4 | 1 |  | 14 | 2.3 | MODERATE |
| Srisomboon et al, 2020 | 2 |  | 2 |  | 4 | 4 | 3 | 1 |  | 16 | 2.7 | MODERATE |
| Tai et al, 2006 | 2 |  | 3 |  | 2 | 2 | 2 | 4 |  | 15 | 2.5 | MODERATE |
| Uchida et al, 2017 | 2 | 3 | 3 | 2 | 3 | 4 | 4 | 1 | 3 | 25 | 2.8 | MODERATE |
| Wiesner et al, 2020 | 4 | 4 | 4 | 3 | 4 | 2 | 4 | 3 | 3 | 31 | 3.4 | HIGH |
| Wong et al, 2020 |  | 3 |  | 3 | 3 | 4 | 4 |  | 2 | 19 | 3.2 | HIGH |
| Wu et al, 2020 | 2 |  | 1 |  | 4 | 3 | 1 | 3 |  | 14 | 2.3 | MODERATE |
| Zaidman et al, 2017 | 2 |  | 2 |  | 3 | 2 | 1 | 1 |  | 11 | 1.8 | LOW |
| **Overall scores** | **2.4** | **2.9** | **2.8** | **2.8** | **2.5** | **3.0** | **2.8** | **1.8** | **2.6** |  | **2.6** | **MODERATE** |

**Supplementary Figure S1:** Overview of IL33 induction of Th2 cytokines. IL33 is released from epithelial cells and recruits ILC2 cells where it binds to the ST2 receptor. ILC2s then produce IL13, which can increase mucin production and induce airway hyperresponsiveness, IL4 which can induce the production of serum IgE, and IL5 which can recruit eosinophils to the lungs. Other cytokines which can be released from the epithelium, including IL6, IL25 and TSLP, can also induce ILC2s and Th2 cytokine production.


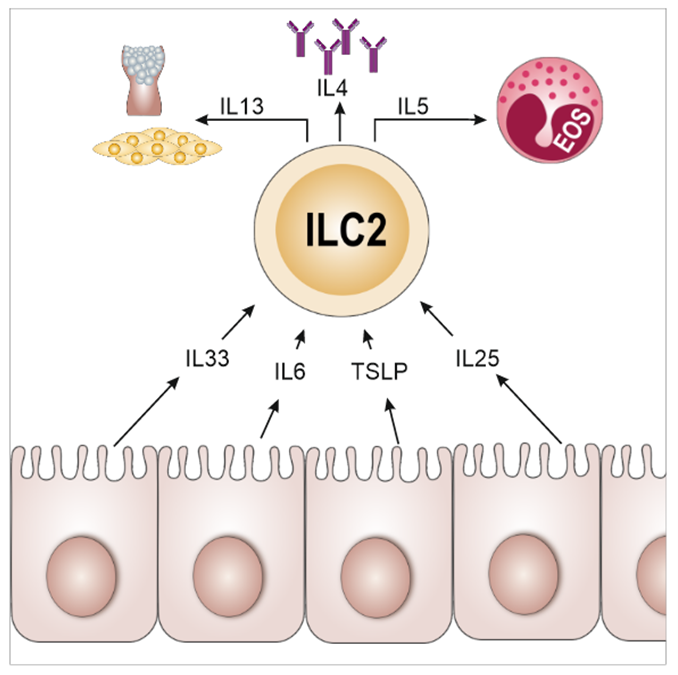

Supplement: Supplementary file 1 — Supporting Information S1 [file CLT2-13-e12252-s001.docx]
